# Functional genomics identifies five distinct molecular subtypes with clinical relevance and pathways for growth control in epithelial ovarian cancer

**DOI:** 10.1002/emmm.201201823

**Published:** 2013-05-13

**Authors:** Tuan Zea Tan, Qing Hao Miow, Ruby Yun-Ju Huang, Meng Kang Wong, Jieru Ye, Jieying Amelia Lau, Meng Chu Wu, Luqman Hakim Bin Abdul Hadi, Richie Soong, Mahesh Choolani, Ben Davidson, Jahn M Nesland, Ling-Zhi Wang, Noriomi Matsumura, Masaki Mandai, Ikuo Konishi, Boon-Cher Goh, Jeffrey T Chang, Jean Paul Thiery, Seiichi Mori

**Affiliations:** 1 Cancer Science Institute of Singapore, National University of SingaporeSingapore; 2 NUS Graduate School for Integrative Sciences and Engineering, National University of SingaporeSingapore; 3 Department of Obstetrics and Gynecology, National University Health SystemSingapore; 4 Division of Pathology, Norwegian Radium Hospital Oslo University HospitalOslo, Norway; 5 Faculty of Medicine, University of Oslo, Institute of Clinical MedicineOslo, Norway; 6 Department of Pharmacology, National University of SingaporeSingapore; 7 Department of Obstetrics and Gynecology, Kyoto UniversityKyoto, Japan; 8 Department of Hematology and Oncology, National University Health SystemSingapore; 9 Department of Integrative Biology and Pharmacology, University of Texas Health Science Center at HoustonTX, USA; 10 Institute of Molecular and Cell Biology, A*STAR (Agency for ScienceTechnology and Research), Singapore; 11 Department of Biochemistry, National University of SingaporeSingapore; 12 Division of Cancer Genomics, Cancer Institute of Japanese Foundation for Cancer Research3-8-31 Ariake, Koto-ku, Tokyo, Japan; 13 Present Address: Division of Cancer Genomics, Cancer Institute of Japanese Foundation for Cancer ResearchKoto-ku, Tokyo, Japan

**Keywords:** cell line model for subtype, functional genomic screen, molecular subtype, ovarian cancer, tubulin

## Abstract

Epithelial ovarian cancer (EOC) is hallmarked by a high degree of heterogeneity. To address this heterogeneity, a classification scheme was developed based on gene expression patterns of 1538 tumours. Five, biologically distinct subgroups — Epi-A, Epi-B, Mes, Stem-A and Stem-B — exhibited significantly distinct clinicopathological characteristics, deregulated pathways and patient prognoses, and were validated using independent datasets. To identify subtype-specific molecular targets, ovarian cancer cell lines representing these molecular subtypes were screened against a genome-wide shRNA library. Focusing on the poor-prognosis Stem-A subtype, we found that two genes involved in tubulin processing, *TUBGCP4* and *NAT10*, were essential for cell growth, an observation supported by a pathway analysis that also predicted involvement of microtubule-related processes. Furthermore, we observed that Stem-A cell lines were indeed more sensitive to inhibitors of tubulin polymerization, vincristine and vinorelbine, than the other subtypes. This subtyping offers new insights into the development of novel diagnostic and personalized treatment for EOC patients.

## INTRODUCTION

Epithelial ovarian cancer (EOC) is the most lethal gynaecologic malignancy. The global disease burden is approximately 225,000 new cases per year with a survival rate of 30% (Bray et al, 2013). EOC, like most other cancers, represents a heterogeneous collection of distinct diseases that arise as a consequence of varied somatic mutations and epigenetic changes acquired during the process of tumourigenesis and tumour progression. This heterogeneity is apparent in tumour histopathology such as serous, mucinous, endometrioid and clear cell histotypes. It is now established that the discrete histological types differ with respect to variable clinical features, including epidemiological risk, spread patterns, somatic mutations, chemotherapeutic response and patient prognosis (Gilks & Prat, 2009). The histologically distinct subtype, high-grade serous adenocarcinoma, is the most common subtype and accounts for approximately 70% of all ovarian carcinoma. Although this histotype has distinguishing clinical characteristics from the other subtypes, patients with this histological subtype still show diverse outcomes and usually low survival rates, even after the same or very similar treatment regimens (Gilks & Prat, 2009). One possible reason for this low survival rate is that the high degree of heterogeneity of EOC is not considered in the current standard of care (Vaughan et al, 2011). Thus, it is critically important to develop a systematic scheme to dissect the heterogeneity of EOC (Bast et al, 2009; Vaughan et al, 2011).

Genome-scale expression data has been instrumental in characterizing the complex biological diversity of human cancer (Alizadeh et al, 2000; Perou et al, 2000; Verhaak et al, 2010). Subtypes identified through expression microarray analyses are coupled with multiple clinical parameters, such as patient prognosis, age of onset and molecular marker expression (Alizadeh et al, 2000; Perou et al, 2000; Verhaak et al, 2010). Efforts to dissect EOC heterogeneity have correlated expression patterns with clinical features, such as histological types, aggressiveness and patient outcomes (Denkert et al, 2009; Helland et al, 2011; Mok et al, 2009; The Cancer Genome Atlas Research Network, 2011; Tothill et al, 2008). However, due to varied sample sizes and analytical criteria, the reported subtypes of EOC are similar but not completely the same (Helland et al, 2011; The Cancer Genome Atlas Research Network, 2011; Tothill et al, 2008; Verhaak et al, 2013), with reports of six molecular subtypes in 285 serous and endometrioid EOC (Tothill et al, 2008), yet only four molecular subtypes in 489 high-grade serous EOC (The Cancer Genome Atlas Research Network, 2011). Thus, a refined classification scheme with intense phenotypic characterization remains to be established. Also, the molecular targets relevant to cancer cell growth in these transcriptional subtypes have not been identified. The development of diagnostic and therapeutic strategies based on such a scheme is paramount for improving therapeutic efficacy in patients with EOC.

Despite recent successes with molecular targeted therapies for chronic myelogenous leukaemia, ER- or Her2-positive breast cancer, and EGFR-mutated lung cancer, targeted therapies for EOC have not been as encouraging (Quintas-Cardama et al, 2009; Rosell et al, 2010; Yaziji et al, 2004). One approach for the identification of specific targets for EOC subtypes is the use of a genome-wide, systematic, functional assessment of cancer cell growth (proliferation and/or viability). The recent success in suppressing the growth of cultured lung cancer cells with activating *EGFR* mutations by siRNA (Sordella et al, 2004) unveiled the sensitivity of siRNA-based approaches in distinguishing drivers of tumour growth. RNAi libraries, such as The RNAi Consortium (TRC) lentiviral library (Moffat et al, 2006; Root et al, 2006), have enabled systematic genetic studies in mammalian cells, and have identified the genes responsible for proliferation and viability in human cancer cell lines, particularly in the context of synthetic lethality (Barbie et al, 2009; Luo et al, 2008; Scholl et al, 2009).

The TRC library contains 80,000 lentivirally expressing short hairpin RNAs (shRNAs), corresponding to 16,000 human genes. In a systematic screen, a library such as this could be employed to help isolate key regulators of cancer cell growth on a genome-wide scale in a pooled format. Cultured cells would be infected with a pool of the shRNA-expressing lentivirus library such that a typical cell is subjected to only one integration event of an shRNA-expressing lentiviral genome into the host. Infected cells would then be allowed to proliferate for a period of time to permit the amplification or depletion of hairpins accordingly. Although the vast majority of shRNAs have minimal effects on cell proliferation and/or viability, an shRNA that silences the expression of a critical gene will be relatively depleted. Conversely, the relative amplification of an shRNA suggests that it targets a gene with an inhibitory role in cell growth. These integrated hairpins are then subsequently retrieved from the genomic DNA by PCR amplification, and the abundance of each shRNA sequence can be measured with microarray hybridization (Luo et al, 2008) or with next-generation sequencing technology (Sims et al, 2011).

Notably, the successful application of this platform led to the discovery of *PAX8* as having a more essential role in proliferation and survival in ovarian cancer cell lines than in cell lines from other tissues (Cheung et al, 2011). Furthermore, *TBK1* was identified as a synthetic lethal partner of oncogenic *KRAS* in an earlier report using this method (Barbie et al, 2009). Despite these successes, this technology has not been used to identify subtype-specific growth-promoting genes, particularly in the context of ovarian cancer.

Here, we describe a functional genomic approach to dissect the heterogeneity of EOC. We established a large-scale meta-analysis of EOC microarray datasets to determine EOC molecular subtypes. Next, we integrated EOC cell line data into the molecular subtyping scheme to derive an *in vitro* working model representative of each molecular subtype. Finally, we utilized genome-wide shRNA screening to identify molecular targets crucial for cell growth in a selected subtype, which linked the subtype with tubulin polymerization inhibitory drugs.

## RESULTS

### Molecular heterogeneity of epithelial ovarian cancer

We used a large collection of ovarian tumour gene expression data (*n* = 1538; serous: 1335, mucinous: 27, clear cell: 25, endometrioid: 96, and others: 55 samples; note that the histological distribution is largely biased toward serous adenocarcinoma as opposed to typical clinical setting) derived from 16 independent studies (Supporting Information Table 1) (Anglesio et al, 2008; Bild et al, 2006; Bowen et al, 2009; Denkert et al, 2009; Hendrix et al, 2006; Hogdall et al, 2003; Hsu et al, 2007; Iorio et al, 2010; Jochumsen et al, [Bibr b33],[Bibr b34]; Mok et al, 2009; Pejovic et al, 2009; The Cancer Genome Atlas Research Network, 2011; Tone et al, 2008; Tothill et al, 2008; Tung et al, 2009). Among the 16 datasets, the dataset from TCGA was the largest in sample number (*n* = 406; 26.4% of all samples). All publicly available datasets were included at the time of the study (April 2010), and compiled with an Oslo cohort dataset (BD and JMN). A strong batch-effect was removed by ComBat, eliminating technical differences across data collection sites, while conserving meaningful variations (Supporting Information Fig 1A and B) (Chen et al, 2011; Johnson et al, 2007). A preliminary statistical power analysis showed that 1500 or more samples were required to achieve sufficient statistical power (≥ 0.8) in capturing the complexity and dynamicity of EOC (Supporting Information Fig 2; Supporting Information Materials and Methods) (Fox & Mathers, 1997). In this collection, known prognostic factors were correlated with patient overall survival by univariate and multivariate Cox proportional hazards analyses (Table 1).

**Table 1:** Univariate and multivariate Cox proportional hazards regression analysis for multiple clinical variables and tumour subtypes

Clinical variables	Sample size (total *n* = 539)	Univariate (HR, 95% CI)	*p*-value	Multivariate (HR, 95% CI)	*p*-value
Age (year)					
<55	175 (32.47%)	1		1	
≥55	364 (67.53%)	1.403 (1.071–1.839)	0.0141	1.285 (0.9781–1.687)^a^	0.07173^a^
Stage					
I or II	47 (8.72%)	1		1	
III or IV	492 (91.28%)	3.907 (1.843–8.285)	0.00038	3.429 (1.591–7.389)^a^	0.00165^a^
Grade					
1	17 (3.15%)	1		1	
≥2	522 (96.85%)	2.58 (0.9578–6.949)	0.0608	1.365 (0.494–3.763)^a^	0.54799^a^
Metastasis					
Primary	500 (92.76%)	1		1	
Metastasis	39 (7.24%)	1.349 (0.8323–2.185)	0.224	1.391 (0.854–2.27)^a^	0.1853^a^
Subtype					
Non-Epi-A	483 (89.61%)	1		1	
Epi-A	56 (10.39%)	0.7103 (0.4498–1.122)	0.142	0.9449 (0.5834–1.53)^b^	0.8176^b^
Non-Epi-B	384 (71.24%)	1		1	
Epi-B	155 (28.76%)	0.69 (0.5206–0.9144)	0.0098	0.7347 (0.5532–0.976)^b^	0.033^b^
Non-Mes	361 (66.98%)	1		1	
Mes	178 (33.02%)	1.171 (0.907–1.513)	0.225	1.01 (0.7771–1.324)^b^	0.9164^b^
Non-Stem-A	411 (76.25%)	1		1	
Stem-A	128 (23.75%)	1.417 (1.075–1.868)	0.0135	1.382 (1.045–1.83)^a^	0.0234^a^
Non-Stem-B	517 (95.92%)	1		1	
Stem-B	22 (4.08%)	1.204 (0.6383–2.271)	0.567	1.14 (0.6033–2.149)^b^	0.6886^b^

Epi-A, epithelial-A; Epi-B, epithelial-B; Mes, mesenchymal; Stem-A, stem-like-A; Stem-B, stem-like-B.

*p*-values below 0.05 are shown in red.

a Multivariate Cox regression analysis of clinical variables with Stem-A subtype.

b For multivariate Cox regression, each subtype was independently analysed with the other clinical variables (age, stage, grade and metastasis) from the remaining subtypes.

**Figure 1:**
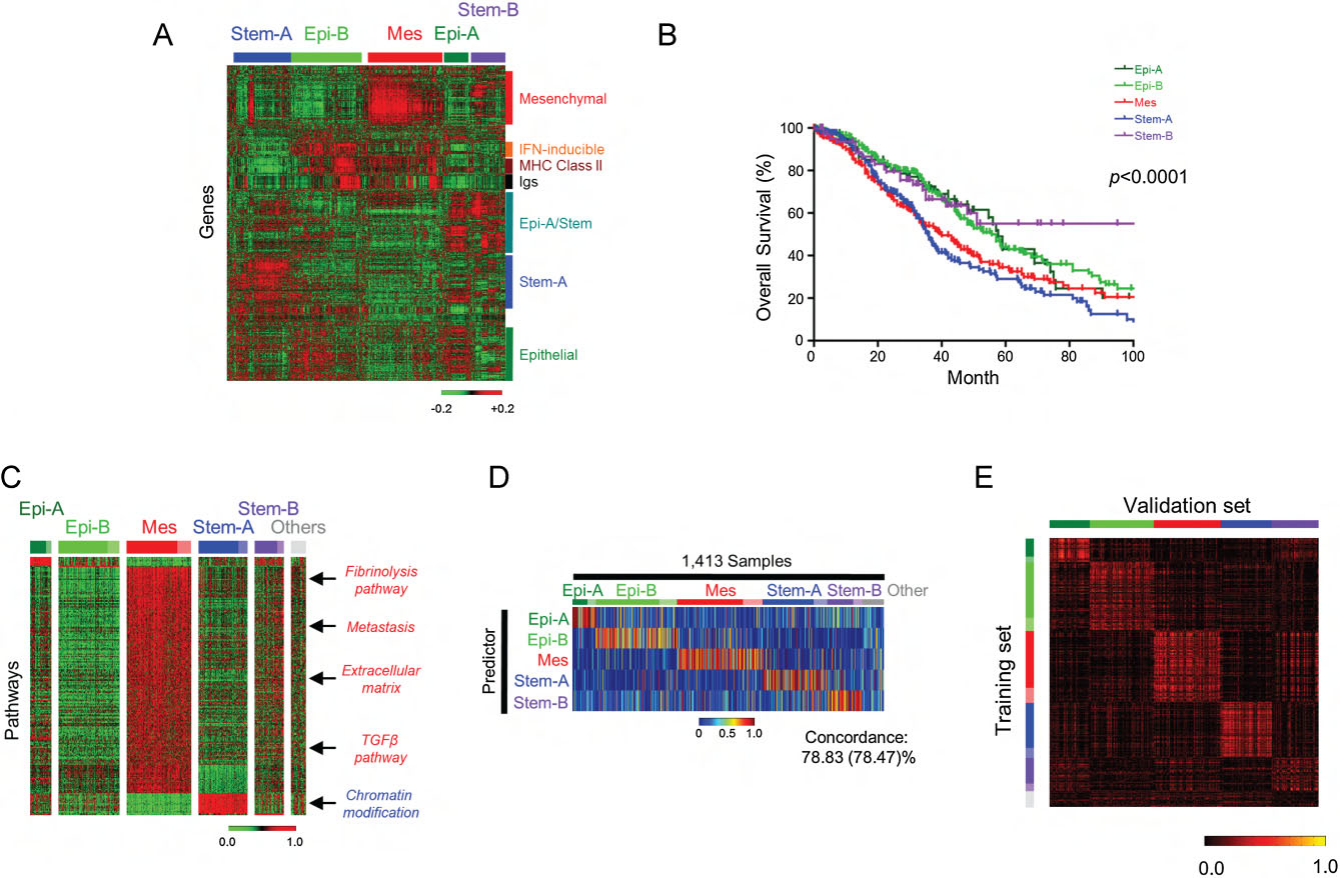
CC analysis revealed five subtypes of epithelial ovarian carcinoma Gene expression heatmap for the five tumour clusters (red = high; green = low expression). CC of 1538 samples identified five subtypes, designated by the associated gene components. Note the similarities between Epi-A/Stem-B subtype tumours, between Epi-A/Epi-B subtypes for epithelial genes, and the expression pattern of Epi-A/Stem genes. Also note that none of cultured cell-line data was included in this analysis.Kaplan–Meier survival analysis for each subtype. Among data for 1538 patient samples, survival information for 978 samples was available (GSE3149: 143, GSE9891: 277, TCGA: 400, GSE14764: 80, GSE18520: 53 and Oslo cohort: 25 samples) (Epi-A: 80, Epi-B: 264, Mes: 284, Stem-A: 220, Stem-B: 61 and others: 69 samples) and used for the Kaplan–Meier analysis.Subtype-specific pathway enrichment. Heatmap shows subtype-specific single sample gene set enrichment analysis (ss-GSEA) scores (false discovery rate (FDR) in significance analysis of microarrays (SAM) *q* = 0%, receiver operating characteristic (ROC) >0.85) for 1538 ovarian cancer samples. Red = high; green = low enrichment scores. Gene sets are aligned in descending value of ROC. Samples are aligned by subtype classification and SW. Deep colour = positive SW (core samples); pale colour = samples classified, but negative SW. “Others” indicates the unclassified samples not grouped in any of the five subtypes in the initial CC analysis in Fig 1. Arrows indicate positions of selected pathways.Ovarian cancer subtype predictors (BinReg). A heatmap is shown for the predicted probabilities of subtype status on 1413 clinical samples not used in the subtype predictor generation. Red = high; blue = low. Samples were aligned according to subtype classification by CC and SW. Colour as for (C). “Others” is represented as for (C).Heatmap of Spearman correlation *Rho* between the subtype of training data (*n* = 1538) and the BinReg predicted subtype of samples in five independent datasets (GSE19829, GSE20565, GSE30311, GSE26712 and GSE27651; total *n* = 418). The validation samples are aligned horizontally according to the predicted subtype, whereas the training samples are aligned vertically according to the subtype. Yellow = high correlation; black = low correlation. Abbreviations: Epi-A, epithelial-A; Epi-B, epithelial-B; Mes, mesenchymal; Stem-A, stem-like-A; Stem-B, stem-like-B.

To identify EOC molecular subtypes, we applied consensus clustering (CC) to the collection and detected five clusters (Fig 1) that were characterized by markers of differentiation or cell-type status and stromal components, including the presence of infiltrated inflammatory cells (Supporting Information Table 2). Subtypes were annotated by applying single sample gene set enrichment analysis (ss-GSEA) (Verhaak et al, 2010) with literature-curated gene signatures for epithelial, mesenchymal and stem cells (Supporting Information Text), and confirmed this characterization with the use of appropriate markers. The silhouette plot and SigClust (Liu et al, 2008b) analysis confirmed tumour similarity within each subtype, indicating the robustness of the classification (Supporting Information Fig 3A). The subtype distribution by cohorts and histology is presented in the Supporting Information Text and Supporting Information Figs 4A and B. Subtype distribution within the samples, taken by laser capture microscopy (GSE10971, GSE14407 and GSE18520), implied that the subtypes were intrinsic to cancer cells, and not dependent on stromal cells (Supporting Information Text).

We compared our subgrouping with a previous classification (285 samples; GSE9891) included in our combined dataset (Tothill et al, 2008). An overall concordance of 82.9% for all of the subtypes was found (Supporting Information Table 3; Supporting Information Fig 3B); thus, our large-scale analysis confirmed the previous study, and provided finer distinctions not detectable with fewer samples. Also, we noted that the proposed molecular subtypes were akin to that of serous ovarian carcinoma as proposed by The Cancer Genome Atlas Research Network (2011) (Supporting Information Fig 3B). However, the subtyping schemes from the previous studies did not show a one-to-one match with our proposed classification (Supporting Information Table 3; Supporting Information Fig 3B; Supporting Information Text; see the mutual relationships among Epi-A or Epi-B/C2, C3 or C4/Immunoreactive or Differentiated). This discrepancy may suggest a shared biological feature across these subgroups and hence may cause an imperfect distinction among the subtypes with predictive models as described later (Fig 1; Supporting Information Fig 8C; Supporting Information Table 8; Supporting Information Text). We also noted that TCGA molecular subtyping did not include a Stem-B/C6 population (Supporting Information Fig 3B; Supporting Information Text). The proposed subtypes in the current study are similar to the previously identified molecular subtypes yet reveal novel biological features.

### Correlation of subtype with clinicopathological parameters

We correlated the subtypes with various clinicopathological parameters to ascertain their clinical relevance (Supporting Information Fig 6A; Supporting Information Tables 4A and B; note that the clinicopathological information obtained with each dataset was neither standardized nor centrally reviewed across the datasets; therefore, there might be misdiagnosed or mis-evaluated samples included). We found a significant correlation between subtype and patient outcome: Epi-A, Epi-B and Stem-B subtypes had a better prognosis in a Kaplan–Meier analysis (Fig 1), while Mes and Stem-A tumours were linked with poorer outcomes. The Mes subtype included more advanced staged and metastasized tumours (Supporting Information Fig 6A; Supporting Information Tables 4A and B), whereas some Stem-A tumours were already found to be at stages 1 and 2 (Supporting Information Fig 6B), with poorer outcomes than those of other subtypes, even at stages 1 and 2 (Supporting Information Fig 6B), Furthermore, Stem-A tumours were enriched in older patients (Supporting Information Fig 6A; Supporting Information Tables 4A and B). The Stem-B subtype, on the other hand, was characterized by multiple histological types, including the majority of mucinous, endometrioid and clear cell carcinoma and some serous carcinoma (Supporting Information Figs 4B, 5 and 6A; Supporting Information Tables 4A and B). Focusing solely on serous tumours (Supporting Information Fig 6D), the frequency of Epi-A-classified tumours decreased significantly as tumour classification moved from serous tumours with low malignant potential (LMP) through to high-grade tumours, whereas the opposite shift in pattern was true for Mes and Stem-A serous tumours. All subtypes displayed high-grade serous carcinoma, with distinctions in survival in Kaplan–Meier curves (Supporting Information Fig 6C). The effect of molecular subtyping on prognosis was significant in both the univariate and multivariate Cox regression analyses with multiple combinations of clinically relevant parameters and status (Table 1; Supporting Information Tables 5A–E; Supporting Information Text).

Clear distinctions were also observed in the enrichment of the gene expression signatures for various pathways. The ss-GSEA analysis of 1538 samples using 6898 gene sets (GSEA databases Supporting Information Table 6) revealed a subtype-specific enrichment of 207 gene sets (Fig 1; Supporting Information Table 7) (Subramanian & Simon, 2011). Mes tumours correlated with *Metastases* and *TGF-β-related* pathways, consistent with their link with epithelial–mesenchymal transition (EMT) and metastasis (Supporting Information Fig 6A) (Maruyama et al, 2000; Yin et al, 1999). In comparison, *chromatin modification* gene sets were highly enriched in the Stem-A subtype (Fig 1; Supporting Information Table 7). Overall, this expression-based subtyping scheme dissected ovarian serous carcinoma heterogeneity into subgroups with similar biological properties.

### Predictive framework for EOC subtype classification

We next developed a predictive model with BinReg as a potential diagnostic tool for quantitative gene expression-based subgroup assignment (Supporting Information Fig 7A and B) (Gatza et al, 2010). This was performed using microarrays of representative samples for each subtype (*n* = 50 per subtype). Fig 1 shows predicted probabilities for subtype status of the remaining samples (*n* = 1413) not used in building predictive model. A comparison of the subtype predicted by BinReg with that classified by the CC (Fig 1) revealed an overall 78.8% concordance for all subtypes (78.5% for core samples) (Fig 1; Supporting Information Table 8), and a highly similar pattern of patient outcomes (Fig 1; Supporting Information Fig 7C). This demonstrated the powerful predictive capability of the method, with the concordance comparable with those reported in previous studies for multiple breast cancer cohorts (Supporting Information Text) (Calza et al, 2006; Haibe-Kains et al, 2012). We affirmed the accuracy of this method using 10-fold cross-validation (Supporting Information Figs 8A–C) (Blum et al, 1999; Kim, 2009; Konavi, 1995), 3-way split cross-validation (Ewens & Grant, 2001), and also by comparing BinReg to ClaNC (Supporting Information Fig 9; Supporting Information Materials and Methods).

To ensure the robustness of the classifier, we performed validation on five independent ovarian cancer datasets (total *n* = 418; Supporting Information Table 1) (King et al, 2011; Konstantinopoulos et al, 2010; Meyniel et al, 2010) that were not included in the prediction modelling. We observed high concordance for the gene expression patterns and clinicopathological characteristics in the predicted molecular subtype (Fig 1; Supporting Information Tables 4A, C and D). Using 260 samples from the validation set (GSE19829 [*n* = 28], GSE30311 [*n* = 47] and GSE26712 [*n* = 185]), for which patient outcome information was supplied (Konstantinopoulos et al, 2010), the Kaplan–Meier analysis on the BinReg-predicted molecular subtypes revealed a similar pattern of patient prognoses with that of the original CC analysis (*p* = 0.0372 by the log-rank test; Fig 1; Supporting Information Fig 7D) for subtypes other than Stem-B (Supporting Information Text). ClaNC (Dabney, 2006; Verhaak et al, 2010) further confirmed the highly comparable and predictive capability of this EOC subtyping (Supporting Information Fig 8D). Thus, the molecular subtype prediction model can assign clinical samples with unknown subtype status with high accuracy.

### Identification of representative cell lines for each subtype

**Figure 2:**
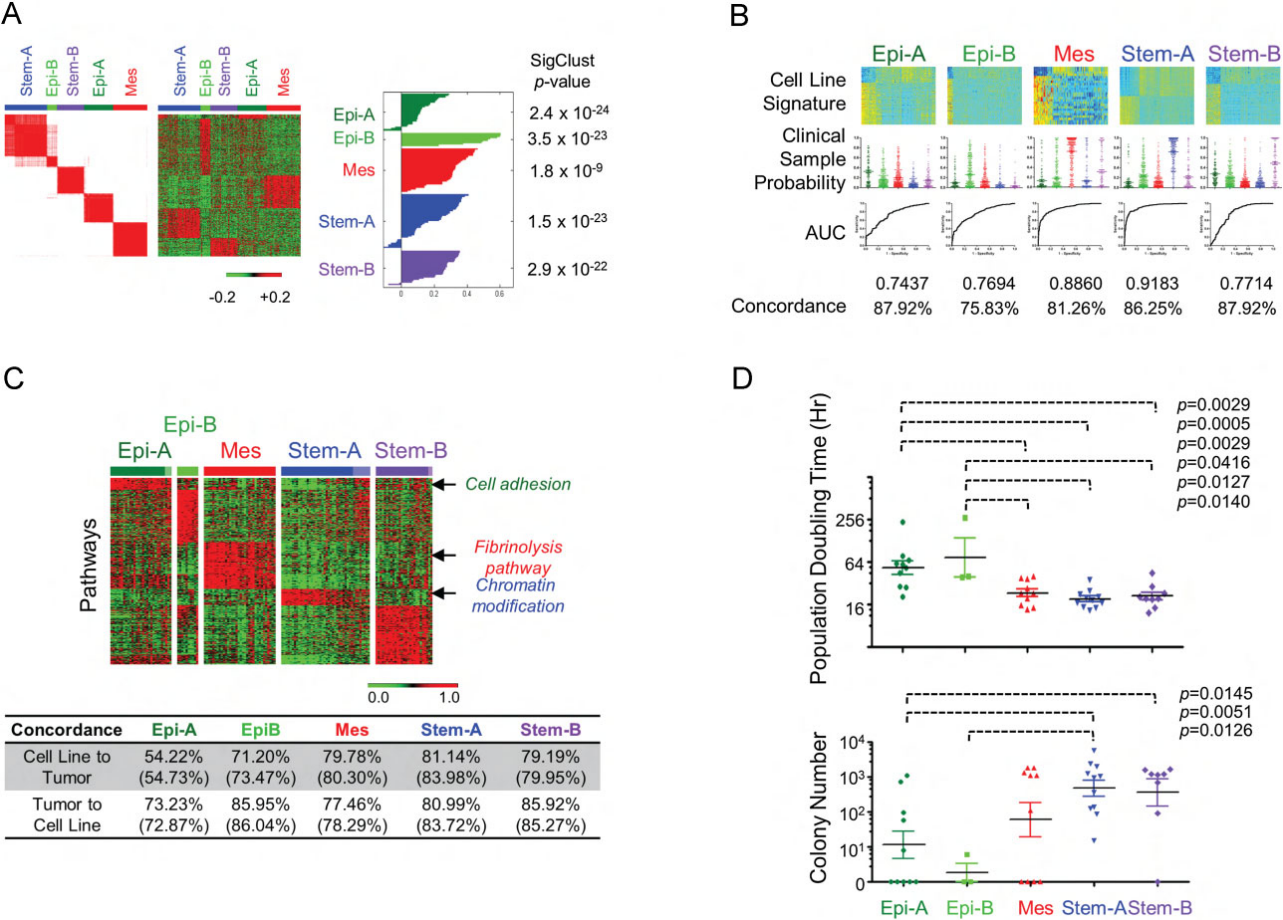
Identification of cell line subtype status Five subtypes in ovarian cancer cell line classification. Left panel. CC matrix of 142 ovarian cell lines. Red = high; white = low similarity. Middle panel. Gene expression heatmap of ovarian cell lines. Red = high; green = low expression. Right panel. Silhouette analysis for each subtype. Column to the right of silhouette plot is the SigClust (Liu et al, 2008b) *p*-value indicative of cluster significance for each subtype.Prediction of clinical samples by cell line predictors using BinReg. Upper panel. Gene expression heatmaps for subtype predictors based on cell line expression data. Red = high; blue = low expression. Middle panel. Predicted probability of core clinical samples for cell-line subtype predictor by BinReg. Each subtype signature detected the probability difference between the corresponding subtype from the remaining subtypes with statistical significance (*p* < 0.0001; Mann–Whitney *U*-test). Lower panel. Receiver operating characteristic (ROC) analyses of subtype predictors. Overall accuracy is shown by the area under the ROC curve (AUC) (Pejovic et al, 2009). Concordance (%) of the subtype status derived from CC with the prediction based on the cell line subtype predictors.Upper panel. Cell line subtype-specific pathway enrichment. Subtype-specific single sample gene set enrichment analysis (ss-GSEA) scores (false discovery rate (FDR) of the significance analysis of microarrays (SAM) *q* = 0%, ROC > 0.85 as overexpressed gene sets) for 142 ovarian cell lines are shown as a heatmap. Red = high; green = low enrichment scores. Gene sets aligned in descending value of ROC; samples are aligned according to the subtype classification by CC and the SW. Deep colour = positive SW (core samples); pale colour = samples classified to a subtype, but negative SW. Arrows indicate positions of selected pathways. Lower panel: Concordance (%) of the subtype status (from CC by genes) with the prediction result (from BinReg based on the subtype predictors by enrichment scores). The number in parentheses indicates the accuracy of the prediction against core samples.Characterization of *in vitro* phenotypes of cell lines in each subtype. Upper panel. Population doubling time of a cell line was measured with the MTS assay (Matsumura et al, 2011) and is shown as dot plots. Lower panel. Anchorage-independent cell growth ability for each cell line was measured using the methylcellulose assay (Mori et al, 2009). Log_10_-transformed colony number is shown. *p*-values were computed by Mann–Whitney *U*-test. Abbreviations: Epi-A, epithelial-A; Epi-B, epithelial-B; Mes, mesenchymal; Stem-A, stem-like-A; Stem-B, stem-like-B.

Cell lines corresponding to each EOC subtype were identified for *in vitro* modelling. We performed two rounds of CC on a pool of datasets from 142 cultured EOC cell lines, resulting in Epi-A: 29, Epi-B: 10, Mes: 34, Stem-A: 42 and Stem-B: 27 cell lines (Supporting Information Figs 10A and B); the results were unambiguously supported by similarity matrices, the silhouette values with significant *p*-value by SigClust (Fig 2) (Liu et al, 2008b), as well as consistent subtype assignments amongst biological replicates of 28 cell lines (Supporting Information Table 9; Supporting Information Text). The cell-line subtype predictors (Fig 2) were then applied to tumour core samples to estimate the molecular similarity of the subtypes between *in vivo* tumours and *in vitro* cell lines. We observed a high level of accuracy in the *a*rea *u*nder the *c*urve (AUC: 0.744 to 0.918) and a high concordance between the predicted tumour subtype by a cell-line classifier with the initially assigned tumour subtype (75.8–87.9%) (Fig 2). Furthermore, we found a high correlation between clinical tumour subtype and cell line subtype in the Spearman correlation map analysis (Supporting Information Fig 10C). These findings indicated a high level of similarity between ovarian cancer cell lines and tumour transcriptomic expression patterns (Fig 2; Supporting Information Fig 10C).

We next compared the pathway activation for these 142 cell lines with that of the clinical tumours using ss-GSEA analysis (Figs 1 and 2; Supporting Information Table 10). Epi-A cell lines were characterized by cell adhesion-related gene sets, reflecting enrichment of epithelial cell markers. Importantly, 33 of the 402 cell line subtype-specific gene sets were shared with tumours, including enrichment of *fibrinolysis pathway* and *chromatin modification* in the Mes and Stem-A subtypes, respectively (Supporting Information Table 10); this was confirmed with BinReg analyses using a statistical model with pathway enrichment scores (Fig 2). We estimated the subtype status of clinical samples by fitting a Bayesian probit regression model with the subtype-specific enrichment scores for cell lines. Reverse estimations were also performed from the tumour samples to the cell lines. By applying the same method as in Fig 2, we observed high levels of concordance between the predicted subtype of tumours by the cell-line ss-GSEA pathway classifier with the initially assigned tumour subtype (54.2–81.1%) and reciprocally high concordance between the predicted cell line subtype by a tumour ss-GSEA pathway classifier with the original cell line subtype (72.9–86.0%). These results indicated strong similarity between cell lines and tumours in the pattern of pathway enrichment (Fig 2). We then correlated the *in vitro* phenotypes for the molecular subtypes, and identified a significant correlation between cell line subtypes with population doubling time and anchorage-independent cell growth potential (Fig 2). Epi-A and Epi-B cell lines had longer population doubling times and decreased colony-forming ability, which may reflect the less-aggressive behaviour of clinical tumours. Overall, these cell lines can serve as good experimental models for each molecular subtype.

### Genome-wide shRNA screens identified subtype-specific growth-promoting genes

**Figure 3:**
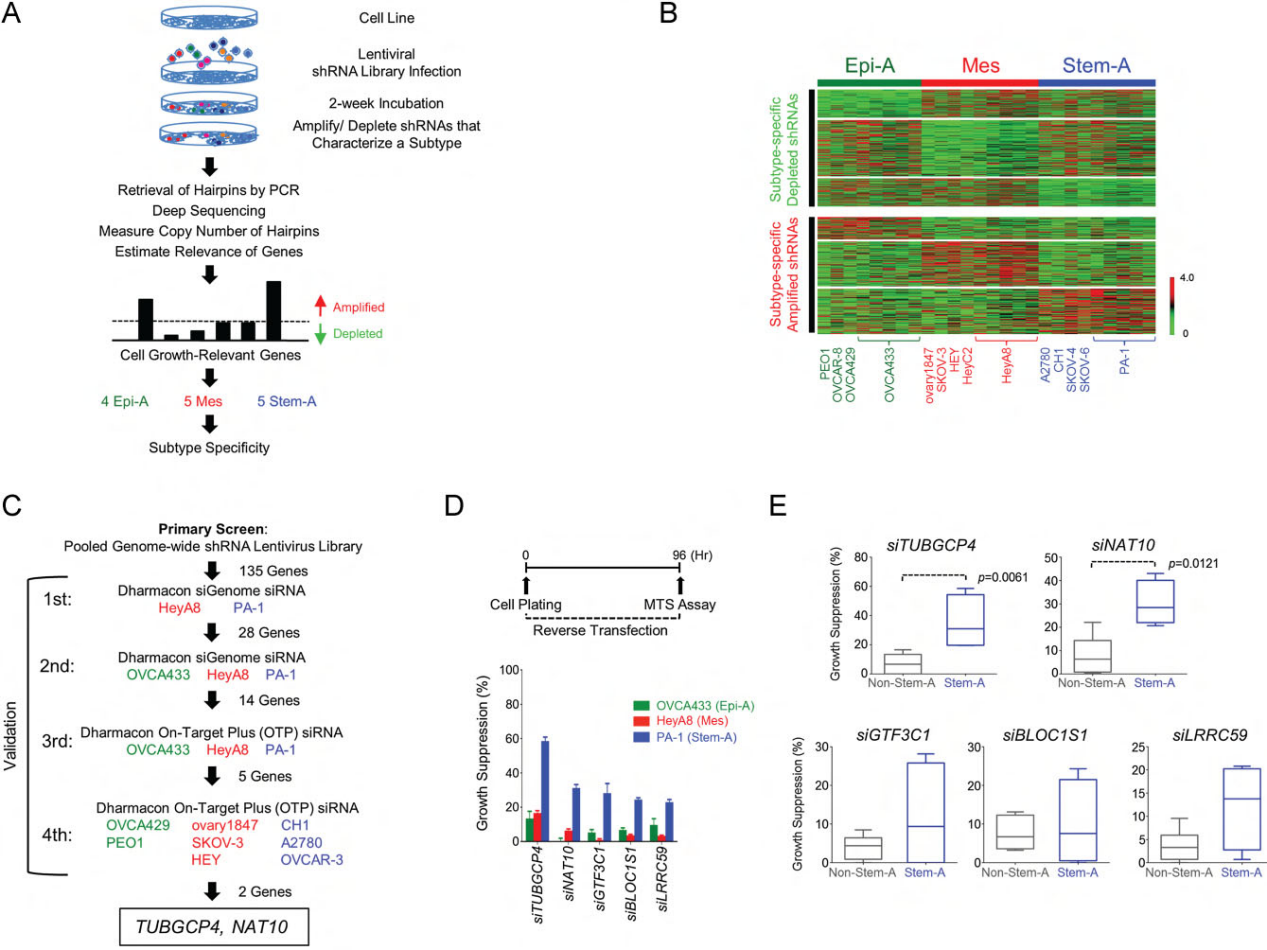
Subtype-specific functional relevance genes Schematic showing identification of functionally relevant genes for cell growth in a subtype-specific manner.Gene centred and normalized heatmap, compiled from two independent screens, shows hairpins selectively depleted or amplified in each subtype. The quadruplicates of three cell lines (OVCA433; Epi-A, HeyA8; Mes and PA-1; Stem-A) were assayed in the initial screen, while the second screen used one experimental replicate of 14 different cell lines (4 Epi-A: OVCA429, OVCAR-8, OVCA433, PEO1; 5 Mes: ovary1847, HEY, HeyA8, HeyC2, SKOV-3 and 5 Stem-A: A2780, CH1, PA-1, SKOV-4, SKOV-6). Using reads with a perfect match to the reference sequences (Sigma–Aldrich), the copy number of each hairpin was counted and normalized against the total number of reads in a sample and then rendered to RIGER analysis to find phenotype-specific, functionally relevant genes (Luo et al, 2008). Top panel. Subtype-specific depleted hairpins in Epi-A, followed by Mes and Stem-A subtypes. Each row represents shRNA hairpin copy number and is sorted according to the hairpin score identified in RIGER (Luo et al, 2008). Only hairpin scores ≥0.2 and genes significantly enriched in a subtype (*q* < 0.005) are shown. Bottom panel. Subtype-specific amplified hairpins arranged as in the top panel. Red = higher; green = lower copy number counts.Schematic of siRNA experiments validating the identified Stem-A-specific growth-promoting genes. This analysis led to the identification of two functionally relevant genes specific to Stem-A: *TUBGCP4* and *NAT10*.Validation of subtype-selective effect of the genes on cell growth by siRNAs. Upper panel. Timeline of assay performed for the siRNA reverse-transfection experiment. Lower panel. Effect of gene knockdown on cell growth (bar plots) as a percentage ratio of growth suppression, normalized against the negative controls. Error bar indicates the SEM of three independent experiments. Stem-A-selective growth suppression effect is shown for the inhibition of the five validated PA-1 (Stem-A)-specific growth-promoting genes in OVCA433, HeyA8 and PA-1, respectively. Green = OVCA433 (Epi-A); red = HeyA8 (Mes); blue = PA-1 (Stem-A).Effect of silencing PA-1 (Stem-A)-selective genes on cell growth in other ovarian cancer cell lines. The five PA-1-selective genes were silenced individually by siRNA in non-Stem-A (OVCA433, OVCA429, PEO1, HeyA8, ovary1847, SKOV-3 and HEY) and Stem-A (PA-1, CH1, A2780 and OVCAR-3) cell lines in three independent experiments, and examined for their effect on cell growth relative to the negative control. Averaged percentages of growth suppression in each group are shown as a box plot and were statistically evaluated using Mann–Whitney *U*-test with GraphPad Prism. Bottom, middle and top lines of each box represent the 25th percentile, median and 75th percentile, respectively, and whiskers extend to the most extreme values of the group. Inhibition with si*TUBGCP4* or si*NAT10* significantly suppressed cell growth of Stem-A cell lines as compared to non-Stem-A cell lines. Grey = non-Stem-A cell lines; blue = Stem-A cell lines. Abbreviations: Epi-A, epithelial-A; Mes, mesenchymal; Stem-A, stem-like-A.

Genes essential to each subgroup were investigated via genome-wide screens using the pooled TRC shRNA library, with the presumption that tumours within the same subtype would share molecular mechanisms for their growth (proliferation and/or survival). The experimental strategy of the screen is shown in Fig 3. Briefly, we conducted pooled shRNA screens on 14 ovarian cell lines, representing Epi-A, Mes and Stem-A subtypes, that differ profoundly in gene expression and clinical properties (Figs 1) (4 Epi-A: OVCA429, OVCAR-8, OVCA433, PEO1; 5 Mes: ovary1847, HEY, HeyA8, HeyC2, SKOV-3; and 5 Stem-A: A2780, CH1, PA-1, SKOV-4, SKOV-6). These 14 cell lines were selected based on their high silhouette width (SW) values for the subtype signature in order to screen with “more representative” cell lines for a given subtype, with a notion of PA-1 as a teratocarcinoma cell line (Supporting Information Table 11).

Two independent screens were performed to ensure reproducibility. The initial assay was designed to determine concordance among four experimental replicates of a single cell line per subtype (OVCA433, HeyA8 and PA-1 was used to represent Epi-A, Mes and Stem-A subtypes, respectively). Spearman correlations confirmed tight correlations among the quadruplicates in the screen (Spearman *rho* = 0.7528 ± SEM 0.0113, *p* < 10^−16^). The second screen was performed in 14 cell lines with the intention to detect differences across subtypes as well as concordance among different cell lines within a subtype. Since the screenings detected similarity in subtype-specific depletions or amplifications of hairpins, we combined both datasets and further performed RIGER analyses (Luo et al, 2008) on the compiled data. Supporting Information Fig 12A illustrates highly distinctive genome-wide patterns in the copy number of subtype-specific shRNAs that were depleted or amplified. The effect size was reasonably large (Cohen, 1988; Monk et al, 2012; Syrjanen & Syrjanen, 2013): the mean effect sizes of depleted hairpins were Epi-A = −0.9098; Mes = −0.7681 and Stem-A = −0.7818, and those of amplified hairpins were Epi-A = 0.8128, Mes = 0.8282 and Stem-A = 0.7486 (Supporting Information Fig 12B; Supporting Information Table 12).

The primary aim of the screens was to identify genes that, when inhibited, would render growth suppression on a certain molecular subtype. To this end, we identified depleted shRNAs targeting 77 genes for Epi-A, 85 genes for Mes, and 88 genes for Stem-A subtypes (Fig 3), with high significance in subtype enrichment (*q* < 0.005) and Hairpin Score (>0.2). These genes are potentially involved in growth promotion of the cells in a given subtype (Supporting Information Table 12). Conversely, we identified amplified hairpins targeting 43 genes for Epi-A, 72 genes for Mes, and 44 genes for Stem-A (Fig 3) that may have a suppressive effect on cell growth of the given subtype under conventional culture conditions (Supporting Information Table 12). For most of the growth-related functional genes, the abundance of shRNAs did not show significant correlation to gene expression, implying that the functional relevance of the genes was independent of their expression levels. Differences in experimental design and detection platforms hampered the integration of the results from this screen with that of another published screen using the same shRNA library (Supporting Information Materials and Methods) (Cheung et al, 2011).

### Validation of subtype-specific growth promoting genes

To validate the effects of the genes identified from the screens, we focused on the Stem-A subtype (given its worse clinical outcome) and targeted individual genes with siRNA (Fig 3). We chose 135 genes depleted in Stem-A subtypes based on a less stringent *q*-value cut-off of 0.03 from RIGER analysis (note that a more stringent *q*-value was used in Fig 3; Supporting Information Table 13). The validation of these 135 genes was performed in a process that consisted of four steps (Fig 3; with more details available in “Materials and Methods”) in order to identify siRNAs that inhibited growth on Stem-A cells but had a minimal effect on other cells. Stem-A-specific essential genes were identified as positive hits based on the following comparisons using Student *t*-tests: (1) comparison between the growth inhibitory effect of silencing the gene of interest with that of the siRNA negative controls in the Stem-A cells; and (2) comparison between the effect on Stem-A cells with that on the references for the subtype (non-Stem-A cells) (Fig 3). Relying on criteria of ≥20% growth suppression in PA-1 with *p* < 0.001 in a Student's *t*-test comparing control with the gene of interest and ≥20% growth suppression in PA-1 as compared with the reference cell line, 28 genes were found in the first step of validation to be selective for PA-1 cell growth (Supporting Information Table 13). In the second step, we examined the effect of these 28 genes in PA-1, HeyA8 and OVCA433, and further confirmed the growth suppressive effect of 14 of these 28 genes (Supporting Information Table 13). For the third step, we switched platforms from “siGenome” to “On-Target Plus siRNA” to further validate our observations using different sets of target sequences in the genes as well as to reduce possible off-target effects. After this step, five genes (*TUBGCP4, NAT10, GTF3C1, BLOC1S1* and *LRRC59*) were validated as PA-1-relevant genes (Fig 3). Importantly, PA-1 cells showed increased cleavage of Caspase-3 and PARP after treatment with si*TUBGCP4*, si*NAT10*, si*GTF3C1* or si*LRRC59*, indicating activation of apoptosis in these cells (Supporting Information Fig 13C). Finally, as the fourth step of the validation process, the experiments were conducted with use of additional non-Stem-A (Mes: ovary1847, SKOV-3 and HEY; Epi-A: OVCA429 and PEO1) and Stem-A (CH1, A2780 and OVCAR-3) cell lines to ensure its reproducibility and to exclude any possible impact of PA-1 cells being derived from a different cell-of-origin (teratocarcinoma), even though it had the highest SW of the Stem-A cell lines. *TUBGCP4* or *NAT10* siRNA treatment reproducibly resulted in a statistically significant reduction in cell growth for the Stem-A cell lines, while cell growth for non-Stem-A cell lines was not affected (Fig 3). These multiple stages of rigorous validation confirmed the dependence of Stem-A cell lines on *TUBGCP4* and *NAT10* in cell growth and ensured that this effect was not limited to PA-1 cells. Silencing of the other three genes (*GTF3C1*, *BLOC1S1* and *LRRC59*), albeit not statistically significant, also exhibited a tendency toward differential toxicity in Stem-A cells (Fig 3). These observations demonstrate that subtype classification based on gene expression is indeed mirrored by patterns of functional genetic determinants of cell viability. Moreover, the validated genes can provide us with an insight into the molecular mechanisms of Stem-A tumour growth.

### Microtubules as potent targets in Stem-A subtype

**Figure 4:**
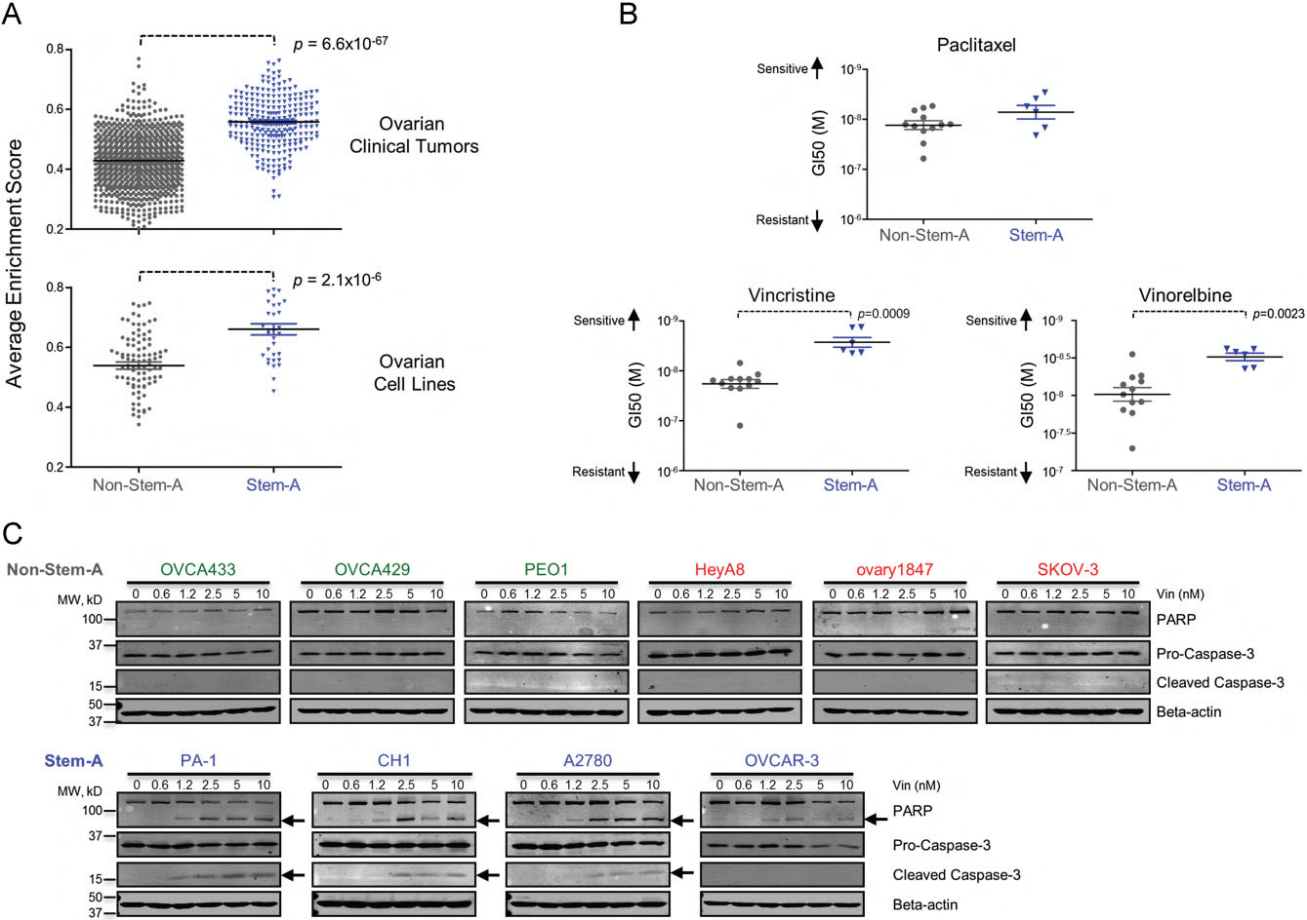
Susceptibility of Stem-A cells to microtubule assembly inhibitors Estimated microtubule activity in non-Stem-A and Stem-A subgroups of ovarian cancer. Microtubule activity in 1142 core samples of ovarian clinical tumours (Top panel) and in 129 core samples of ovarian cell lines (Bottom panel) was estimated based on the average single sample gene set enrichment analysis (ss-GSEA) enrichment score of 19 microtubule-related gene sets (Supporting Information Table 16) acquired from GSEA databases (Supporting Information Table 6). Differences in microtubule activity between non-Stem-A and Stem-A subgroups were statistically evaluated with Mann–Whitney *U*-test in Graphpad Prism. Grey = non-Stem-A subgroup; blue = Stem-A subgroup.Specificity of drug sensitivity in ovarian cancer cell lines. A panel of 18 ovarian cancer cell lines was classified into non-Stem-A (OVCA433, OVCA429, OVCAR-8, PEO1, OVCA432, OVCA420, HeyA8, HEY, HeyC2, SKOV-3, ovary1847 and DOV 13) or Stem-A (PA-1, CH1, A2780, OVCAR-3, SKOV-4 and SKOV-6) groups and analysed for their sensitivity to paclitaxel (Top panel), vincristine (Left bottom panel) and vinorelbine (Right bottom panel). GI50 values were calculated with the results from cell proliferation assays for each cell type in three independent experiments, and the mean GI50s are shown as dot plots. A non-parametric Mann–Whitney *U*-test in Graphpad Prism was used to evaluate the results statistically. A higher value along the *y*-axis indicates increased sensitivity to the drugs. Colour as for (A).Detection of apoptotic activity upon vincristine treatment. Six non-Stem-A (Upper panel) and four Stem-A (Lower panel) cell lines were subjected to increasing concentrations of vincristine (0 to 10 nM) for 48 h. The presence of apoptotic activity was determined by immunoblotting for cleaved PARP and Caspase-3, as indicated by arrows. Abbreviations: Stem-A, stem-like-A.

TUBGCP4 is a component of γ-tubulin ring complex, which is critical for nucleation of tubulin complexes in the cell (Fava et al, 1999; Moritz et al, [Bibr b54],[Bibr b55]). NAT10 is reported as a possible acetyl transferase of α-tubulin that may be involved in the stabilization of microtubules (Hubbert et al, 2002; Shen et al, 2009). The selective effect of si*TUBGCP4* or si*NAT10* on Stem-A cell lines (Fig 3) may suggest that the Stem-A cell lines are more susceptible to mitotic inhibition than other subtype cell lines. An examination of the expression data of clinical tumours and cell lines revealed higher activity in the enrichment score of microtubule/tubulin-related pathways for Stem-A than that for non-Stem-A subgroups (*p* = 6.6 × 10^−67^ and *p* = 2.1 × 10^−6^ by Mann–Whitney *U*-test, respectively; Fig 4; Supporting Information Table 16) (Verhaak et al, 2010). In addition, *TUBGCP4* knockdown resulted in a down-regulation of the *Microtubule* gene set in the transcriptome across Epi-A, Mes and Stem-A cell lines (Supporting Information Fig 13B; Supporting Information Table 14; Supporting Information Text).

These findings prompted us to examine the *in vitro* sensitivity of Stem-A cells to microtubule-targeted drugs such as paclitaxel, vincristine and vinorelbine using a panel of ovarian cancer cell lines (12 non-Stem-A: OVCA433, OVCA429, OVCAR-8, PEO1, OVCA432, OVCA420, HeyA8, HEY, HeyC2, SKOV-3, ovary1847 and DOV 13; 6 Stem-A: PA-1, CH1, A2780, OVCAR-3, SKOV-4 and SKOV-6). A growth inhibitory concentration of 50% (GI50; drug concentration for 50% growth inhibitory effects on cells) was measured for each cell line in at least three independent experiments. The Stem-A cell lines were found to be more sensitive to inhibitors of tubulin polymerization, vincristine and vinorelbine (Lobert et al, 1996), than non-Stem-A cell lines (Fig 4). In contrast, paclitaxel, a drug that stabilizes microtubules (Manfredi & Horwitz, 1984), resulted in no significant distinction between the two subgroups (Fig 4). Moreover, 48-h vincristine treatment caused apoptosis in Stem-A cell lines at 1.2 nM (Fig 4), whereas minimal or no apoptosis was observed in non-Stem-A cell lines, even at 10 nM concentrations (Fig 4). Taken together, these findings provide evidence that drugs targeting tubulin polymerization can be useful in treating patients with Stem-A EOC with poor clinical outcomes.

## DISCUSSION

Using a large collection of EOC samples, we identified five molecular subtypes (Epi-A, Epi-B, Mes, Stem-A and Stem-B) that exhibited distinct clinicopathological characteristics and rates of overall survival. Of these, Epi-B and Stem-A subtypes were found to be independent prognostic factors. We established a prediction model for these subtypes and validated this model on an independent dataset. For the first time, using a genome-wide shRNA screen, we found that subtype-matched cell lines have distinct vulnerabilities. In particular, the poor-prognosis Stem-A subtype exhibited elevated microtubule activity and was sensitive to several microtubule polymerization inhibitor drugs, such as vincristine and vinorelbine. These results offer possible therapeutic strategies to target specific subtypes of EOC.

Multiple clinicopathological parameters are linked with prognosis in EOC patients, such as age at diagnosis, peritoneal dissemination, metastasis to distant organs/lymph nodes, and response to platinum-based standard chemotherapy (Gilks & Prat, 2009). Here, we add transcriptional subtype as an additional prediction parameter. Although a correlation between the Mes subtype and patient prognosis was detected with a log-rank test, it was masked in the multivariate Cox analysis; this suggests that the Mes subtype may be confounded in the analysis because it is significantly enriched in tumours at a more advanced stage. Nevertheless, since Stem-A and Epi-B subtypes were detected as significant independent prognostic factors in both the univariate and multivariate analyses, this demonstrates the clinical importance of our classification scheme. Of note, a previous study of 489 samples could not correlate their molecular classification with patient overall survival, although a more recent study correlated two TCGA subtypes with relapse-free survival using the same cohort (The Cancer Genome Atlas Research Network, 2011; Verhaak et al, 2013). This is perhaps derived from a bias internal to the cohort, and suggests the need for a substantial number of samples, which is provided by combining multiple datasets, as presented here.

Genomic profiling aimed at dissecting the complexity of cancer could provide further opportunities for the identification of relevant molecular targets. However, a major challenge is to identify cell lines that reflect the relevant underlying tumour biology (Chin et al, 2011). Expression studies of cultured breast cancer cell lines have shown that *in vitro* cells retain subtype characteristics corresponding to those of their *in vivo* counterparts. Hence, matching breast cancer cell lines by expression data could represent *in vivo* tumours (Gatza et al, 2010; Neve et al, 2006; Perou et al, 2000). Whilst we acknowledge that cell lines may be divergent from their ancestral tumour and not wholly representative of the full diversity of ovarian cancer, we believe our classification represents a foundation for further development, particularly since ovarian cell lines can be assigned to unique ovarian tumour subtypes and are not derived from any random scheme. This concept is supported by the similarities in the expression and pathway activation between the cell lines and tumours of a given subtype, and could be further supported by shared cell functions, such as anchorage-independent cell growth and population doubling time. The availability of representative cell lines would facilitate the quest for functionally relevant targets and bring us a step forward in developing therapeutics that could be matched with the characteristics of individual patients.

Loss-of-function studies using pooled shRNA libraries have identified essential genes in specific human cancer cell lines in the context of synthetic lethality (Barbie et al, 2009; Luo et al, 2008; Scholl et al, 2009) and lineage-specificity (Cheung et al, 2011). Extending this concept, we utilized the pooled shRNA library, in combination with next-generation sequencing technology as the detection platform (Sims et al, 2011), to identify key subtype-specific regulators of cancer cell proliferation and/or survival. The relevance of such subtype-specific targets has been exemplified by *ESR1* (estrogen receptor α) for luminal-subtype breast cancers; these cancers share not only clinical features such as prognosis and the response to chemotherapy, but also the pattern of gene expression. *ESR1* has been used not only for diagnosis but also as a molecular target to treat cancer patients with this subtype (Howell, 2013; Sorlie et al, 2001). Importantly, in this study, specific growth determinants were distinguished amongst the ovarian cancer subtypes at the genome-wide as well as gene level. This observation supports the potential for subtype-specific therapeutic options in treating ovarian carcinoma and reinforces the clinical importance of the classification scheme proposed in this study.

Although the molecular mechanisms linking *TUBGCP4* or *NAT10* with Stem-A growth remains to be elucidated, susceptibility to vincristine and vinorelbine underscores the importance of tubulin polymerization in Stem-A cells. Both drugs are well-established chemotherapeutic agents that block cell proliferation by inhibiting microtubule assembly through its interaction with tubulin heterodimers (Lobert et al, 1996); however, they are not standard chemotherapeutic reagents for the treatment of EOC, unlike paclitaxel (Armstrong et al, 2006; McGuire et al, 1996). The molecules implicated in the tubulin polymerization pathway may provide us with a potential platform to more effectively target Stem-A ovarian cancer. As such, the survival of patients with ovarian cancer could be improved by the stratification and targeting strategy described in this study.

## MATERIALS AND METHODS

### Eligibility criteria and quality control of expression data

In order for our study to make broader generalizations and attain a larger sample size, reduced eligibility criteria were adopted (George, 1996). Female adult (age ≥20 years) patients with a clinical diagnosis of primary or metastatic ovarian cancer were included in our analysis. We imposed no limit on patient race, pre-treatment history or medical conditions, or on the stages, grades, and histology of the disease.

To control for the quality of expression data, we checked the quality of the Affymetrix chips (Affymetrix, Santa Clara, CA) using Bioconductor AffyQCReport package (Gautier et al, 2004) and the following criteria: average perfect-match (Neve et al, 2006) intensity, kernel density plot, GAPDH 3′:5′ ratio, β-actin 3′:5′ ratio, and centre of intensity for positive and negative controls. All chips passed at least one of the criteria, and hence, none of the samples was discarded.

### Data preprocessing of Affymetrix expression data

Ovarian cancer datasets were downloaded from multiple data repositories: Gene Expression Omnibus (GEO), Array Express, Expression Project for Oncology (ExpO), and The Cancel Genome Atlas (TCGA). Microarray data on Affymetrix U133A or U133Plus2 platforms were utilized for the analysis. Robust Multichip Average (RMA) normalization was performed on each dataset. ComBat (Johnson et al, 2007), a high precision and accurate technique for removing batch effect while conserving meaningful variation (Chen et al, 2011), was applied for batch adjustment on the compiled, normalized data. Removal of ovarian cancer cell lines, normal tissues and primary cultured normal cells from the batch-adjusted data yielded a dataset of 1538 ovarian tumour samples, predominantly composed by EOCs (Supporting Information Table 15A). Probes (1185) corresponding to 941 genes (Supporting Information Table 2) were retained by applying a threshold of standard deviation across samples >1.05. Expression values of selected genes were normalized and centred with Cluster 3.0 and further processed for subtype identification. An additional validation dataset of 418 samples were similarly collected and subjected to the same preprocessing procedure. Clinical information of the validation dataset is given in Supporting Information Table 15B.

### Consensus clustering

CC (Monti et al, 2005) using Gene Pattern software (Reich et al, 2006) was employed to identify robust clusters corresponding to the distinct subgroups in EOC. We chose hierarchical clustering with agglomerative average linkage, with Euclidean distance and a sub-sampling ratio of 0.8 for 1000 iterations. The condition of *K*_max_ = 18 was employed, as it gave a reasonable Gini index and purity of ∼0.8. “Other” was used to indicate the unclassified samples not grouped in any of the five subtypes in the initial CC analysis shown in Fig 1. They were not included in following statistical analyses for characterization of the molecular subtypes.

### Univariate and multivariate Cox regression analysis

From 845 samples with overall survival information, we extracted 537 samples from three institutions (GSE3149: 5, GSE9891: 241 and TCGA: 291) with clinical variables (Table 1). This information was transformed to binary information (presence/absence of a phenotype) prior to assessment of their prognostic association with overall survival by Cox proportional hazards regression analysis (Therneau & Grambsch, 2000). The same procedure was applied for Cox proportional hazards regression analysis for progression-free survival. We extracted 518 samples (GSE9891: 199 and TCGA: 319) from 596 samples with progression-free survival information. Univariate and multivariate Cox regression were performed using R (http://www.R-project.org). Multivariate analyses with clinical variables were conducted independently for each subtype.

### Statistical analysis for clinical parameters

GraphPad Prism was used to examine statistical significance of clinical stage, primary or metastatic tumours, histological subtypes, or the malignant potential of each subtype by Fisher's exact test. For Kaplan–Meier analyses, the statistical significance was calculated by log-rank test.

### Subtype-specific gene set enrichment

A total of 6898 gene sets were collected (Supporting Information Table 6). The ss-GSEA score (Verhaak et al, 2010) was computed to estimate the pathway activity for all 1538 ovarian cancer samples or 142 cell lines for each gene set. Based on the computed ss-GSEA score, a binary comparison was conducted for each subtype to identify subtype-specific pathway enrichment. Gene sets specifically and significantly enriched in a subtype were selected using SAM (FDR *q* = 0) and ROC (ROC > 0.85 as overexpressed gene sets).

### Predictive modelling and validation by BinReg

Expression data analysis, based on a binary regression model using the BinReg ver. 2.0, was described previously (Gatza et al, 2010). BinReg uses a Bayesian statistical analysis to fit a binary probit regression model on training data given a set of genes that are most correlated with the binary response/phenotype of interest (*e.g*. Epi-A *vs*. Non-Epi-A). The regression coefficients of these genes indicate the discriminating power of the genes and are weights for the overall meta-gene profile. The overall meta-gene profile is used for comparison and predicts the status of the phenotype of the new sample or dataset. In this study, we built a binary regression model for each subtype that singled out a subtype from the rest (*i.e*. Epi-A *vs*. Non-Epi-A) and adopted a divide-and-conquer approach for generating signatures for each of the different subtypes (Supporting Information Figs 7A and B). Briefly, the top 50 core samples were selected by their highest SW of all five subtypes, and subdivided into two sets of data: training set A and training set B. These training sets were utilized to determine appropriate parameters (Supporting Information Table 17; Supporting Information Materials and Methods) for the binary regression model. Subsequently, the condition was used to predict the remaining samples by training set A. To predict the status of the phenotype on a dataset, a Bayesian probit regression model was fit to assign the probability that the sample exhibited evidence of a phenotype, based on the concordance of its gene expression values with the signature (Gatza et al, 2010).

### Expression microarrays of cultured cell lines

Most of Duke, Kyoto and Singapore cell lines were derived from an original collection assembled in a Duke laboratory (Supporting Information Table 11) (Matsumura et al, 2011). Therefore, expression data for these 28 cell lines from the collection could be used as biological replicates. We extracted RNA from 34 cultured EOC cell lines (ovary1847, JHOS-2, OAW28, OAW42, OV7, OV17R, OV56, Caov-2, OV90, OVCA420, OVCA429, OVCA432, OVCA433, OVCAR-2, OVCAR-3, OVCAR-5, OVCAR-8, OVCAR-10, Caov-3, SKOV-3, UWB1.289, A2008, EFO-21, C13, OV2008, FU-OV-1, IGROV-1, TOV-112D, A2780, CH1, DOV 13, TYK-nu, PEO1 and COLO720E) (Methods: Cell line phenotypes *in vitro*) and performed expression assays with Affymetrix Human U133 Plus 2.0 arrays. The data were deposited in Gene Expression Omnibus (GEO) with the accession of GSE28724. Details of the EOC cell lines are given in Supporting Information Table 11.

### Cell line phenotypes *in vitro*

Cell lines were cultured in RPMI 1640 media (Invitrogen, Carlsbad, CA) with 10% foetal bovine serum (#S1810-500; Biowest, Nuaillé, France). Measurements of population doubling time and colony formation assays in methylcellulose were described previously (Huang et al, 2008; Liu et al, 2008a; Matsumura et al, 2011; Mori et al, 2009). Mann–Whitney *U*-test of GraphPad Prism was used to statistically evaluate the numerical values for the cell line phenotypes across the subtypes.

### Lentivirus library infection and shRNA retrieval by PCR of the genomic DNA

Fourteen cell lines representing Epi-A, Mes or Stem-A were chosen based on the SW for the subtype signature so as to have “more representative” cell lines for a given subtype, and these cell lines were used for shRNA screening. We used a pooled library of shRNA-expressing lentiviruses (80,000 clones targeting 16,000 genes per library, TRC1.0, #CSTVRS; Sigma–Aldrich, St Louis, MA). Optimal lentiviral infection conditions achieved a multiplicity of interest (MOI) of 0.3 to ensure the highest probability of having single shRNA integration into the host genome in each cell (Luo et al, 2008). Each lentiviral vector encodes each shRNA expression cassette with the puromycin resistance gene, allowing the use of puromycin to isolate stable integrants. Under selection pressure from puromycin (5 μg/ml), infected cells were allowed to propagate for ∼14 days (∼4 or 5 passages), whereby cells expressing shRNA that silence genes that were required for and known to suppress cell growth were depleted from and enriched in the culture, respectively. Hence, the abundance of each shRNA (=shRNA copy number) is reflective of the effect of an shRNA on cell growth. At the endpoint of the incubation, genomic DNA was harvested from the resulting cells by PureLink Genomic DNA kits (#K1820-01, Invitrogen). The integrated shRNA sequences were retrieved from the genomic DNA (100 ng) by PCR amplification using vector primers (shRNA Forward Primer: 5′-atcttgtggaaaggacgaaac-3′ and shRNA Reverse Primer: 5′-tactgccatttgtctcgaggt-3′) with KOD Plus ver. 2 (#KOD-211, Toyobo) and 28–32 cycles of 98°C for 10 s, 56°C for 30 s, and 68°C for 1 min. Products were purified with QIAQuick PCR Purification Kit (#28106, Qiagen, Hilden, Germany).

### Next-generation sequencing analysis by Solexa to count copy numbers of individual shRNAs

Amplified DNA (20 ng) from PCR was used to construct a sequencing library using a ChIP-Seq sample preparation kit (#IP-102-1001, Illumina, San Diego, CA). The two sample-multiplexing sequencing method was used individually, with multiplexing index 6 and index 12 primers for each sample (Illumina, #PE-400-1001). Constructed libraries were subjected to a final size-selection step on a 10% Novex TBE gel (#EC6275BOX, Invitrogen, Carlsbad, CA). DNA fragments of 205 bp were excised, recovered and quantified following Illumina's qPCR quantification protocol and guides. Quantified libraries were then sequenced on the Genome Analyzer IIx (Illumina) using the multiplexing single-end sequencing protocol at a length of 58 + 7 bp (#PE-400-2002, Illumina). Image analysis and base calls were performed using the default settings. After stripping off the PCR primer sequences, reads were then aligned to the shRNA library using Bowtie with the specified settings: –solexa1.3-quals -n 0-l 5 -v 0 -k 1 -m 1–best –strata -y –nomaqround. The data were deposited in GEO with the accession of GSE45420.

### Statistical identification of the functionally relevant genes in a subtype-specific manner

Using reads with a perfect match to the reference sequences (Sigma–Aldrich), copy number was counted and normalized by total number of reads in a sample. RNAi gene enrichment ranking (RIGER) was used to find phenotype-specific, functionally relevant genes from the scale-normalized copy number count data (Luo et al, 2008). Among 80,000 hairpins included in the library, next-generation sequencing analyses detected 60,002 and 65,533 shRNA hairpins in two independent screenings and 57,168 hairpins were intersected in both results. We compiled and subsequently standardized these two datasets by ComBat (Johnson et al, 2007). Binary comparisons were performed on the three subtypes (*e.g*. Epi-A subtype *versus* the others). We adopted the signal-to-ratio as the metric for ranking hairpins, 1000 as the number of permutations, and Kolmogorov–Smirnov in the RIGER settings. The false discovery rate was computed using the Benjamini and Hochberg procedure. Genes were considered significant at *q* < 0.005 in Fig 3 or *q* < 0.03 for the validation study. For heatmap presentation, we retained the hairpins with a hairpin score ≥0.2.

**The paper explained**

PROBLEM:

Epithelial ovarian cancer exhibits considerable heterogeneity, which may lead to poor survival rates for patients treated with standard chemotherapeutic regimens. This has prompted the need for a robust classification scheme to unravel this heterogeneity and allow for the development of personalized treatment strategies.

RESULTS:

A large collection of gene expression data enabled the identification of five distinct subgroups of ovarian carcinoma. The existence of these five subgroups was validated in an independent collection. Genome-wide shRNA screening against a panel of ovarian carcinoma cell lines revealed two subtype-specific targets and the pathways that control cancer cell growth.

IMPACT:

We identified five distinct subgroups, allowing rational patient stratification. Subsequent assays uncovered genes and deregulated pathways, which will be instrumental in guiding future therapeutic strategies for ovarian cancer.

### Validation of functional determinants in cell growth of Stem-A cell lines by siRNAs

We selected 135 genes as Stem-A-specific growth-promoting genes for further validation via siRNA transfection from the top hit gene list from RIGER analysis of shRNA lentivirus screens (*q* < 0.03). The validation experiments were performed via a process consisting of four steps (Fig 3). Dharmacon SMART pool siGENOME siRNA (1st and 2nd steps) and Dharmacon SMART pool ON-TARGET*plus* siRNA (OTP; 3rd and 4th steps) formats (Thermo Fisher Scientific, Lafayette, CO) were used to validate the effect of gene knockdown on cell growth of ovarian cell lines (Fig 3). PA-1 (1st, 2nd, and 3rd steps) and CH1, A2780 and OVCAR-3 (4th step) were used as representative cell line(s) for the Stem-A subtype. As reference(s) for the subtype, HeyA8 (1st step), HeyA8 and OVCA433 (2nd and 3rd steps), OVCA429, PEO1, ovary1847, SKOV-3 and HEY (4th step) were used (Fig 3). Cells were reverse-transfected with each individual siRNA per well in a 96-well format in the following conditions: OVCA433, 2500 cells with 0.3 μl of DF1 (T-2001); HeyA8, 800 cells with 0.08 μl of DF4 (T-2004); PA-1, 1200 cells with 0.22 μl of DF2 (T-2002); OVCA429, 1500 cells with 0.22 μl of DF4 (T-2004); PEO1, 4000 cells with 0.24 μl of DF4 (T-2004); ovary1847, 2500 cells with 0.12 μl of DF2 (T-2002); SKOV-3, 2500 cells with 0.12 μl of DF2 (T-2002); HEY, 1000 cells with 0.08 μl of DF4 (T-2004); CH1, 1800 cells with 0.17 μl of DF4 (T-2004); A2780, 2000 cells with 0.16 μl of DF1 (T-2001); OVCAR-3, 4000 cells with 0.2 μl of DF3 (T-2003, Thermo Fisher Scientific). We used two negative controls for Dharmacon SMART pool siGENOME siRNA transfection (#D-001206-13-20 and #D-001206-14-20), and one negative control for Dharmacon SMART pool ON-TARGET*plus* siRNA transfection (#D-001810-10-20). Assays were performed in quadruplicate. After 96-h incubation, an MTS assay was used to measure cell growth using a CellTiter 96 AQueous Non-Radioactive Cell Proliferation Assay following the manufacturer's recommendations (#G5430, Promega, Madison, WI). Genes were considered as Stem-A-specific growth-promoting genes when their down-regulation caused ≥20% growth suppression on the Stem-A cell line (*p* < 0.001), and showed ≥20% more growth suppression on the Stem-A line than on the reference cell lines.

### Cell line drug sensitivity *in vitro*

Eighteen ovarian cancer cell lines (12 non-Stem-A: OVCA433, OVCA429, OVCAR-8, PEO1, OVCA432, OVCA420, HeyA8, HEY, HeyC2, SKOV-3, ovary1847 and DOV 13; 6 Stem-A: PA-1, CH1, A2780, OVCAR-3, SKOV-4 and SKOV-6) were tested for their sensitivity to paclitaxel, vincristine and vinorelbine, as described previously (Bild et al, 2006). Paclitaxel (#T7402), vincristine (#V8879) and vinorelbine (#V2264) were purchased from Sigma–Aldrich. Cells were seeded in 96-well plates at an optimal density, which was determined for each cell line to ensure that it reached 80% confluency by the end of the assay. Following an overnight incubation, cells were treated with nine concentrations of each drug (twofold dilution series over a 128-fold concentration range) for 48 h. The percentage of the cell population responding to the drug relative to the negative controls was measured using a CellTiter 96 AQueous Non-Radioactive Cell Proliferation Assay, following the manufacturer's recommendations (#G5430, Promega). Dose-response curves were plotted using GraphPad Prism, to derive a growth inhibitory concentration of 50% (GI50; drug concentration for 50% growth inhibitory effects on cells) for each cell line in at least three independent experiments. Mann–Whitney *U*-test of GraphPad Prism was used to statistically evaluate the averaged GI50s between non-Stem-A and Stem-A cell lines.

### Western blotting analysis

Total cell lysates were prepared by direct lysis with RIPA buffer (#R0278, Sigma–Aldrich), supplemented with protease inhibitor cocktail (#539134, Calbiochem, Boston, MA). Protein concentrations were determined using BCA protein assay (#23225, Thermo Scientific, Rockford, IL). Electrophoresis of the cell lysates were carried out with a BioRad Mini Protean II apparatus and transferred onto PVDF membranes (#IPFL00010, Millipore, Billerica, MA) with a BioRad Mini Trans-Blot apparatus, following the manufacturer's recommendations. Membranes were immunoblotted with primary antibodies directed against PARP (#9542, Cell Signaling, Danvers, MA), Caspase-3 (#9662, Cell Signaling) or β-actin (#A1978, Sigma–Aldrich), followed by immunoblotting with secondary IRDye 800CW conjugated goat anti-rabbit (#926-32211) or IRDye 680 conjugated goat anti-mouse antibodies (#926-32220, LI-COR Biosciences, Lincoln, NE). The western blots were scanned using an Odyssey Infrared Imaging System from LI-COR Biosciences.

## For more information

National Library of Medicine (Ovarian Cancer): http://www.ncbi.nlm.nih.gov/pubmedhealth/PMH0001891/

RNAi Consortium: http://www.broadinstitute.org/rnai/trc/lib

BinReg http://dig.genome.duke.edu/software.html

http://www.stat.duke.edu/∼mw/ABS04/Lecture_Slides/5.Stats_BinReg.pdf

ClaNC http://www.stat.tamu.edu/∼adabney/clanc/

RIGER http://www.broadinstitute.org/cancer/software/GENE-E/

## Supplementary material

As a service to our authors and readers, this journal provides supporting information supplied by the authors. Such materials are peer reviewed and may be re-organized for online delivery, but are not copy-edited or typeset. Technical support issues arising from supporting information (other than missing files) should be addressed to the authors.
