# Supplementary material for: Functional genomics identifies five distinct molecular subtypes with clinical relevance and pathways for growth control in epithelial ovarian cancer
Source: EMBO Mol Med. 2013 May 13;5(7):983–98. doi: 10.1002/emmm.201201823 (PMC3721473; doi:10.1002/emmm.201201823)
Supplement: Supplementary file 1 [file emmm0005-0983-SD1.pdf]

EMBO Molecular Medicine

Functional Genomics Identifies Five Distinct Molecular Subtypes with Clinical Relevance and Pathways for Growth Control in Epithelial Ovarian Cancer

Tuan Zea Tan, Qing Hao Miow, Ruby Yun-Ju Huang, Meng Kang Wong, Jieru Ye, Jieying Amelia Lau, Meng Chu Wu, Luqman Hakim Bin Abdul Hadi, Richie Soong, Mahesh Choolani, Ben Davidson, Jahn M Nesland, Ling-Zhi Wang, Noriomi Matsumura, Masaki Mandai, Ikuo Konishi, Boon-Cher Goh, Jeffrey T. Chang, Jean-Paul Thiery, Seiichi Mori

Corresponding author: Jean Paul Thiery, Proteos

Review timeline:

Submission date:	06 August 2012
Editorial Decision:	26 September 2012
Revision received:	18 February 2013
Editorial Decision:	19 March 2013
Revision received:	03 April 2013
Accepted:	09 April 2013

Transaction Report:

(Note: With the exception of the correction of typographical or spelling errors that could be a source of ambiguity, letters and reports are not edited. The original formatting of letters and referee reports may not be reflected in this compilation.)

Editor: Anneke Funk / Céline Carret

1st Editorial Decision 26 September 2012

Thank you for the submission of your manuscript "Functional Genomics Identifies Five Distinct Molecular Subtypes with Clinical Relevance and Pathways for Growth Control in Epithelial Ovarian Cancer" to EMBO Molecular Medicine and please accept my apologies for the delayed reply. We have now received the reports from two the three referees whom we asked to review your manuscript and since the review process so far has been lengthy, we prefer to take a decision now and forward the third report if/when it comes in.

You will see that the reviewers find the topic of your manuscript potentially interesting. However, they also raise significant concerns on the study, which should be addressed in a major revision of the manuscript.

Importantly, both Reviewers highlight, in addition to a number of technical concerns, that more mechanistic insight should be provided concerning the pathways that differentiate the subsets. In addition, Reviewer #1 notes that more detailed clinical information has to be provided and that the cell line classification has to be improved.

Given the balance of these evaluations, we feel that we can consider a revision of your manuscript if you can convincingly address the issues that have been raised within the time constraints outlined below.

However, we realize that addressing all of the referees' criticisms might require a lot of additional time and effort and be technically challenging. I am uncertain whether you will be able (or willing) to return a revised manuscript within the 3 months deadline and I would also understand your decision if you chose to rather seek rapid publication elsewhere at this stage.

Should you decide to embark on such a revision, revised manuscripts should be submitted within three months of a request for revision. They will otherwise be treated as new submissions.

Should you find that the requested revisions are not feasible within the constraints outlined here and choose, therefore, to submit your paper elsewhere, we would welcome a message to this effect.

***** Reviewer's comments *****

Referee #1 (Comments on Novelty/Model System):

Insufficient details of the sample cohorts are included; The PA-1 cell line representing "Stem-A" was derived from human ovarian teratocarcinoma cells which is not EOC but a germ cell tumor (Zeuthen et al. Int J Cancer 1980).

Referee #1 (General Remarks):

This study presents a large meta-analysis of epithelial ovarian cancer (EOC) microarray datasets in which five molecular subtypes with different characteristics are identified. A collection of 16 microarray datasets was used as a training set, and combined with a collection of 5 microarray datasets for validation. The successful clustering of multiple data sets is remarkable as a batch effects are common and approaches such as ComBat tend to remove not only batch effects, but also biological variation. Finally, a genome-wide shRNA screening method is utilized to identify subtype-related growth-promoting genes in selected molecular subtypes, and validated five genes as "Stem-A" related growth-promoting genes using PA1 cell line. A pathway analysis to suggest an association between mitochondrial metabolic pathways and "Stem-A" cell proliferation.
Generally, the manuscript should be more realistic about the results and be put more clearly in the context of previous findings; there is a general tendency for overstating the findings that aims to convince the reader of the importance of the study. This is unnecessary. Throughout the Results section the logic of why certain choices were made or how to interpret certain results needs to be more clear. A lot of insider's knowledge is expected from the reader. The manuscript represents a large body of work with a potentially exciting end result - possible involvement of energy metabolism in a specific subset of EOC - but it leaves the reader with an a bit of an empty feeling as the end result is entirely speculative. The study would greatly benefit from an attempt to show that energy metabolism is what sets the StemA subset apart from the other subtypes. Without followup to this result, it remains unclear what the true contribution of this study is, specifically of the shRNA/siRNA screens.

Detailed comments are described below.

1. "Computation of entropy showed that 1,500 or more samples were required to capture the complexity and dynamicity of EOC (Suppl. Fig. 1B) (Ewens & Grant, 2001)." Fig 1B shows that about 500 samples is likely to capture the major entropy.
2. As was reported by TCGA, serous ovarian carcinoma can be separated into four subgroups that were very similar to the serous ovarian carcinoma subgroups of Tothill et al. See also the review by David Bowtell in Nat Rev Cancer, 2010. These results should be acknowledged in the introduction. Supplemental Table 3 shows that the clusters reported here are again very similar to Tothill's. This similarity is recognized and the authors need to be careful not to overstate the novelty of the subgroups reported throughout the manuscript. For consistency, the authors should compare the names they propose for their subtypes to those proposed by Tothill (c1-c6) or TCGA

(differentiated/c4, mesenchymal/c1, immunoreactive/c2, proliferative/c5); they should strongly consider to use these previously proposed naming conventions for consistency.
3. A breakdown of the subgroups by data set as well as by histology should be provided to allow full interpretation of the clustering results. The authors use not only high-grade serous types but also other histologic types that share few molecular similarities (Zorn et al. Clin Cancer Res 2005; Vaugen et al. Nat Rev Cancer 2011). Especially, most invasive mucinous ovarian cancers are metastases from the gastrointestinal tract to the ovary. Therefore, the authors have to be especially careful about the interpretation of the results when molecular subtypes are identified in EOC. Although the authors showed clinical information in Supplementary Fig. 2, the details of clinical information per subtype are unknown. More details of clinical information per subtype in the training and validation datasets should be provided as a Table.
4. It should be briefly explained what the BinReg tool is and does. 78.8% classification accuracy of this approach seems low and unconvincing and it surprising that the core samples perform worse (78.5%) than non-core samples. It should be explained why the results of combined clustering of validation data set and training data set to find the subtype of samples in the validation data set, was used over the more conventional approach of using a classifier (in this case ClanC). Similarly, why were cell lines not classified (using ClanC or another classification method) but clustered. It is surprising to find cell lines of the class that resembles Tothill's c2, as the c2 class was related to tumor associated T-lymphocytes that are not part of any cell line model.
5. An association between molecular subtypes and prognosis is observed and referred to in the discussion as well as the results. This result should be discussed in the context of covariates such as surgical status (or status of residual tissue), which is one of most important prognostic factor in EOC (deBois et al. Cancer 2009). Progression-free survival time (or time to recurrence) should be included. The Kaplan-Meier survival curves (Fig. 1B and Suppl. Fig. 3D) of training and validation sets suggest a difference in prognosis of "Stem-B" subtype, which includes many samples with non-serous histologic types. This suggests that the outcome differences observed are due to the inclusion of different histologies.
6. It is suggested that the EOC cell lines mimic the clinical outcome behavior of the primary tumor samples. Does this result hold up when only including serous tumors into the analysis, or are the differences observes due to different histologies and the inclusion of LMP cell lines.
7. How were the 14 celllines used for shRNA screen selected? What was the histology of the celllines? The term 'shRNA copy number' was confusing as copy number is often used to reflect DNA dosage; similar for the terms deleted/amplified. What does it mean when an shRNA is depleted/amplified? Is the target gene than suggested to be a growth inhibitor/stimulator? Not all readers will understand these details.
8. There should be more details on the implementation of the shRNA screen in the main text. For instance, details on how the 'shRNA copy number' is assessed on compared between different shRNAs and celllines would be helpful in interpreting the result. How many genes are targeted by the library and are the numbers of identified shRNAs/genes more than expected by random chance? How many genes were deleted/amplified in all celllines? Although the subtypes are a interesting characteristic of EOC, it is not clear why those genes that are different between sutbypes would be the most disease-relevant, rather than genes associated with all celllines. Figure 3B shows a heatmap of the identified shRNAs in each of the celllines and the data is likely (z-score) normalized. The effects of this normalization should be explained, as the normalization will force the data to show (visually attractive) differences.
9. Why is the number of genes depleted in Stem-A (n=135) different from what is reported in the previous paragraph (n=88)? The PA-1 cell line representing "Stem-A" was derived from human ovarian teratocarcinoma cells which is not EOC but a germ cell tumor (Zeuthen et al. Int J Cancer 1980). This is likely to have an impact on the results and suggests that the cellline classification will be able to classify any expression profile and is not EOC specific .
10. The logic and flow of the "Validation of subtype-specific growth promoting genes" section is unclear. Was the HEYA8 cellline used a control in the t-test comparison? The identification of five genes as growth-promoting seems adequate, but is hard to interpret without better understanding what the control was.
11. Were technical replicates performed for each cellline-shRNA expression array experiment? For any of such experiments, triplicates are adviced as a minimal number by any statistical model.

Minor comments

1. Paragraph 1: The authors should provide the eligibility criteria in this study including quality control of expression data.
2. Materials and Methods/Supplementary Table 11: The authors should describe source of information (for example GEO_ID) about the Duke Cohort dataset.
3. Materials and Methods, paragraph 7: The authors should describe the name of cell lines precisely. Ex) Tyknu -> TYK-nu
4. Supplementary Table 1: The column of "Genomic data" is unnecessary because the authors didn't use genomic data except expression data. Instead, the authors should add a column of "chemotherapy".

Referee #3:

The manuscript "Functional Genomics Identifies Five Distinct Molecular Subtypes with Clinical Relevance and Pathways for Growth Control in Epithelial Ovarian Cancer" by Tan, Huang and Miow et al. identified gene expression signatures associated with five subtypes of epithelial ovarian cancer (EOC). The authors analyzed the largest compilation of EOC expression profiles to date and defined molecular subgroups which display unique clinical outcome. The authors extend their EOC molecular subgroup expression profiles to traditional in vitro cell lines which allowed for their classification as clinically relevant models. Through the use of RNAi libraries, the authors identified genes that affect EOC sub-type specific tumor cell growth and suggest the biological pathways that may underlie these sub-type dependencies. The manuscript presents extensive data and analyses including an expression meta-analysis of a very large number of ovarian cancers, and genome-wide shRNA data for 14 ovarian cancer cell lines. However, some of the analyses could be improved, and many of the conclusions seem overstated.

Major Concerns:

1) The subtyping analyses at times seem to be circular. For example, tumor assignments were determined for external datasets by applying BinReg using a predictive model generated from the internal data. These results were compared to "true" subtypes determined by pooling the external data with 1,142 samples from the internal data. In both cases, the internal data will dominate the analysis and determine similar cluster assignments.

Likewise, the internal data were divided into deciles with leave-10%-out determination of the cluster assignments; these assignments were then compared to the assignments using all the data. Again, the 90% of the data used in both analyses will dominate the cluster assignments, which will necessarily overlap as a result. A better approach would seem to be to divide the data into, for example, 40%, 40%, and 20%, then use each 40% independently to classify the 20%, and evaluate concordance.

Similarly, the tumor data were used to determine a classification scheme for the cell lines, and the resultant subtype assignments were compared to assignments obtained from clustering of all the tumors and cell lines together. Again, the concordance of the results simply reflects the fact that substantially the same data were used to determine the clustering pattern in both analyses.

2) I do not understand the justification or process for classifying cell lines based on two rounds of consensus clustering. It would seem that CLANC could just be applied based upon the original clustering results.

3) On p. 13, "Importantly, our shRNA screens detected specificity in the shared pattern within a subtype in the depletion or amplification of shRNAs." Yet it is not clear that the depleted/amplified shRNAs were significant when p-values are corrected for multiple hypotheses. One straightforward way to validate the data (beyond the individual hairpins evaluated in single lines in the following

experiments) would be to compare the results to previously published genome-level shRNA screens in ovarian cancers, eg Cheung et al, PNAS 2011.

Also, I would be interested to know the effect sizes of these hairpins (not just the ones explored in detail in following experiments). RIGER was applied using a KS statistic, which is valid but may highlight hairpins with small (though consistent) effect sizes.

4) The biological evaluation of the "Stem-A specific growth promoting genes" is limited in scope. In particular, the GSEA results implicating pathways are suspect because they represent comparisons of individual cell lines. Moreover, the authors conclude that finding "Mitochondria Membrane" etc as restricted to the response of the Stem-A line to shRNA to the 5 genes suggests a link between these pathways and cell proliferation. Rather, these pathways are likely to reflect effects of toxicity: namely activation of apoptotic pathways and cell death. Moreover, the Discussion paragraph on these pathways is very speculative. We need much more mechanistic data before we can implicate these pathways as therapeutic targets, and would have to at least try drugs targeting these pathways on the cell lines before proposing them for clinical trials.

5) The manuscript defines 5 molecular subtypes of EOC. How do these subtypes compare with the 4 transcriptional subtypes described by the TCGA? Does your 5th subgroup subdivide a TCGA subtype, or does it represent a novel subtype?

6) The clinical utility of the molecular subtypes is emphasized, but it is not clear to me that this is justified by the modest magnitude of difference in outcomes between the subtypes. Moreover, in the Discussion the authors state "we believe our classification represents a foundation for further development, particularly since ovarian cell lines can be assigned to unique ovarian tumor subtypes." It is not surprising that cell expression profiles could be so assigned-a random subtyping scheme would also seem to divide the cell lines. It would be more interesting if the subtypes predicted other characteristics of the cell lines that were similar to tumors, eg genetics. The authors mention anchorage-independent growth and growth rate, but these are not measured in tumors.

Minor Concerns:

1) On page 6, "Computation of entropy showed that 1,500 or more samples were required..." suggests that there is a clear cutoff at 1,500, but I see little change past 500.

2) On page 7, "non-serous EOC appears to have a distinct molecular profile"-the data do not convincingly support this. Both the non-serous and (particularly) serous tumors appear to reside in several clusters, and no cluster contains only non-serous tumors.

3) In Supp Fig 1, it would be useful to see a heatmap of all the data, arranged by dataset, to evaluate the effectiveness of ComBat.

4) Supp Fig 2 is small and difficult to read. The equations in the Entropy analysis are also illegible in my copy.

5) ss-GSEA of EOC samples resulted in numerous gene sets enriched for each subtype. On page 7 the authors state, "Mes tumors correlated with metastases and TGFB-related pathways". Yet the Mes tumors were enriched for 177 gene sets/pathways. Is it correct to connect Mes tumor metastatsis to a single gene set if there are a multitude of gene sets enriched?

1st Revision - authors' response 18 February 2013

Referee #1 (Comments on Novelty/Model System):

1. Insufficient details of the sample cohorts are included;

> In order to maximize the utility of the sample cohorts in this study, we have added clinical information including histology, debulking status, stage, grade, age, primary/metastasis, progression-free survival and overall survival for each sample of the datasets in Suppl. Tables 15A and 15B.

2. The PA-1 cell line representing "Stem-A" was derived from human ovarian teratocarcinoma cells, which is not EOC but a germ cell tumor (Zeuthen et al. Int J Cancer 1980).

> We agree with the referee that the PA-1 cell line was established from human ovarian teratocarcinoma not from epithelial ovarian carcinoma. However, it is also clear that PA-1 shares a common transcriptional signature with Stem-A EOCs; in particular, serous carcinoma. Our classification of the cell line panel is purely based on the expression profile, without considering the histological origin of the cell line.
>
> Further, we noted that the 1,538 tumors included only one teratocarcinoma sample (in GSE2109) that was classified into the Stem-A subtype; this implies common molecular profiles between teratocarcinoma and Stem-A EOC *in vivo*. Our findings thus suggest that the gene expression profiles can classify molecular phenotypes beyond what is apparent from histological origin.
>
> (Please note that this is further addressed on Page 11 of this rebuttal in response to comment 9, part II.)

Referee #1 (Specific Remarks):

This study presents a large meta-analysis of epithelial ovarian cancer (EOC) microarray datasets in which five molecular subtypes with different characteristics are identified. A collection of 16 microarray datasets was used as a training set, and combined with a collection of 5 microarray datasets for validation. The successful clustering of multiple data sets is remarkable as a batch effects are common and approaches such as ComBat tend to remove not only batch effects, but also biological variation.

> We agree with this Referee that biological variations might be affected when attempting to remove technical variation. ComBat was used in the current analysis, since it showed the best precision, accuracy and overall performance among various algorithms to effectively adjust for batch effects while conserving meaningful variations (Chen *et al*, 2011). However, the biological variation of the assembled data in the current study is apparent even in the post-ComBat data, where we can see different molecular subgroups with distinct clinical characteristics exemplified by survival outcomes and age. This finding indicates that biological variations of interest were conserved, as shown in Suppl. Figs. 1A and 1B. We now included this description in the section "Molecular heterogeneity of epithelial ovarian cancer" of the "Results".

Finally, a genome-wide shRNA screening method is utilized to identify subtype-related growth-promoting genes in selected molecular subtypes, and validated five genes as "Stem-A" related growth-promoting genes using PA1 cell line. A pathway analysis to suggest an association between mitochondrial metabolic pathways and "Stem-A" cell proliferation.

Generally, the manuscript should be more realistic about the results and be put more clearly in the context of previous findings; there is a general tendency for overstating the findings that aims to convince the reader of the importance of the study. This is unnecessary. Throughout the Results section the logic of why certain choices were made or how to interpret certain results needs to be more clear. A lot of insider's knowledge is expected from the reader.

We considered both Referees' comments and carefully re-wrote the relevant parts in the "Introduction", "Results" and "Discussion". First, we restated our findings more precisely, removing instances where we had overstated the results as much as we noted throughout the manuscript. Second, we made a significant effort to clarify the logic in the design and interpretation of the experiments to render the manuscript understandable for non-experts. Finally, we added to the Supplemental Information more detailed descriptions of the bioinformatics approaches for expression microarray analyses and experimental methodologies for shRNA/siRNA screens.

The manuscript represents a large body of work with a potentially exciting end result - possible involvement of energy metabolism in a specific subset of EOC - but it leaves the reader with an a bit of an empty feeling as the end result is entirely speculative. The study would greatly benefit from an attempt to show that energy metabolism is what sets the StemA subset apart from the other subtypes. Without follow-up to this result, it remains unclear what the true contribution of this study is, specifically of the shRNA/siRNA screens.

As suggested by both Referees, we have included follow-up validation studies of our shRNA/siRNA screen.

Initially, we observed that genes involved in mitochondrial oxidative phosphorylation were transcriptionally more active in Stem-A cell lines than in cell lines of the other molecular subtypes. Selective down-regulation of oxidative phosphorylation-related genes was further observed in PA-1 with si*TUBGCP4*, *siNAT10* or si*GTF3C1*. Based on these observations, our initial hypothesis was that cells of the Stem-A subtype might rely on mitochondrial oxidative phosphorylation as a source of energy more than those of the other subtypes.

During the course of the revision, however, we noted that transcriptional elevation of the genes for mitochondrial oxidative phosphorylation did not coincide with an actual activation in the oxygen consumption rate measured with the XF24 extracellular flux analyzer (Seahorse Bioscience, Billerica, MA) in cultured cells (image analysis based on the changes in carbonylcyanide-4- (trifluoromethoxy) -phenylhydrazone (FCCP)-uncoupled respiration). Moreover, suppression of mitochondrial membrane gene expression by siRNA treatment against *TUBGCP4* or *NAT10* was accompanied by the activation of apoptosis, as measured by cleavage of Caspase 3 and PARP in PA-1 cells (Suppl. Fig. 13C). It is, therefore, more reasonable to conclude that suppression of the mitochondrial membrane gene expression implies an apoptotic state of the cell rather than decreased activity of mitochondrial metabolism (as suggested by Referee #3).

Instead, we now find a significant increase in the expression of gene sets for microtubule-associated complexes or -related processes in Stem-A clinical samples and cell lines (Fig. 4A; Suppl. Table 16). Treatment with si*TUBGCP4*, a critical gene for tubulin nucleation, was also found to coincide with the down-regulation of microtubule-related genes (Suppl. Fig. 13B). Importantly, *in vitro* studies using drugs that interfere with microtubule polymerization revealed that the GI50 for vincristine and vinorelbine of the Stem-A cell lines was significantly lower than that for non-Stem-A cell lines (Fig. 4B). Such a difference was not observed for paclitaxel (drug that stabilize microtubule), the current standard of care used in the treatment of ovarian cancer (Fig. 4B). These observations imply that drugs interfering

with the tubulin polymerization pathway could be considered to selectively treat Stem-A-type ovarian carcinoma. In the revised version of the manuscript, we therefore focus on describing this identification of the connection between Stem-A and tubulin formation. We modified the “Abstract”, “Results”, “Discussion” and “Materials and Methods” accordingly, and also added Suppl. Table 16.

Detailed comments are described below.

1. "Computation of entropy showed that 1,500 or more samples were required to capture the complexity and dynamicity of EOC (Suppl. Fig. 1B) (Ewens & Grant, 2001)." Fig 1B shows that about 500 samples is likely to capture the major entropy.

We used the entropy plot to show that the number of samples employed for the analysis (n = 1,538 in total) was necessary and sufficient to provide a robust molecular classification in ovarian carcinoma. However, to address the comments from both Referees, we utilized a statistical power analysis to estimate the minimum sample size (Suppl. Fig. 2; Suppl. Materials and Methods).

We sought to distinguish a given $\mu_{Subtype\text{-}X}$ from $\mu_{Non\text{-}Subtype\text{-}X}$, using a two-sided *t*-test with significance set at $\alpha = 0.05$. Based on these parameters, we generated a power plot for each subtype. The sample number required for segregation was then computed as Epi-A = 1,298, Epi-B = 1,119, Mes = 785, Stem-A = 620, and Stem-B = 1,665; these numbers would achieve a desirable power of greater than 0.8, which is a conventionally accepted level of statistical power (Fox & Mathers, 1997). This result implies that our sample number of 1,538 is indeed appropriate for the molecular subtyping of ovarian carcinoma. We presented the results of the statistical power analysis as Suppl. Fig. 2, and described this in the section “Molecular heterogeneity of epithelial ovarian cancer” of the “Results” and in the “Suppl. Materials and Methods”.

2. As was reported by TCGA, serous ovarian carcinoma can be separated into four subgroups that were very similar to the serous ovarian carcinoma subgroups of Tothill et al. See also the review by David Bowtell in Nat Rev Cancer, 2010. These results should be acknowledged in the introduction. Supplemental Table 3 shows that the clusters reported here are again very similar to Tothill's. This similarity is recognized and the authors need to be careful not to overstate the novelty of the subgroups reported throughout the manuscript. For consistency, the authors should compare the names they propose for their subtypes to those proposed by Tothill (c1-c6) or TCGA (differentiated/c4, mesenchymal/c1, immunoreactive/c2, proliferative/c5); they should strongly consider to use these previously proposed naming conventions for consistency.

Although we originally acknowledged the relevance of the two previous studies by Tothill *et al.* (Tothill *et al*, 2008) and by TCGA (The Cancer Genome Atlas Research Network, 2011) in the second paragraph of the Introduction, it is now clear that our original description lacked clarity in describing the relationships between the different molecular subtyping methods. The classifications made by TCGA and the present study were compared with that of Tothill *et al.* (Tothill *et al*, 2008) (Suppl. Table 3). We now provide a scheme of the mutual relationships between the three molecular subtyping methods in Suppl. Fig. 3B. This is also described in the section of “Molecular heterogeneity of epithelial ovarian cancer” in the “Results” section and in “Suppl. Text” of the revised manuscript. It is important to note that the subtypes described by Tothill *et al.* or by TCGA (The Cancer Genome Atlas Research Network, 2011) do not show a one-to-one match with the subtypes established in our study (see the relationship among C2, C3 or C4/Immunoreactive or Differentiated/Epi-B or Epi-A). These differences in subtype classification may suggest a shared biological feature across these subgroups and hence may cause an imperfect distinction of the subtypes using predictive models. We also noted that the TCGA molecular subtyping lacks the Stem-B/C6

population, which is described in more detail in the response to the Major Comment #3 of Referee #1.

To better describe the connection between our study and these two prior studies, we now describe our subtypes as follows: Epi-A/C3/Differentiated; Epi-B/C2/Immunoreactive or Epi-B/C4/Differentiated; Mes/C1/Mesenchymal; Stem-A/C5/Proliferative, and Stem-B/C6 when these terms first appear in the revised manuscript. However, because of the discrepancy among the subtyping schemes, we use our own terminology in further instances.

3. A breakdown of the subgroups by data set as well as by histology should be provided to allow full interpretation of the clustering results. The authors use not only high-grade serous types but also other histologic types that share few molecular similarities (Zorn et al. Clin Cancer Res 2005; Vaugen et al. Nat Rev Cancer 2011). Especially, most invasive mucinous ovarian cancers are metastases from the gastrointestinal tract to the ovary. Therefore, the authors have to be especially careful about the interpretation of the results when molecular subtypes are identified in EOC. Although the authors showed clinical information in Supplementary Fig. 2, the details of clinical information per subtype are unknown. More details of clinical information per subtype in the training and validation datasets should be provided as a Table.

In response to this comment, the breakdown of subtypes by dataset and by histology has been added in Suppl. Figs. 4A and 4B.

TCGA subdivided high-grade serous cancers into four molecular subgroups, which lacked an identifiable Stem-B/C6 population, even when using all available histotypes (in the current study), or just the serous, endometrioid and serous LMP histologies (Tothill *et al*, 2008). On the other hand, in our analyses, many of the histotypes other than serous cancers were classified into the Stem-B subtype (Suppl. Figs. 4B and 6A), although these non-serous tumors shared “few molecular similarities”. Inclusion of the other histotypes, therefore, may allow the identification of a unique subgroup in serous carcinomas. To see the effect of including the other histologies in our molecular classification (using a standard deviation cut-off of 1.05), we examined the lists of genes that were most variably expressed across the samples as below.

1) All samples used in the current study (n = 1,538)
2) Serous carcinoma samples (n = 1,274)
3) Samples with histotypes other than serous carcinoma (n = 264)

These lists showed a significantly higher overlap between all 1,538 and 1,274 serous samples (1,116 overlapped/1,138 genes in total; 98.1%), than between all 1,538 and 264 of the other histology samples (1,089 overlapped/1,311 genes in total; 83.1%). This difference in the gene lists indicates that the overall expression pattern with all 1,538 samples was influenced mainly by variation within the serous carcinoma samples but to a lesser extent than that within the other histological types. The other histotypes might have been thus classified into the same molecular category as Stem-B, even though they exhibited distinct biological characteristics. However, it is also likely that the variation between serous and the other histologies contributed to the identification of the Stem-B subtype exhibiting less serous features. In fact, the Stem-B/C6 subtype was identified from the analyses of multi-histotype ovarian cancers by Tothill *et al.* and by us, but not in the analysis of only high-grade serous ovarian cancer by TCGA, as mentioned previously. This notion was also supported by the fact that the expression level of *WT1* gene, a marker of serous adenocarcinoma of the ovary (Lawrenson & Gayther, 2009) was significantly lower in Stem-B tumors, irrespective of whether they were exhibiting other histotypes or were only serous ovarian carcinoma (Suppl. Fig. 5). We now include this description in “Suppl. Text”.

More detailed clinical information regarding the molecular subtype in the training and validation datasets are provided in Suppl. Tables 4A, 4B, 4C and 4D. Moreover, clinical information for each sample in the training and validation datasets is given in the new Suppl. Tables 15A and 15B.

4. It should be briefly explained what the BinReg tool is and does. 78.8% classification accuracy of this approach seems low and unconvincing and it surprising that the core samples perform worse (78.5%) than non-core samples.

More details of the BinReg method have been added to the "Materials and Methods" in the section "Predictive modeling and validation by BinReg". We now also provide the URL links to the detailed descriptions by the original authors and to the software.

In spite of recent progress in the development of statistical models for molecular subtyping with expression microarray data, there is still uncertainty about the reproducibility of the various classification algorithms because of the intrinsic nature of subtype identification where the true classification remains unknown (Haibe-Kains *et al*, 2012). The concordance between pairs of different classifiers varied from 49-86% (median: 68%) in the molecular subtyping of 5,715 breast cancer samples derived from 36 independent datasets (Haibe-Kains *et al*, 2012). Similarly, a concordance of 77.5% was observed between a centroid prediction and a *k*-means clustering in a breast cancer cohort with 412 samples (Calza *et al*, 2006), showing that the best methods achieve a concordance comparable to the 78.8% we observed in our study.

By scrutinizing the discrepancy in the subtype classification, we noted high rates of discordant assignments in distinguishing Epi-A from Epi-B or Stem-B, and Epi-B from Mes (Suppl. Table 8). This ambiguity may arise from shared biological properties between some of the subtypes; for example, all three Epi-A, Epi-B and Stem-B subtypes expressed epithelial markers, and many clinical samples with Epi-B or Mes subtype assignment expressed inflammatory cell markers (Fig. 1A; Suppl. Fig. 11A; Suppl. Text). A similar overlap was indeed observed in a breast cancer cohort between luminal A and luminal B subtypes, which shared a nuclear expression of estrogen receptors (Calza *et al*, 2006). In fact, 82% of 489 ovarian cancer expression data of TCGA were assigned to more than one subtypes in a cross-validation with ss-GSEA (Verhaak *et al*, 2013), implying there were transcriptionally overlapped features across the samples. We believe that the observed discordance is due to similarity in the biological properties of the clinical samples. We now include this description in the "Suppl. Text".

To define core samples in the analysis, we used gene sets that are the most variably expressed across the 1,538 samples, while the gene sets for BinReg were selected to distinguish a given subtype from the others in a supervised manner. The gene components for each gene set were largely distinct, with a 9.8% overlap (116/1185 genes). Moreover, the silhouette width for the genes with variable expression was not correlated with that for the BinReg subtype signatures (Spearman correlation $rho = 0.0194$ and $p = 0.4612$). These findings are not inconsistent with our observation, in which the core samples were not always predicted as anticipated.

It should be explained why the results of combined clustering of validation data set and training data set to find the subtype of samples in the validation data set, was used over the more conventional approach of using a classifier (in this case ClanC).

In order to assess the accuracy of a predictor generated by BinReg or ClaNC, the "true" subtype must be known for a validation sample. In the original manuscript, as a means of assigning a "true" subtype to the validation samples, we employed the combined clustering

approach, in which the validation and training datasets were combined and subsequently clustered. Independently, BinReg or ClaNC was used to predict a subtype status of a validation sample purely based on the expression data of the training dataset. We then compared the subtype derived from the combined clustering with that from BinReg or ClaNC.

Nevertheless, to avoid confusion, we removed the combined clustering part and hence the assessment of accuracy/concordance of the validation samples in the revised manuscript. We instead present only the prediction results from BinReg and ClaNC (Suppl. Figs. 7D and 8D). To compare the similarity of the subtypes identified between the training and validation sets, we used clinicopathological features as well as the Spearman correlation analysis in the expression, and show the results in Fig. 1E, Suppl. Figs. 7D and 8D, and Suppl. Tables 4A, 4C and 4D. We now provide a detailed description in the "Predictive framework for EOC subtype classification" section of the "Results" in the revised manuscript.

Similarly, why were cell lines not classified (using ClanC or another classification method) but clustered.

In the original manuscript, we did not describe the reason why we employed the co-clustering method other than predictive models in classification of the panel of cell lines. First, co-clustering is a conventional method to classify cell lines into molecular subgroups with expression microarray data (Lowe *et al*, 2007; Perou *et al*, 1999; Prat *et al*, 2010; Virtanen *et al*, 2002). Second, we in fact performed cell line classification based on the predictive models by ClaNC and BinReg, relying on subtype signatures from clinical samples. The results were then compared with that obtained by the co-clustering method. Taking advantage of 28 biological replicate cell lines (Duke, Kyoto, and Singapore; see also "Cell line panel" in "Suppl. Materials and Methods"), we examined whether subtype assignment was consistent across the replicates. While consistency was 100% (28/28 cell lines) in the assignment by the co-clustering, both ClaNC and BinReg resulted in a less consistent subtyping (BinReg: 67.9%; 19/28 cell lines, ClaNC: 67.9%; 19/28 cell lines, Suppl. Table 9). Based on this observation, we decided to employ the co-clustering method instead of the predictive models. To make this clearer, we modified the description in the section of "Identification of representative cell lines for each subtype" in the "Results", added a more detailed description in the figure legend for Suppl. Fig. 10A, in the "Suppl. Text", and included Suppl. Table 9 in the revised manuscript to explain why we employed co-clustering over the other methods. In addition to Figs. 2B and 2C, transcriptional similarity of a given subtype between the cell lines and the clinical samples was further confirmed with a Spearman correlation map analysis and the result obtained was added as Suppl. Fig. 10C.

It is surprising to find cell lines of the class that resembles Tothill's c2, as the c2 class was related to tumor associated T-lymphocytes that are not part of any cell line model.

We agree with the Referee that tumor-associated inflammatory cells, including T-lymphocytes, are not part of any cell line model. However, it is also true that Epi-B cell lines share transcriptional profiles with the Epi-B clinical tumor samples. The similarity in the expression was confirmed with the BinReg analyses at gene and pathway levels in the original manuscript (Figs. 2B and 2C) and with the newly added Spearman correlation map in the revised manuscript (Suppl. Fig. 10C). It is plausible that the similarity between cell lines and clinical samples is intrinsic to carcinoma cells and not to contaminating inflammatory cells, as supported by several lines of evidence: 1) The Epi-B subtype of clinical samples consists of Tothill's C2 and C4, as illustrated in Suppl. Fig. 3B, as well as in the response to the comment #2. Tothill's C4 is a tumor cluster with fewer infiltrating cells than C2 (Tothill *et al*, 2008); therefore, the Epi-B subtype in clinical samples is not solely characterized by inflammatory cell infiltration (Fig. 1A). 2) Epi-B tumors are characterized not only by the marker gene

expression of inflammatory cells, such as major histocompatibility complex (MHC) class II genes (*HLA-DMs*, *DOs*, *DPs*, *DQs* and *DRs*), but also by gene sets for epithelial cells and interferon (IFN) pathways, such as IFN-downstream genes (Fig. 1A; Suppl. Figs. 11A and 11B). It is important to note that Epi-B cell lines also retain a similar expression pattern in these subsets of genes that include even MHC class II genes (Suppl. Figs. 11A and 11B). This ectopic expression of MHC class II genes in Epi-B cell lines may be induced through the IFN signaling pathway, since intrinsic inflammatory pathways in cancer cells (such as TNF-α and IFNs) can be activated by tumorigenic events. Also, IFNs are reported to stimulate the expression of MHC genes in various cancer cells including ovarian cancer *in vitro* and *in vivo* (Boyer *et al*, 1989; Freedman *et al*, 2000; Mantovani *et al*, 2008). We have now included these descriptions in the "Suppl. Text".

5. An association between molecular subtypes and prognosis is observed and referred to in the discussion as well as the results. This result should be discussed in the context of covariates such as surgical status (or status of residual tissue), which is one of most important prognostic factor in EOC (deBois et al. Cancer 2009). Progression-free survival time (or time to recurrence) should be included.

We agree with the Referee that multiple parameters or status should be examined to robustly identify prognostic factors. Table 1 reports the analysis of the different histological ovarian cancer types with age, stage, grade, metastasis and molecular subtype status for overall survival rate. In response to this comment, we further performed four additional Cox hazardous regression analyses described below (Suppl. Table 5A).

1) Serous cancer cases with age, stage, grade, metastasis and molecular subtype status based on the overall survival rate (OS) (Suppl. Table 5B)
   : The Stem-A or Epi-B subtype was found to be an independent prognostic factor from multiple clinical characteristics for serous ovarian cancer patients (Suppl. Table 5B).

2) Serous cancer cases with age, stage, grade, metastasis status and molecular subtype status based on the progression-free survival rate (PFS) (Suppl. Table 5E).
   : The molecular subtype was not independently correlated with PFS for serous ovarian cancer (Suppl. Table 5E).

3) All histology cancer cases with age, stage, grade, metastasis status, surgical status and molecular subtype status based on OS (Suppl. Table 5C).
   : The limited number of cases with complete information prevented us from identifying even the debulking status as an independent prognostic factor with statistical significance. Similarly, none of the molecular subtypes was found as an independent prognostic factor (Suppl. Table 5C).

4) All histology cancer cases with status for debulking surgery or molecular subtypes based on OS (Suppl. Table 5D).
   : When we examined only the debulking surgery status and the molecular subtype, Epi-B and Stem-A were found to be significant prognostic factors independent of the debulking status (Suppl. Table 5D).

In summary, the Stem-A and Epi-B subtypes are consistently identified as prognostic factors that are independent of multiple clinical parameters and status, which include the status for surgery in the overall survival rate (Table 1; Suppl. Tables 5A, 5B, and 5D). We now include these findings in the section of "Correlation of subtype with clinicopathological parameters" in "Results", and provide detailed descriptions in the "Suppl. Text".

The Kaplan-Meier survival curves (Fig. 1B and Suppl. Fig. 3D) of training and validation sets suggest a difference in prognosis of "Stem-B" subtype, which includes many samples with non-serous histologic types. This suggests that the outcome differences observed are due to the inclusion of different histologies.

The Kaplan-Meier curves for the Stem-B subtype differed between the training and validation sets, while those curves for the other subtypes were consistent (Fig. 1B; Suppl. Figs. 7C, 7D, 8B and 8D). The small number of samples for Stem-B [n = 56 (BinReg) or 32 (ClaNC) samples with survival information] in the validation dataset might be responsible for this discrepancy. However, we also found that the serous samples included in the Stem-B subtype have different overall survival rates between the training and validation datasets (data not shown), implying possible heterogeneity within the subtype. We described this finding in the "Suppl. Text".

6. It is suggested that the EOC cell lines mimic the clinical outcome behavior of the primary tumor samples. Does this result hold up when only including serous tumors into the analysis, or are the differences observes due to different histologies and the inclusion of LMP cell lines.

To address this comment, co-consensus clustering of the cell lines and the serous tumor samples was performed, and yielded a result that was highly concordant (137/142, 96.5%) with that of the original analysis. We noted that the five discordant arrays included three biological replicates of TYK-nu cell line and one of its derivatives (TYK-nu CisR) (data not shown), which were originally grouped into Mes subtype but were clustered with Stem-A serous tumors when clustering with serous tumors only. Nevertheless, this highly concordant assignment demonstrates the robustness of the co-clustering method and implies a very modest influence of the other histologies when included with serous tumor samples. We described this observation in the "Suppl. Text".

The cell-line panel used in the study comprises a mix of histologies according to the publicly available descriptions (Suppl. Table 11). To examine the influence of the original histology on the cell-line phenotyping, we selected the cell lines derived from serous carcinoma and correlated their *in vitro* properties with the molecular subtype. Although the differences were reduced due to the limited number of the cell lines, a similar trend could still be observed with statistical significance, being exemplified by the fact that Epi-A and Epi-B cell lines retained lower colony forming abilities and a longer population doubling time (Fig. R1). The Referee also suggested that we examine the possible effect by inclusion of LMP cell lines into the analysis. However, we could not perform the experiment since our panel did not contain LMP cell lines (Suppl. Table 11).

Figure R1: Cell-line phenotypes for each subtype with serous origin (*p*-value is computed by Mann-Whitney test)

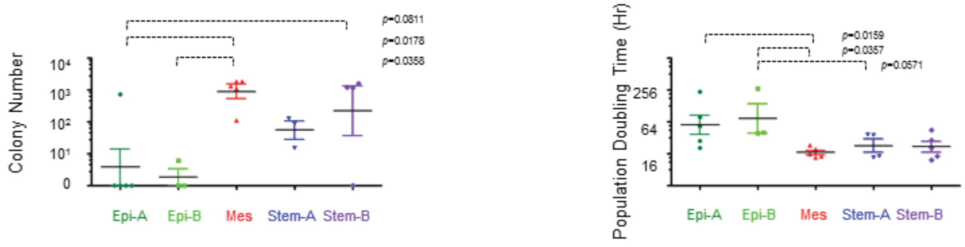

7. How were the 14 cell lines used for shRNA screen selected? What was the histology of the cell lines? The term 'shRNA copy number' was confusing as copy number is often used to reflect DNA

dosage; similar for the terms deleted/amplified. What does it mean when an shRNA is depleted/amplified? Is the target gene than suggested to be a growth inhibitor/stimulator? Not all readers will understand these details.

The 14 cell lines were chosen based on the silhouette width for the subtype signature in order to use "more representative" cell lines for a given subtype. We added a description of this in the section of "Genome-wide shRNA screens identified subtype-specific growth-promoting genes" in the "Results". The silhouette width of all cell lines was included in Suppl. Table 11.

We provided more detailed descriptions of the shRNA screening in "Introduction", "Results" as well as "Materials and Methods" to clarify the terminology, the experimental procedures and the interpretations for the results obtained.

8. There should be more details on the implementation of the shRNA screen in the main text. For instance, details on how the 'shRNA copy number' is assessed on compared between different shRNAs and cell lines would be helpful in interpreting the result. How many genes are targeted by the library and are the numbers of identified shRNAs/genes more than expected by random chance?

We appreciate comments #7 and #8 for pointing weaknesses in our description of the shRNA experiments. As mentioned in the response to comment #7, we now provide detailed explanations for the shRNA screens particularly regarding the terminology, library, experimental system, statistical method and interpretations in the "Introduction", "Results" and "Materials and Methods".

The responses below answer the two specific questions:

1) The TRC library used in this study contains 80,000 lentivirally expressing shRNAs, corresponding to 16,000 human genes.

2) The subtype-specific essential genes were computed using RIGER software (Luo *et al*, 2008), in which the *q*-values are computed by the Kolmogorov-Smirnov test based on 1,000 permutations and subsequently adjusted by the Benjamini and Hochberg procedure (http://www.broadinstitute.org/cancer/software/GENE-E/; Suppl. Table 12). The *q*-value (false discovery rate) for each gene indicates the probability of that particular gene being significantly amplified (increased copy number due to increased number of cells carrying this shRNA) or depleted as compared to what would occur by mere random chance. Using the threshold with a stringent *q*-value (for example, $q < 0.005$), shRNAs targeting 409 genes were then identified as significantly amplified or depleted more than that which would be expected by random chance (Suppl. Table 12). To avoid confusion, we changed the name of "*p*-value" column to "false discovery rate *q*-value" in Suppl. Table 12 and provided more information about RIGER analysis in the "Materials and Methods" of the revised manuscript. In addition, we provide the link to the RIGER software in "For more information".

How many genes were deleted/amplified in all cell lines? Although the subtypes are an interesting characteristic of EOC, it is not clear why those genes that are different between subtypes would be the most disease-relevant, rather than genes associated with all cell lines.

We do agree with the Referee that it is important to identify "EOC common" molecular targets and that the shRNA screening approach can be also utilized for this purpose. In fact, Cheung *et al.* performed large screens and identified *PAX8* as a relevant molecule for the growth of ovarian cancer in a lineage-specific manner (Cheung *et al*, 2011).

However, there is a technical reason that hampers our identification of “depleted” or “amplified” shRNA in all cell lines; that is, “depletion” or “amplification” of shRNAs was defined by relative abundance across the cell lines in our experimental system. So, obtaining the EOC disease-relevant genes would require performing the assays using cell lines derived from tissues other than the ovary. The study by Cheung *et al.,* based on a different experimental system, relied on a comparison between the screens of 25 ovarian cancer cell lines with those of 77 cancer cell lines derived from the other tissues.

It is also important to point out that the main focus of the present study was to identify molecular targets for a subtype; therefore, EOC disease-relevant genes are beyond the scope of this study. The relevance of such subtype-specific targets has been exemplified by *ESR1* (estrogen receptor α) for luminal-subtype breast cancers. *ESR1* has been used not only for diagnosis but also as a molecular target for therapeutics. In the same vein, we hope that our study could contribute to the diagnostics and therapeutics associated with EOC through the identification of a potential molecular target for the Stem-A subtype. We include these remarks in the “Introduction” and “Discussion” of the revised manuscript to emphasize the ultimate goal of the shRNA screen.

Figure 3B shows a heatmap of the identified shRNAs in each of the celllines and the data is likely (z-score) normalized. The effects of this normalization should be explained, as the normalization will force the data to show (visually attractive) differences.

For the computation of differentially detected shRNA, the data was not z-score normalized. Using reads with a perfect match to the reference sequences (Sigma Aldrich, St Louis, MA), copy number was counted and normalized by the total number of reads in a sample and then rendered to RIGER analysis to find phenotype-specific, functionally relevant genes (Luo *et al*, 2008). As the Referee noted, the heatmap was plotted using gene normalization and centering, which is commonly performed to facilitate reading of the differences. Although we described this analytical method in the section of “Statistical identification of the functionally relevant genes in the subtype-specific manner” of “Materials and Methods” in the original manuscript, the description has now also been added to the legend of Fig. 3B in the revised manuscript.

9. Why is the number of genes depleted in Stem-A (n=135) different from what is reported in the previous paragraph (n=88)?

The disparity in the numbers of genes depleted in Stem-A in both paragraphs is due to the differences in the significance thresholds that we applied in the RIGER analysis. The initial number of 88 genes was obtained from the threshold at $q = 0.005$ and used for the heatmap presentation (Fig. 3B). A q-value of 0.03 was used as the threshold for the second part of the analysis, arriving at 135 genes. We provide more details in the section of “Validation of subtype-specific growth promoting genes” in the “Results” to clarify the differences in the number of genes identified by the shRNA screen.

The PA-1 cell line representing "Stem-A" was derived from human ovarian teratocarcinoma cells, which is not EOC but a germ cell tumor (Zeuthen et al. Int J Cancer 1980). This is likely to have an impact on the results and suggests that the cell line classification will be able to classify any expression profile and is not EOC specific.

This concern was already addressed on page 2 of this rebuttal in response to comment 2. Our responses are repeated here.

We agree with the Referee that the PA-1 cell line was established from human ovarian teratocarcinoma not from epithelial ovarian carcinoma. However, it is also clear that PA-1 shares a common transcriptional signature with Stem-A EOCs; in particular, serous carcinoma. Our classification of the cell line panel is purely based on the expression profile, without considering the histological origin of the cell line.

Further, we noted that the 1,538 samples included only one teratocarcinoma sample (in GSE2109) that was classified into the Stem-A subtype; this implies common molecular profiles between teratocarcinoma and Stem-A EOC *in vivo*. Our findings thus suggest that the molecular classification might not be specific to EOCs as the Referee pointed out.

Nevertheless, it is true that the different cell-of-origin could have an impact on the results. To exclude this possible impact, we performed additional validation siRNA experiments using three more Stem-A cell lines (CH1, A2780 and OVCAR-3) with five more non-Stem-A cell lines (Epi-A: OVCA429 and PEO1, Mes: ovary1847, SKOV-3 and HEY), for which the information of the origin is presented in Suppl. Table 11. We confirmed the reproducibility of the effect of si*TUBGCP4* and si*NAT10* on the set of cell lines, demonstrating that our finding is not limited to PA-1. We present the new results as Fig. 3E in the revised manuscript and provide a detailed description in the "Validation of subtype-specific growth promoting genes" section in the "Results" section.

10. The logic and flow of the "Validation of subtype-specific growth promoting genes" section is unclear. Was the HEYA8 cellline used a control in the t-test comparison? The identification of five genes as growth-promoting seems adequate, but is hard to interpret without better understanding what the control was.

We appreciate this comment and have now carefully edited the logic and flow of the validation process in "Validation of subtype-specific growth promoting genes" in the "Results" in the revised manuscript to facilitate a better understanding.

The primary aim of the validation was to identify the genes whose inhibition induced growth suppression in only Stem-A cells with a minimal effect on non-Stem-A cells. Stem-A-specific essential genes were identified as positive hits based on the following comparison using a Student *t*-test as well as based on ≥ 20% growth suppression of PA-1 cells (Fig. 3C):

1) Comparison between the negative siRNA controls with silencing the gene of interest in PA-1 cells.

2) Comparison between PA-1 with the reference cell line(s) in silencing the gene of interest.
   : The reference was HeyA8 at the 1st step, and HeyA8 and OVCA433 at the 2nd and 3rd steps during the process of validations.

The HeyA8 cell line was hence utilized as a comparison in the identification of Stem-A-specific genes for the 1st, 2nd and the 3rd steps during the process of validation (Fig. 3C). In view of comments earlier by the Referee, we also performed an additional 4th step of the validation process, using three more Stem-A cell lines (CH1, A2780 and OVCAR-3), with five more non-Stem-A cell lines as references (Epi-A: OVCA429 and PEO1, Mes: ovary1847, SKOV-3 and HEY). We provide these detailed descriptions in the section "Validation of subtype-specific growth promoting genes" of the "Results", and in the "Materials and Methods" to clarify the experimental procedures.

11. Were technical replicates performed for each cellline-shRNA expression array experiment? For any of such experiments, triplicates are adviced as a minimal number by any statistical model.

We indeed performed triplicate experiments for expression microarray with siRNA transfections. We had 58 gene expression arrays in total:
3 replicates of reverse transfections x (5 siRNAs + 1 negative control) x 3 cell lines
+ 4 replicates to bridge four microarray batches that correct the batch effect.

We provide detailed descriptions in the "Suppl. Materials and Methods" to clarify the experimental procedures in the revised manuscript.

Minor comments:

1. Paragraph 1: The authors should provide the eligibility criteria in this study including quality control of expression data.

In order for our study to have broader generalizations and larger sample size, reduced eligibility criteria were adopted (George, 1996). To clarify, we included the eligibility criteria and methods of quality control of the expression data in "Materials and Methods". Female adult (age ≥20) patients with a clinical diagnosis of primary or metastatic ovarian carcinoma were included in our analysis with no restriction on race, pre-treatment history or medical condition, stage, grade, or histology of the disease.

We checked the quality of Affymetrix-based expression profiling using Bioconductor AffyQCReport package (Gautier *et al*, 2004), and the criteria: average perfect-match (Neve *et al*) intensity, kernel density plot, *GAPDH* 3':5' ratio, β-actin 3':5' ratio, and center of intensity for positive and negative control. All chips passed at least one of the criteria, and hence, none of the samples was discarded.

2. Materials and Methods/Supplementary Table 11: The authors should describe source of information (for example GEO_ID) about the Duke Cohort dataset.

The GEO_ID of the Duke cohort was included in Suppl. Table 15A (Suppl. Table 11 in original manuscript).

3. Materials and Methods, paragraph 7: The authors should describe the name of cell lines precisely. Ex) Tyknu -> TYK-nu

The names of the cell lines were amended by following to the names in the original paper as well as to the names used by the distributors, such as ATCC and Riken.

4. Supplementary Table 1: The column of "Genomic data" is unnecessary because the authors didn't use genomic data except expression data. Instead, the authors should add a column of "chemotherapy".

The column of genomic data in Suppl. Table 1 was removed. However, due to a lack of information on the chemotherapy received by the patients, we did not include this column.

Referee #3:

The manuscript "Functional Genomics Identifies Five Distinct Molecular Subtypes with Clinical Relevance and Pathways for Growth Control in Epithelial Ovarian Cancer" by Tan, Huang and Miow et al. identified gene expression signatures associated with five subtypes of epithelial ovarian cancer (EOC). The authors analyzed the largest compilation of EOC expression profiles to date and defined molecular subgroups, which display unique clinical outcome. The authors extend their EOC molecular subgroup expression profiles to traditional in vitro cell lines, which allowed for their classification as clinically relevant models. Through the use of RNAi libraries, the authors identified genes that affect EOC sub-type specific tumor cell growth and suggest the biological pathways that may underlie these sub-type dependencies. The manuscript presents extensive data and analyses including an expression meta-analysis of a very large number of ovarian cancers, and genome-wide shRNA data for 14 ovarian cancer cell lines. However, some of the analyses could be improved, and many of the conclusions seem overstated.

> We thank the Referee for the comments. We have removed overstatements as much as we could identify throughout the manuscript. Concerns regarding the analyses are addressed one by one in the response to each comment as described below.

Major Concerns:

1) The subtyping analyses at times seem to be circular. For example, tumor assignments were determined for external datasets by applying BinReg using a predictive model generated from the internal data. These results were compared to "true" subtypes determined by pooling the external data with 1,142 samples from the internal data. In both cases, the internal data will dominate the analysis and determine similar cluster assignments.

> This has been addressed in our answer to Referee 1, question 4, part 2 on page 6. Our responses are repeated here.
>
> In order to assess accuracy/concordance of a predictor generated by BinReg or ClaNC, the “true” subtype must be known for a validation sample. In the original manuscript, as a means of assigning a “true” subtype to the external samples, we employed the combined clustering approach, in which the external and internal datasets were combined and subsequently clustered. Independently, BinReg or ClaNC was performed to predict a subtype status of an external sample purely based on the expression data of the internal dataset. We then compared the subtype derived from the combined clustering with that from BinReg or ClaNC.
>
> Nevertheless, to avoid confusion, we removed the combined clustering part and hence the assessment of accuracy/concordance of the external samples in the revised manuscript. We instead present only the prediction results from BinReg and ClaNC (Suppl. Figs. 7D and 8D). To compare the similarity of the subtypes identified between the internal and external sets, we used clinicopathological features as well as the Spearman correlation analysis in the expression, and show the results in Fig. 1E, Suppl. Figs. 7D and 8D, and Suppl. Table 4A, 4C and 4D. We now provide detailed description in “Predictive framework for EOC subtype classification” section of the “Results” in the revised manuscript.
>
> In the BinReg and ClaNC predictions, the internal data is used to generate a predictive model to assign any subtype to the external data. The prediction is based on inherent structural similarity in the expression between the external and internal data. It is important to note that the structure already exists within the external data and is not influenced by the internal data.

Likewise, the internal data were divided into deciles with leave-10%-out determination of the cluster assignments; these assignments were then compared to the assignments using all the data. Again, 90% of the data used in both analyses will dominate the cluster assignments, which will necessarily overlap as a result. A better approach would seem to be to divide the data into, for example, 40%, 40%, and 20%, then use each 40% independently to classify the 20%, and evaluate concordance.

We performed a 10-fold or leave-10%-out cross-validation since it has been reported and well adopted as a methodology to evaluate predictors (Blum A, 1999; Kim, 2009; Konavi, 1995).

Nevertheless, following the suggestion made by the Referee, we performed five random 40-40-20 hold-out tests so as to provide additional assessments for the ClaNC and BinReg predictors. Briefly, we randomly divided the data into sample sets made up of 40%, 40% and 20% of the total 1,538 samples, denoted as training set A, training set B, and testing set. To ensure a fair assessment, the division was such that each sample was used as a testing sample only once in the five random sets (Suppl. Materials and Methods). The results achieved with ClaNC or BinReg predictors are similar to that of 10-fold cross validation, with concordance of an average 73.6% (ClaNC) or 74.0% (BinReg) as shown in Suppl. Fig. 9 in the revised manuscript. This has been described in the "Predictive framework for EOC subtype classification" of the "Results" and detailed descriptions of the experimental procedure are included in the "Suppl. Materials and Methods".

Similarly, the tumor data were used to determine a classification scheme for the cell lines, and the resultant subtype assignments were compared to assignments obtained from clustering of all the tumors and cell lines together. Again, the concordance of the results simply reflects the fact that substantially the same data were used to determine the clustering pattern in both analyses.

We evaluated the cell-line subtype derived from co-clustering by comparing similarity of the subtype between the cell lines and clinical samples. Although we agree with the Referee that this process may look circular, it is important to point out that there are two distinct steps embedded in the process: first, identification of the cell-line subtype, and second, confirmation of the expression similarity of the subtype. For the first step, the co-clustering method was employed (Suppl. Fig. 10A); for the second step, the BinReg and Spearman analysis was performed (Figs. 2B and 2C; Suppl. Fig. 10C). Importantly, the BinReg or Spearman analysis do not largely rely on the same genes that were used for the co-clustering analysis (BinReg; 4.33% = 65 overlapped in total 1,502 genes, Spearman; 10.12% = 145 overlapped in total 1,433 genes). We include a description in the section "Cell line subtype identification by consensus clustering" in the "Suppl. Text" to make this point clearer.

2) I do not understand the justification or process for classifying cell lines based on two rounds of consensus clustering. It would seem that CLANC could just be applied based upon the original clustering results.

This has been addressed in our answer to Referee 1, on page 7. Our responses are repeated here.

In the original manuscript, we did not describe the reason why we employed the co-clustering method other than predictive models in classification of the panel of cell lines. First, co-clustering is a conventional method to classify cell lines into molecular subgroups with expression microarray data (Lowe *et al*, 2007; Perou *et al*, 1999; Prat *et al*, 2010; Virtanen *et al*, 2002). Second, we in fact performed cell line classification based on the predictive models by ClaNC and BinReg, relying on subtype signatures from clinical samples. The results were then compared with that obtained by the co-clustering method. Taking advantage of 28 biological replicate cell lines (Duke, Kyoto, and Singapore; see also "Cell line panel" in

"Suppl. Materials and Methods"), we examined whether subtype assignment was consistent across the replicates. While consistency was 100% (28/28 cell lines) in the assignment by the co-clustering, both ClaNC and BinReg resulted in a less consistent subtyping (BinReg: 67.9%; 19/28 cell lines, ClaNC: 67.9%; 19/28 cell lines; Suppl. Table 9). Based on this observation, we decided to employ the co-clustering method instead of the predictive models. To make this clearer, we modified the description in the section of "Identification of representative cell lines for each subtype" in the "Results", added a more detailed description in the figure legend for Suppl. Fig. 10A, in the "Suppl. Text" and included Suppl. Table 9 in the revised manuscript to explain why we employed co-clustering over the other methods. In addition to Figs. 2B and 2C, transcriptional similarity of a given subtype between the cell lines and the clinical samples was further confirmed with a Spearman correlation map analysis and the result obtained was added as Suppl. Fig. 10C.

3) On p. 13, "Importantly, our shRNA screens detected specificity in the shared pattern within a subtype in the depletion or amplification of shRNAs." Yet it is not clear that the depleted/amplified shRNAs were significant when p-values are corrected for multiple hypotheses. One straightforward way to validate the data (beyond the individual hairpins evaluated in single lines in the following experiments) would be to compare the results to previously published genome-level shRNA screens in ovarian cancers, eg Cheung et al, PNAS 2011.

The *q*-value outputted by RIGER is already adjusted by the Benjamini and Hochberg procedure (http://www.broadinstitute.org/cancer/software/GENE-E, RIGER section). We modified the column name '*p*-value' to 'false discovery rate *q*-value' in Suppl. Table 12 and provided more information on the RIGER analysis in the "Materials and Methods" of the revised manuscript. In addition, we provided the link to the RIGER software in "For more information".

We appreciate the Referee's suggestion to compare the results in the current study with that in the previously published shRNA screens. However, a comparison between Cheung *et al.* (2011) data and ours is not feasible because of the different experimental designs (at the endpoint of the screen, we compared the abundance of integrated shRNA sequences across the different molecular subtypes, while Cheung *et al*. (2011) compared the relative abundance to the initial shRNA pool across different cell line lineages). The different detection platforms (next-generation sequencing versus microarray) also prohibit this comparison. We, however, have now included this description in the "Suppl. Materials and Methods". It is important to note that we added further rigorous validations for *TUBGCP4* and *NAT10* with an increased number of cell lines (Figs. 3C and 3E).

Also, I would be interested to know the effect sizes of these hairpins (not just the ones explored in detail in following experiments). RIGER was applied using a KS statistic, which is valid but may highlight hairpins with small (though consistent) effect sizes.

As suggested by the Referee, we included the effect size (standardized copy number difference) of each hairpin in the Suppl. Table 12. The effect size is reasonably large as the effect size is ≥ 0.7 or ≤ -0.7 (Cohen, 1988; Monk *et al*, 2012; Syrjanen & Syrjanen, 2013); the mean effect sizes of depleted hairpins were Epi-A = -0.9098; Mes = -0.7681, and Stem-A = -0.7818, and those of amplified hairpins were: Epi-A = 0.8128, Mes = 0.8282, and Stem-A = 0.7486 (Suppl. Fig. 12B). We now included these descriptions in "Genome-wide shRNA screens identified subtype-specific growth-promoting genes" section of the "Results" in the revised manuscript.

4) The biological evaluation of the "Stem-A specific growth promoting genes" is limited in scope. In particular, the GSEA results implicating pathways are suspect because they represent comparisons of individual cell lines. Moreover, the authors conclude that finding "Mitochondria Membrane" etc as restricted to the response of the Stem-A line to shRNA to the 5 genes suggests a link between these pathways and cell proliferation. Rather, these pathways are likely to reflect effects of toxicity: namely activation of apoptotic pathways and cell death. Moreover, the Discussion paragraph on these pathways is very speculative. We need much more mechanistic data before we can implicate these pathways as therapeutic targets, and would have to at least try drugs targeting these pathways on the cell lines before proposing them for clinical trials.

As suggested by the two Referees, we included follow-up validation studies of our shRNA/siRNA screen. This has been addressed on page 3 in response to Referee 1 Specific comments. Our responses are repeated here.

Initially we observed that genes involved in mitochondrial oxidative phosphorylation were transcriptionally more active in Stem-A cell lines than in cell lines of the other molecular subtypes. Selective down-regulation of oxidative phosphorylation-related genes was further observed in PA-1 with si*TUBGCP4*, *siNAT10* or si*GTF3C1*. Based on these observations, our initial hypothesis was that cells of the Stem-A subtype might rely on mitochondrial oxidative phosphorylation as a source of energy more than those of the other subtypes.

During the course of the revision, however, we noted that transcriptional elevation of the genes for mitochondrial oxidative phosphorylation did not coincide with an actual activation in the oxygen consumption rate measured with the XF24 extracellular flux analyzer (Seahorse Bioscience, Billerica, MA) in cultured cells (image analysis based on the changes in carbonylcyanide-4- (trifluoromethoxy) -phenylhydrazone (FCCP)-uncoupled respiration). Moreover suppression of mitochondrial membrane gene expression by siRNA treatment against *TUBGCP4* or *NAT10* was accompanied by the activation of apoptosis, as measured by cleavage of Caspase 3 and PARP in PA-1 cells (Suppl. Fig. 13C). It is, therefore, more reasonable to conclude that suppression of the mitochondrial membrane gene expression implies an apoptotic state of the cell rather than decreased activity of mitochondrial metabolism (as suggested by the Referee in this question above).

Instead, we now find a significant increase in the expression of gene sets for microtubule-associated complexes or -related processes in Stem-A clinical samples and cell lines (Fig. 4A; Suppl. Table 16). Treatment with si*TUBGCP4*, a critical gene for tubulin nucleation, was also found to coincide with the down-regulation of microtubule-related genes (Suppl. Fig. 13B). Importantly, *in vitro* studies using drugs that interfere with microtubule polymerization revealed that the GI50 for vincristine and vinorelbine of the Stem-A cell lines was significantly lower than that for non-Stem-A cell lines (Fig. 4B). Such a difference was not observed for paclitaxel (drug that stabilize microtubule), the current standard of care used in the treatment of ovarian cancer (Fig. 4B). These observations imply that drugs interfering with the tubulin polymerization pathway could be considered to selectively treat Stem-A-type ovarian carcinoma. In the revised version of the manuscript, we therefore focus on describing this identification of the connection between Stem-A and tubulin formation. We modified the “Abstract”, “Results”, “Discussion” and “Materials and Methods” accordingly, and also added Suppl. Table 16.

5) The manuscript defines 5 molecular subtypes of EOC. How do these subtypes compare with the 4 transcriptional subtypes described by the TCGA? Does your 5th subgroup subdivide a TCGA subtype, or does it represent a novel subtype?

This question was already answered earlier on page 4. Our responses are repeated here. Although we originally acknowledged the relevance of the two previous studies by Tothill *et*

al. (Tothill *et al*, 2008) and by TCGA (The Cancer Genome Atlas Research Network, 2011) in the second paragraph of the Introduction, it is now clear that our original description lacked clarity in describing the relationships between the different molecular subtyping methods. The classification made by TCGA and the present study were compared with that of Tothill *et al.* (Tothill *et al*, 2008) (Suppl. Table 3). We now provide a scheme of the mutual relationships between the three molecular subtyping methods in Suppl. Figure 3B. This is also described in the section of "Molecular heterogeneity of epithelial ovarian cancer" in the "Results" section and in "Suppl. Text" of the revised manuscript. It is important to note that the subtypes described by Tothill *et al.* or by TCGA (The Cancer Genome Atlas Research Network, 2011) do not show a one-to-one match with the subtypes established in our study (see the relationship among C2, C3 or C4/Immunoreactive or Differentiated/Epi-B or Epi-A). These differences in subtype classification may suggest a shared biological feature across these subgroups and hence may cause an imperfect distinction of the subtypes using predictive models. We also noted that the TCGA molecular subtyping lacks the Stem-B/C6 population, which is described in more detail in the response to the Major Comment #3 of Referee #1.

To better describe the connection between our study and these two prior studies, we now describe our subtypes as follows: Epi-A/C3/Differentiated; Epi-B/C2/Immunoreactive or Epi-B/C4/Differentiated; Mes/C1/Mesenchymal; Stem-A/C5/Proliferative, and Stem-B/C6 when these terms first appear in the revised manuscript. However, because of the discrepancy among the subtyping schemes, we use our own terminology in further instances.

TCGA subdivided high-grade serous cancers into four molecular subgroups, which lacked a Stem-B/C6 population that could be identified with making use of all available histotypes (in the current study), or of serous, endometrioid and serous LMP histologies (Tothill *et al*, 2008). It is likely that the variation between serous and the other histologies contributed to the identification of the Stem-B subtype exhibiting a "less serous" feature. This notion was supported by the fact that the expression level of *WT1* gene, a marker of serous adenocarcinoma of the ovary (Lawrenson & Gayther, 2009), was significantly lower in Stem-B tumors not only with all of the ovarian cancer samples but also with serous ovarian cancers (Suppl. Fig. 5). Therefore, we believe that the Stem-B subtype represents a novel subtype in serous ovarian cancer.

6) The clinical utility of the molecular subtypes is emphasized, but it is not clear to me that this is justified by the modest magnitude of difference in outcomes between the subtypes.

We agree with the Referee that the magnitude is modest in Kaplan-Meier or Cox hazardous regression analyses of the molecular subtype. However, the multivariate Cox regression on the molecular subtype, with known prognostic factors for ovarian cancer (age, stage, grade, metastatic state and surgical status), indeed indicates that at least Epi-B and Stem-A subtypes are robust prognostic factors for patients with ovarian carcinoma. This is particularly the case for serous carcinoma, in that it is independent of the other clinical parameters and status (Table 1; Suppl. Table 5A). We now include these findings in "Correlation of subtype with clinicopathological parameters" in the "Results", and also provided detailed descriptions in the "Suppl. Text".

Moreover, in the Discussion the authors state "we believe our classification represents a foundation for further development, particularly since ovarian cell lines can be assigned to unique ovarian tumor subtypes." It is not surprising that cell expression profiles could be so assigned-a random subtyping scheme would also seem to divide the cell lines.

To ensure the cell-line subtype was not derived from any random assignment, we assessed the validity of cell-line subtypes with Silhouette analysis in the original manuscript (Fig. 2A). SigClust was additionally performed in the revised manuscript, since it allows us to establish the significance of clustering using Monte Carlo simulations and provides a *p*-value for the clustering operation between two sets of points (Fig. 2A) (Liu *et al*, 2008). Both analyses revealed that cell line subtyping was statistically significant; therefore, it did not originate by a random scheme. We now included the additional SigClust analysis in Fig. 2A and also in "Identification of representative cell lines for each subtype" of the "Results".

It would be more interesting if the subtypes predicted other characteristics of the cell lines that were similar to tumors, eg genetics. The authors mention anchorage-independent growth and growth rate, but these are not measured in tumors.

We agree with the Referee that each subtype may also be distinguished by specific genome alteration patterns shared with their counterpart clinical tumors. Since the transcriptome and the functional genetic screens were our main focus, such an Omics study is beyond the scope of the current study. Following the suggestion, however, we are currently assembling a database including genomic alterations, miRNA, and methylation patterns for the panel of the cell lines. We are hoping to obtain significant results in the coming months to be part of a follow-up study.

Minor Concerns:

1) On page 6, "Computation of entropy showed that 1,500 or more samples were required..." suggests that there is a clear cutoff at 1,500, but I see little change past 500.

This comment has already been addressed on page 4. Our responses are repeated here. We used the entropy plot to show that the number of samples employed for the analysis (n = 1,538 in total) was necessary and sufficient to provide a robust molecular classification in ovarian carcinoma. However, to address the comments from both Referees, we utilized a statistical power analysis to estimate the minimum sample size (Suppl. Fig. 2; Suppl. Materials and Methods).

We sought to distinguish a given $\mu_{Subtype\text{-}X}$ from $\mu_{Non\text{-}Subtype\text{-}X}$, using a two-sided *t*-test, with significance set at $\alpha = 0.05$. Based on these parameters, we generated a power plot for each subtype. The sample number required for segregation was then computed as Epi-A = 1,298, Epi-B = 1,119, Mes = 785, Stem-A = 620, and Stem-B = 1,665; these numbers would achieve a desirable power of greater than 0.8, which is a conventionally accepted level of statistical power (Fox & Mathers, 1997). This result implies that our sample number of 1,538 is indeed appropriate for the molecular subtyping of ovarian carcinoma. We presented the results of the statistical power analysis as Suppl. Fig. 2, and described this in the section "Molecular heterogeneity of epithelial ovarian cancer" of the "Results" and in the "Suppl. Materials and Methods".

2) On page 7, "non-serous EOC appears to have a distinct molecular profile"-the data do not convincingly support this. Both the non-serous and (particularly) serous tumors appear to reside in several clusters, and no cluster contains only non-serous tumors.

We agree with the Referee that non-serous tumors also reside in the other molecular subtypes, and that there are serous tumors in the Stem-B subtype. It is now stated that the majority of non-serous EOC were significantly found in Stem-B (Fisher Exact test, $p = 8.0 \times 10^{-59}$; Suppl. Figs. 4B and 6A; Suppl. Tables 4A and 4B), and might imply "less serous" features of the

Stem-B subtype, as mentioned in the response to comment #5 from Referee #3 and comment #3 of Referee #1. Although the distinct molecular profiles to some extent reflect the biological nature of the tumors, it does not necessarily suggest any mechanism. Therefore, we removed the sentence: "As such, non-serous EOC appears to have a distinct molecular profile, suggesting different carcinogenic or progression mechanisms." from the "Correlation of subtype with clinicopathological parameters" in the "Results" of the revised manuscript.

3) In Supp Fig 1, it would be useful to see a heatmap of all the data, arranged by dataset, to evaluate the effectiveness of ComBat.

To address this comment, we plotted the heatmap of all the data, arranged by dataset, before and after ComBat, as suggested by the Referee. The new figure is added as Suppl. Figure 1A.

4) Supp Fig 2 is small and difficult to read. The equations in the Entropy analysis are also illegible in my copy.

We enlarged the figures and text in Suppl. Fig. 2 (Suppl. Fig. 6 in the revised manuscript). As we replaced the Entropy plot with the Power plot for Suppl. Fig. 1B (Suppl. Fig. 2 in the revised manuscript), the equations for the Entropy analysis are no longer shown in the revised manuscript.

5) ss-GSEA of EOC samples resulted in numerous gene sets enriched for each subtype. On page 7 the authors state, "Mes tumors correlated with metastases and TGFB-related pathways". Yet the Mes tumors were enriched for 177 gene sets/pathways. Is it correct to connect Mes tumor metastatsis to a single gene set if there are a multitude of gene sets enriched?

There are ~28 unique Metastasis-related and ~17 TGFβ-related gene sets in the Molecular Signatures Database version 3. Out of these gene sets, three metastasis-related (hypergeometric probability density function, p = 0.0287) and four TGFβ-related (hypergeometric probability density function, $p = 7.1 \times 10^{-4}$) gene sets are enriched in the Mes subtype. We believe our description was correct; however, at the same time, many gene sets, such as the curated genes from complex expression data, are often difficult to interpret. We therefore did not include the p-value computed for the hypergeometric probability density function analyses in the revised manuscript.

References

Blum A KA, Langford J (1999) Beating the hold-out: bounds for K-fold and progressive cross-validation. Computational Learning Theory**:** 203-208

Boyer CM, Dawson DV, Neal SE, Winchell LF, Leslie DS, Ring D, Bast RC, Jr. (1989) Differential induction by interferons of major histocompatibility complex-encoded and non-major histocompatibility complex-encoded antigens in human breast and ovarian carcinoma cell lines. Cancer research 49**:** 2928-2934

Calza S, Hall P, Auer G, Bjohle J, Klaar S, Kronenwett U, Liu ET, Miller L, Ploner A, Smeds J et al (2006) Intrinsic molecular signature of breast cancer in a population-based cohort of 412 patients. Breast cancer research : BCR 8**:** R34

Chen C, Grennan K, Badner J, Zhang D, Gershon E, Jin L, Liu C (2011) Removing batch effects in analysis of expression microarray data: an evaluation of six batch adjustment methods. PloS one 6**:** e17238

Cheung HW, Cowley GS, Weir BA, Boehm JS, Rusin S, Scott JA, East A, Ali LD, Lizotte PH, Wong TC et al (2011) Systematic investigation of genetic vulnerabilities across cancer cell lines reveals lineage-specific dependencies in ovarian cancer. Proceedings of the National Academy of Sciences of the United States of America 108**:** 12372-12377

Cohen J (1988) Statistical power analysis for the behavioral sciences (2 ed.): Lawrence Erlbaum Associates

Fox N, Mathers N (1997) Empowering research: statistical power in general practice research. Family practice 14**:** 324-329

Freedman RS, Kudelka AP, Kavanagh JJ, Verschraegen C, Edwards CL, Nash M, Levy L, Atkinson EN, Zhang HZ, Melichar B et al (2000) Clinical and biological effects of intraperitoneal injections of recombinant interferon-gamma and recombinant interleukin 2 with or without tumor-infiltrating lymphocytes in patients with ovarian or peritoneal carcinoma. Clinical cancer research : an official journal of the American Association for Cancer Research 6**:** 2268-2278

Gautier L, Cope L, Bolstad BM, Irizarry RA (2004) affy--analysis of Affymetrix GeneChip data at the probe level. Bioinformatics 20**:** 307-315

George SL (1996) Reducing patient eligibility criteria in cancer clinical trials. Journal of clinical oncology : official journal of the American Society of Clinical Oncology 14**:** 1364-1370

Haibe-Kains B, Desmedt C, Loi S, Culhane AC, Bontempi G, Quackenbush J, Sotiriou C (2012) A three-gene model to robustly identify breast cancer molecular subtypes. Journal of the National Cancer Institute 104**:** 311-325

Kim J-H (2009) Estimating Classification Error Rate: Repeated Cross-validation, Repeated Hold-out and Bootstrap. Computational Statistics & Data Analysis 53**:** 3735-3745

Konavi R (1995) A Study of Cross-Validation and Bootstrap for Accuracy Estimation and Model Selection. In 14th international joint conference on Artificial intelligence pp 1137-1143.

Lawrenson K, Gayther SA (2009) Ovarian cancer: a clinical challenge that needs some basic answers. PLoS medicine 6**:** e25

Liu Y, Hayes DN, Nobel A, Marron JS (2008) Statistical Significance of Clustering for High-Dimension, Low-Sample Size Data. Journal of the American Statistical Association 103**:** 1281-1293

Lowe AW, Olsen M, Hao Y, Lee SP, Taek Lee K, Chen X, van de Rijn M, Brown PO (2007) Gene expression patterns in pancreatic tumors, cells and tissues. PloS one 2**:** e323

Luo B, Cheung HW, Subramanian A, Sharifnia T, Okamoto M, Yang X, Hinkle G, Boehm JS, Beroukhim R, Weir BA et al (2008) Highly parallel identification of essential genes in cancer cells. Proceedings of the National Academy of Sciences of the United States of America 105**:** 20380-20385

Mantovani A, Allavena P, Sica A, Balkwill F (2008) Cancer-related inflammation. Nature 454**:** 436-444

Monk BJ, Huang HQ, Burger RA, Mannel RS, Homesley HD, Fowler J, Greer BE, Boente M, Liang SX, Wenzel L (2012) Patient reported outcomes of a randomized, placebo-controlled trial of bevacizumab in the front-line treatment of ovarian cancer: A Gynecologic Oncology Group Study. Gynecologic oncology

Neve RM, Chin K, Fridlyand J, Yeh J, Baehner FL, Fevr T, Clark L, Bayani N, Coppe JP, Tong F et al (2006) A collection of breast cancer cell lines for the study of functionally distinct cancer subtypes. Cancer Cell 10**:** 515-527

Perou CM, Jeffrey SS, van de Rijn M, Rees CA, Eisen MB, Ross DT, Pergamenschikov A, Williams CF, Zhu SX, Lee JC et al (1999) Distinctive gene expression patterns in human mammary epithelial cells and breast cancers. Proceedings of the National Academy of Sciences of the United States of America 96**:** 9212-9217

Prat A, Parker JS, Karginova O, Fan C, Livasy C, Herschkowitz JI, He X, Perou CM (2010) Phenotypic and molecular characterization of the claudin-low intrinsic subtype of breast cancer. Breast cancer research : BCR 12**:** R68

Syrjanen K, Syrjanen S (2013) Detection of human papillomavirus in sinonasal papillomas: Systematic review and meta-analysis. The Laryngoscope 123**:** 181-192

The Cancer Genome Atlas Research Network (2011) Integrated genomic analyses of ovarian carcinoma. Nature 474**:** 609-615

Tothill RW, Tinker AV, George J, Brown R, Fox SB, Lade S, Johnson DS, Trivett MK, Etemadmoghadam D, Locandro B et al (2008) Novel molecular subtypes of serous and endometrioid ovarian cancer linked to clinical outcome. Clinical cancer research : an official journal of the American Association for Cancer Research 14**:** 5198-5208

Verhaak RG, Tamayo P, Yang JY, Hubbard D, Zhang H, Creighton CJ, Fereday S, Lawrence M, Carter SL, Mermel CH et al (2013) Prognostically relevant gene signatures of high-grade serous ovarian carcinoma. The Journal of clinical investigation 123**:** 517-525

Virtanen C, Ishikawa Y, Honjoh D, Kimura M, Shimane M, Miyoshi T, Nomura H, Jones MH (2002) Integrated classification of lung tumors and cell lines by expression profiling. Proceedings of the National Academy of Sciences of the United States of America 99**:** 12357-12362

2nd Editorial Decision 19 March 2013

Thank you for the submission of your revised manuscript to EMBO Molecular Medicine. We have now received the enclosed reports from the referees that were asked to re-assess it. As you will see, the reviewers are now globally supportive and I am pleased to inform you that we will be able to accept your manuscript pending the following final amendments:

- please modify the text as suggested by both referees as they do still find many overstatements and discrepancies.

- please provide the supplementary tables as excel files or, alternatively, combine all tables and supplemental information into one single PDF file (see below).

- please provide individual figure files (see below). You may want to rearrange the different panels to maximize space in order to provide bigger panels (all labels are small-when the figures will be reduced to fit in the published paper, labels will be unreadable.)

- next generation sequencing dataset also needs to be deposited in database and an accession number provided.

- please provide an ethical statement for the use of clinical samples (see our author's guidelines for help)

I look forward to reading a new revised version of your manuscript as soon as possible.

***** Reviewer's comments *****

Referee #1 (General Remarks):

This resubmitted manuscript is a major improvement over the initial submission. The additional experiments performed, such as including more cell lines for shRNA screens, add greater robustness to the results. A tendency for overstatements and discrepancies/incompleteness in figures has been retained and these should be corrected.
- In troduction: 'However, due to varied samples sizes and analytical criteria, reported subtypes of EOC differ'. The three largest and most prominent studies in this field (Tothill, TCGA, Verhaak) show remarkable overlap between the transcriptional subtypes identified. This does not implicate there is no room for further refinement, but the similarities in literature are not properly acknowledged.
- The TCGA data set (which represents a third of the total data set) is not referenced in the 'Molecular heterogeneity of epithelial ovarian cancer' section
- Figure 1C - not possible to distinguish cell lines from tumor samples. What is 'other'? It is unclear how these samples were dealt with in the subsequent analyses such as survival analysis?
- The limitations of meta-analysis should be taken into consideration. Clinical information is based on diagnosis of each dataset and there is a possibility that diagnosis criteria varied in different studies. Especially, the authors should deal with pathological information carefully (histologic type and grade) because these information is not based on central pathological review. Therefore, the authors must bear in mind that combined data includes pathologically misdiagnosed samples.
- Although the study is focused on epithelial ovarian cancer, the distribution of four major histologic types (serous, endometrioid, clear cell, mucinous) in this microarray meta-analysis is not representative from what is observed clinically. This discrepancy seems to influence the results.
- "We also noted that the TCGA molecular subtyping lacked a Stem-B/C6 population (Suppl. Fig. 3B; Suppl. Text)." This seems an unnecessary comment as only high-grade serous ovarian cancer samples were included in TCGA. The use of the word 'lacking' implies that the analysis performed in TCGA was incomplete. "Stem-B" is basically composed of non-serous samples.
- "Using 260 samples from the validation set (GSE19829 [n = 28], GSE30311 [n = 47] and GSE26712 [n = 185]) for which patient outcome information was supplied (Konstantinopoulos et al, 2010), the Kaplan-Meier analysis on the BinReg-predicted molecular subtypes revealed a similar

pattern of patient prognoses with that of the original consensus clustering analysis (p = 0.0372 by the log-rank test; Fig. 1B; Suppl. Fig. 7D)." This description is not proper. Stem-B subtype showed good prognosis in the training set (Fig.1B) but poor prognosis in the validation set (Suppl. Fig. 7D).
- "The ss-GSEA analysis of 1,538 samples using 6,898 gene sets (GSEA databases Suppl. Table 6) revealed a subtype-specific enrichment of 207 gene sets (Fig. 1C; Suppl. Table 7)". Unfortunately, reader will not be able to obtain useful information in Fig.1C because there are no annotations about pathway.
- "This was performed using microarrays of representative samples for each subtype." To understand the result easily, the authors should add the number of core samples. It should be explained why the classification accuracy of the core samples was worse than that of non-core samples.
- Table 1: The authors described sample size as "Total n=537" in Table 1 (Page 41). However, total number of samples described in each clinical variable is "539".
- The pathway/ssGSEA analysis of cell lines seems a bit redundant, as by the nature of the comparison to tumor sample profiles, it seems logical that the associated pathways are highly similar.
- The concept of RNAi screens and synthetic lethality is based on the presence of genomic variation that creates cancer specific vulnerabilities. The use of various cell lines as a model for epithelial ovarian carcinoma, including PA-1, would be particularly useful when these are shown to genomically resemble ovarian carcinoma, harboring (like the majority of ovarian cancer) TP53 mutations and a vastly reorganized genome, with amplifications typical for ovarian cancer such as MYC, MECOM, Cyclin E1, and so forth. The Cell Line Encyclopedia data set may be of help to the authors; at least A2780, OVCAR-3 and SKOV3 are available there. PA-1 is acknowledged as originating from a teratocarcinoma but at a strange position. It would make sense to either describe this at first mention, or in the discussion section.
- 'Of note, a previous study of 489 samples could not correlate their molecular classification with patient overall survival (The Cancer Genome Atlas Research Network, 2011)'. As noted above the TCGA sample cohort includes only serous cases and is therefore not comparable to the current study. Moreover, later studies using the same TCGA data set (PMID 23257362) showed strong associations with outcome.
- 'The development of a decision tree model,17 utilizing the Stem-A and Epi-B predictors, might be a promising application to triage approximately half of the serous EOC patients for outcome prediction.' This is extremely speculative as no alternative therapeutic modalities are available for worse performing cases. Moreover, it is not clear that the classification system from this study outperforms that of previously reported classifiers (PMIDs 22203759, 22505474, and again 23257362).
- The authors note that microtubule stabilizers such as paclitaxel and docetaxel are commonly used in the treatment of ovarian cancer. Does their meta-data set have the clinical annotation to show that these drugs are particularly effective in their Stem-A class? Detailed drug information is available from TCGA.

Referee #3 (Comments on Novelty/Model System):

The inadequacies of in vitro models were addressed in the manuscript.

Referee #3 (General Remarks):

The authors have addressed most of my concerns. The remaining concerns are relatively minor:

1) Some overstatement/misstatement of results remain. For example:
a. The authors state that (Page 12, 1st paragraph), "Epi-A and Epi-B cell lines had longer population doubling times and decreased colony-forming ability, corresponding to the less aggressive behavior of clinical tumors. Overall these cell lines can serve as good experimental models for each molecular subtype." Those interpretations appear to be over stated. While in vitro proliferation rates

and anchorage independent growth can correlate with in vivo tumor behavior this language should softened to reflect the experimental findings directly.
b. First line on page 10: please remove "precisely" in "precisely dissects ovarian serous carcinoma heterogeneity".
c. Page 14: "Relying on a stringent criterion (>= 20% growth suppression..."-20% does not seem so stringent! Please just rephrase.
d. Page 16: "For the first time, using a genome-wide shRNA screen, we revealed that subtype-matched cell lines have distinct molecular pathways." I would change "revealed" to "found" and "molecular pathways" to "vulnerabilities".
e. Later on page 16, the words "unequivocally" and "inevitable" seem unnecessary overstatements.
f. Similarly, on page 17, "confirmed by shared cell functions" is strong and could be changed to "further supported by shared cell functions."
2) While the overall trends can be discerned, the resolution of Fig. 2B is too low to properly evaluate the data. Particularly the "Clinical sample probability" and "AUC" panels. The resolution on Supp Fig 1a is also too low.

2nd Revision - authors' response 03 April 2013

We would like to thank the Editor for being able to accept our revised manuscript entitled "Functional Genomics Identifies Five Distinct Molecular Subtypes with Clinical Relevance and Pathways for Growth Control in Epithelial Ovarian Cancer". We are also very grateful to the Referees for their support of the revised version of our study. We have further revised the manuscript according to the suggestions by the Editor and the Referees as follows:

- Please modify the text as suggested by both referees as they do find still too many overstatements and discrepancies.

We have modified the manuscript accordingly as suggested by both Referees so as to remove overstatements and discrepancies.

- please provide the supplementary tables as excel files or, alternatively, combine all tables and supplemental information into one single PDF file (see below)

We now upload supplementary tables as an Excel file (*.xls).

- Please provide individual figure files (see below). You may want to rearrange the different panels to maximize space in order to provide bigger panels (all labels are small-when the figures will be reduced to fit in the published paper, labels will be unreadable.)

According to the guidelines, the figures have been formatted with a resolution of 300 dpi.

- next generation sequencing dataset also needs to be deposited in database and an accession number provided.

We have deposited the dataset for next-generation sequencing analysis of the shRNA screen at Gene Expression Omnibus (GEO), with the accession number GSE45420.

- please provide an ethical statement for the use of clinical samples (see our author's guidelines for help)

Our ethical statement for the use of clinical samples is given here:

All publicly available datasets were included at the time of the study (April 2010), and compiled with an Oslo cohort dataset (Ben Davidson and Jahn Nesland), obtained under the ethics approval from the Health Region of South-Eastern Norway (# 04300).

The statement was included in the section of "Acknowledgements".

We appreciate the effort of the Referees, and have further addressed the points raised by the Referees in this second revised manuscript. The replies to the Referees are enclosed in point-by-point form.

Referee #1

This resubmitted manuscript is a major improvement over the initial submission. The additional experiments performed, such as including more cell lines for shRNA screens, add greater robustness to the results. A tendency for overstatements and discrepancies/incompleteness in figures has been retained and these should be corrected.

- Introduction: 'However, due to varied samples sizes and analytical criteria, reported subtypes of EOC differ'. The three largest and most prominent studies in this field (Tothill, TCGA, Verhaak) show remarkable overlap between the transcriptional subtypes identified. This does not implicate there is no room for further refinement, but the similarities in literature are not properly acknowledged.

> We have modified the sentence on Page 5 of the manuscript such that the similarity of subtypes is properly acknowledged. The sentence was modified to “However, due to varied samples sizes and analytical criteria, the reported subtypes of EOC were similar but not completely the same…”.

- The TCGA data set (which represents a third of the total data set) is not referenced in the 'Molecular heterogeneity of epithelial ovarian cancer' section

> Even in the original manuscript, the TCGA paper was referenced in the section of “Molecular heterogeniety of epithelial ovarian cancer”. Nevertheless, to acknowledge the size of TCGA data, we added the description of “Among the 16 datasets, TCGA data was the largest in the sample number (n = 406; 26.4% of all samples).” into the section of “Molecular heterogeniety of epithelial ovarian cancer” on Page 7.

- Figure 1C - not possible to distinguish cell lines from tumour samples. What is 'other'? It is unclear how these samples were dealt with in the subsequent analyses such as survival analysis?

> Fig. 1C is a result from ss-GSEA using the data of all 1,538 clinical samples without any cell lines. The co-clustering analysis of clinical samples with cell lines is shown in Suppl. Fig. 10A. Since we did not include any cell-line arrays for the initial consensus clustering analysis shown in Fig. 1A, we added the description of “Also note that none of cultured cell-line data was included in this analysis.” in the Figure Legend of Fig. 1A to avoid unnecessary confusion.
>
> “Other” was used to indicate the unclassified samples not grouped in any of the five subtypes in the initial consensus clustering analysis in Fig. 1A. They were not included in following statistical analyses for characterization of the molecular subtypes. We have defined what “other” means in the figure legends for Figs. 1C and 1D, and Suppl. Figs. 4, 8C and 9. We have also amended the description in the section of “Consensus clustering” in “Materials and Methods” to clearly describe how “other” samples were dealt with in following analyses.

- The limitations of meta-analysis should be taken into consideration. Clinical information is based on diagnosis of each dataset and there is a possibility that diagnosis criteria varied in different studies. Especially, the authors should deal with pathological information carefully (histologic type and grade) because these information is not based on central pathological review. Therefore, the authors must bear in mind that combined data includes pathologically misdiagnosed samples.

> We thank the Referee for pointing out a caveat and limitation of meta-analysis. As the

Referee mentions, the clinicopathological information obtained with each dataset was neither standardized nor centrally reviewed across the datasets; therefore, there might be pathologically misdiagnosed samples included. This might have some potential impact on the Stem-B subgroup since we identified this subgroup as a group exhibiting “less serous” features. We have added the description into the “Result” section of “Correlation of subtype with clinicopathological parameters” in the main text as well as into the section of “Designation of molecular subtypes” in the “Suppl. Text” accordingly.

- Although the study is focused on epithelial ovarian cancer, the distribution of four major histologic types (serous, endometrioid, clear cell, mucinous) in this microarray meta-analysis is not representative from what is observed clinically. This discrepancy seems to influence the results.

The distribution of histologies in our compiled data was serous (82.8%), mucinous (1.8%), clear cell (1.6%), endometrioid (6.2%), and low-malignant potential (LMP) (4.0%), in contrast to the actual incidence rates of serous (55~65%), mucinous (~3%), clear cell (~5%), endometrioid (~10%), and LMP (15~20%) (Soslow, 2008; Jelovac et al. 2011 and Acs, 2005). We agree with the Referee that this discrepancy in the histological distribution may influence the clustering results. To some extent, we indeed discussed this influence already in the section of “Designation of molecular subtypes” in “Suppl. Text” of the first revised manuscript. Nevertheless, we have now also included this notion in the section of “Molecular heterogeneity of epithelial ovarian cancer” in “Results” of the main text.

References:

Soslow RA. Histologic subtypes of ovarian carcinoma: an overview. Int J Gynecol Pathol. 2008 Apr;27(2):161-74.

Jelovac D, Armstrong DK. Recent progress in the diagnosis and treatment of ovarian cancer. CA Cancer J Clin. 2011 May-Jun;61(3):183-203.

Acs G. Serous and mucinous borderline (low malignant potential) tumors of the ovary. Am J Clin Pathol. 2005 Jun;123 Suppl:S13-57.

- "We also noted that the TCGA molecular subtyping lacked a Stem-B/C6 population (Suppl. Fig. 3B; Suppl. Text)." This seems an unnecessary comment as only high-grade serous ovarian cancer samples were included in TCGA. The use of the word 'lacking' implies that the analysis performed in TCGA was incomplete. "Stem-B" is basically composed of non-serous samples.

As suggested by the Referee, we have rephrased the sentence on Page 9 to “We also noted that TCGA molecular subtyping did not include a Stem-B/C6 population".

- "Using 260 samples from the validation set (GSE19829 [n = 28], GSE30311 [n = 47] and GSE26712 [n = 185]) for which patient outcome information was supplied (Konstantinopoulos et al, 2010), the Kaplan-Meier analysis on the BinReg-predicted molecular subtypes revealed a similar pattern of patient prognoses with that of the original consensus clustering analysis (p = 0.0372 by the log-rank test; Fig. 1B; Suppl. Fig. 7D)." This description is not proper. Stem-B subtype showed good prognosis in the training set (Fig.1B) but poor prognosis in the validation set (Suppl. Fig. 7D).

As the Referee noted, the similarity of the patient outcomes between the training and validation sets was observed in the subtypes except for Stem-B. We already discussed the discrepancy of Stem-B tumours in the section of “Designation of molecular subtypes” in “Suppl. Text” of the first revised manuscript. Nevertheless, we have now included this notion in the “Results” section of “Predictive framework for EOC subtype classification” and also modified the description in the Figure Legends for Suppl. Figs. 7D and 8D.

- "The ss-GSEA analysis of 1,538 samples using 6,898 gene sets (GSEA databases Suppl. Table 6)

revealed a subtype-specific enrichment of 207 gene sets (Fig. 1C; Suppl. Table 7)". Unfortunately, reader will not be able to obtain useful information in Fig.1C because there are no annotations about pathway.

We have modified Fig. 1C by additionally annotating several pathways that we mentioned in the manuscript. To maintain consistency, we also annotated the pathways in Fig. 2C.

- "This was performed using microarrays of representative samples for each subtype." To understand the result easily, the authors should add the number of core samples. It should be explained why the classification accuracy of the core samples was worse than that of non-core samples.

The number of core samples (n = 50 per subtype) and the remaining samples not used in building predictive model (n = 1,413) were added to the "Predictive framework for EOC subtype classification" section on Page 10 in the main text. We also explained the possible reason why the classification accuracy of the core samples was worse than that of non-core samples in the "Classification accuracy of the core samples and of non-core samples" in the "Suppl. Text".

- Table 1: The authors described sample size as "Total n=537" in Table 1 (Page 41). However, total number of samples described in each clinical variable is "539".

We thank the Referee for indicating this typo. The number has been change to n = 539 in Table 1.

- The pathway/ssGSEA analysis of cell lines seems a bit redundant, as by the nature of the comparison to tumour sample profiles, it seems logical that the associated pathways are highly similar.

We believe that the results from the pathway analyses (Fig. 2C) are able to support the expression similarity between clinical tumour and cell-line subtypes by indicating biological interpretations and, as such, we kept it in the revised manuscript though it may seem a bit redundant.

- The concept of RNAi screens and synthetic lethality is based on the presence of genomic variation that creates cancer specific vulnerabilities. The use of various cell lines as a model for epithelial ovarian carcinoma, including PA-1, would be particularly useful when these are shown to genomically resemble ovarian carcinoma, harbouring (like the majority of ovarian cancer) TP53 mutations and a vastly reorganized genome, with amplifications typical for ovarian cancer such as MYC, MECOM, Cyclin E1, and so forth. The Cell Line Encyclopaedia data set may be of help to the authors; at least A2780, OVCAR-3 and SKOV3 are available there. PA-1 is acknowledged as originating from a teratocarcinoma but at a strange position. It would make sense to either describe this at first mention, or in the discussion section.

We appreciate the Referee's suggestion. Following this, we performed statistical analysis using genomic information from TCGA and Cancer Cell Line Encyclopaedia (CCLE).

First, information of genomic aberrations of copy number changes (63 amplified genes including *MYC*, *MECOM* and *CCNE1*, 50 genes with deletion such as *RB1* and *NF1*) and somatic mutations (8,431 genes exemplified by *TP53*, *BRCA1*) observed in serous ovarian cancer were collected from TCGA database. For somatic mutations, Fisher exact test identified 11 genes as exhibiting subtype-selective enrichment in clinical tumours. The genes include *TP53* (less frequently mutated in Stem-A; $p = 0.009$), FAT3 (less frequently mutated in Mes; $p = 0.0193$) and *SPTB* (more frequently mutated in Epi-B; $p = 0.0009$).

Second, the genomic alteration data of the ovarian cancer cell lines, where we have subtype information (4 Epi-A, 1 Epi-B, 9 Mes, 7 Stem-A and 2 Stem-B; n = 23), was extracted from CCLE. Subsequently, we compared the genomic aberration pattern across the subtypes between TCGA clinical samples and CCLE cell lines. Due to the limited number of the cell

lines, however, only two genes (*MIER2* and *APBA3*) were identified to be less frequently deleted in Mes subtype (p = 0.0189 and p = 0.0072, respectively) with difficulty in understanding this biological significance.

As we mentioned in the first Rebuttal letter, we are currently assembling a database including genomic alterations, miRNA, and methylation patterns for the panel of cell lines. We are hoping to obtain significant results in the coming months to be part of a follow-up study. Thus, these findings are not reported in the current manuscript.

The acknowledgement of PA-1 being a teratocarcinoma line was moved to the first time it is mentioned in the Results section of "Genome-wide shRNA screens identified subtype-specific growth-promoting genes" on Page 12 as suggested by the Referee.

- 'Of note, a previous study of 489 samples could not correlate their molecular classification with patient overall survival (The Cancer Genome Atlas Research Network, 2011)'. As noted above the TCGA sample cohort includes only serous cases and is therefore not comparable to the current study. Moreover, later studies using the same TCGA data set (PMID 23257362) showed strong associations with outcome.

First, by the sentence, we simply indicated the fact that TCGA researchers observed no correlation between patient outcomes (overall survival: OS) and the TCGA subtypes in their cohort. Importantly, the cohort showed no correlation with the patient OS based on our subtyping scheme unlike the other cohorts (Fig. R2; please note that only high-grade serous samples were included for fair comparison). This implies TCGA cohort contained 'different' patient populations to some extent from those of the other cohorts. Therefore, we described as 'This is perhaps derived from an inevitable bias internal to the cohort' in the original manuscript.

Second, we should mention one important point that the TCGA paper mentioned by the Referee (PMID 23257362) was not published at the time we submitted our manuscript to EMBO Molecular Medicine. In the paper, Verhaak *et al.* utilized "CLOVAR (Classification of Ovarian Cancer) subtype" that was a refined subtype signature from the original TCGA scheme. By CLOVAR subtype, the authors indeed observed two associations between the subtypes and outcomes as this Referee mentioned:

1) It is important to point out that the strong association in OS was observed with the populations, in which the vast majority was derived from the other cohorts than TCGA (64 patients from TCGA cohort and 815 patients from 6 published studies) ($p < 0.0001$ in Fig. 2B of PMID 23257362).
2) A significant difference in recurrence-free survival (RFS) was also observed between patients in the TCGA cohort whose tumor samples expressed either the immunoreactive or the mesenchymal signature ($p = 0.037$ in Suppl. Fig. 2 of PMID 23257362).

Considering there is similarity between our subtyping scheme with the ones by Tothill and TCGA, the correlation by CLOVAR subtyping seemingly indicates that the OS can be correlated with any of the three molecular subtyping strategies with the data from many cohorts but not from the TCGA cohort.

Based on this, we have added the description of "although a more recent study correlated two TCGA subtypes with relapse-free survival using the same cohort".

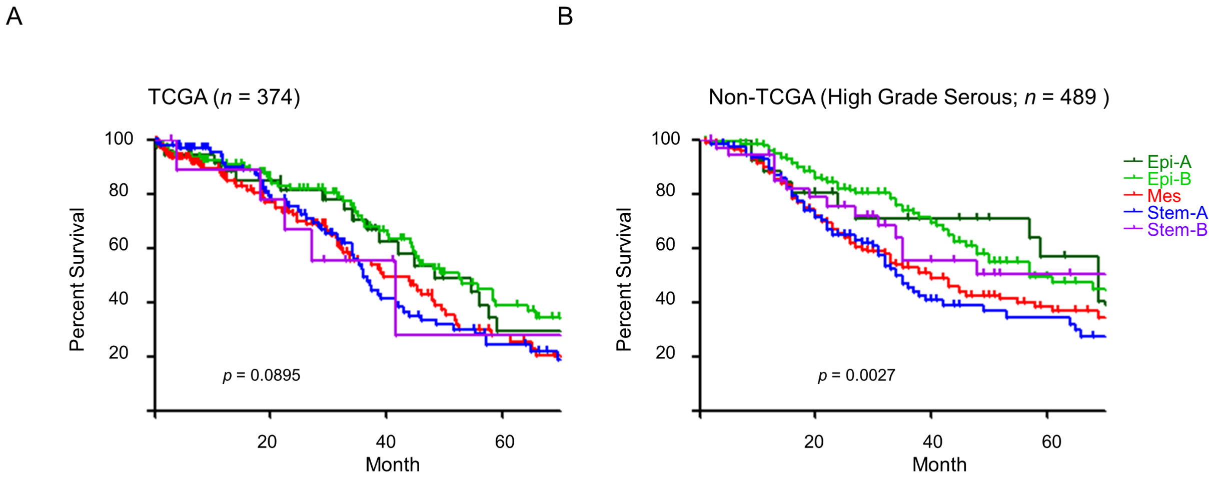

Fig. R2: Overall survival of (A) TCGA and (B) cohort other than TCGA (high-grade serous samples only) by the proposed molecular subtyping. Colour code: Epi-A, dark green; Epi-B, light green; Mes, red; Stem-A, blue; Stem-B, purple. *P*-values were computed by log-rank test.

- 'The development of a decision tree model, utilizing the Stem-A and Epi-B predictors, might be a promising application to triage approximately half of the serous EOC patients for outcome prediction.' This is extremely speculative as no alternative therapeutic modalities are available for worse performing cases. Moreover, it is not clear that the classification system from this study outperforms that of previously reported classifiers (PMIDs 22203759, 22505474, and again 23257362).

By the sentence we meant to simply discuss the possibility of using the subtype signatures with our scheme to build a predictive model for patient outcomes. We did not intend to indicate our molecular subtyping was immediately applicable for that particular purpose with the current condition. Nevertheless, so as to avoid having too speculative discussion as indicted by the Referee, we have removed the sentences of “To date, the incorporation of molecular subtype prediction into clinical decisions has not been realized. The development of a decision tree model, utilizing the Stem-A and Epi-B predictors, might be a promising application to triage approximately half of the serous EOC patients for outcome prediction.” from the “Discussion” of the second revised manuscript.

- The authors note that microtubule stabilizers such as paclitaxel and docetaxel are commonly used in the treatment of ovarian cancer. Does their meta-data set have the clinical annotation to show that these drugs are particularly effective in their Stem-A class? Detailed drug information is available from TCGA.

We thank the Referee for this suggestion. We have examined the information pertaining to clinical response to chemotherapeutic drugs from TCGA database (n = 526). In the database, there were 158 patients treated with adujuvant regimens including paclitaxel or docetaxel. A Fisher exact test revealed no statistically significant difference between Stem-A and the other subtypes in the response (p = 0.1215; Fig. R3), although there is a trend, in which Stem-A seems to have more complete response. Since the difference was not statistically significant, we do not include this observation in the revised manuscript.

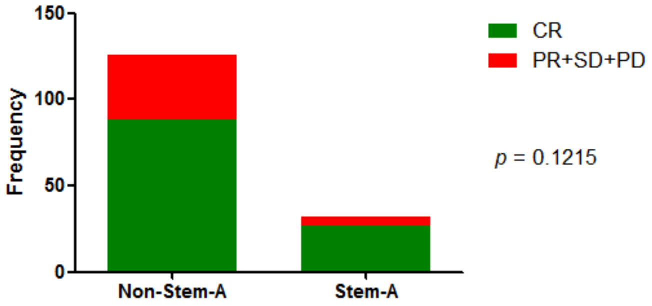

Fig. R3: Contingency plot of clinical response to mitotic inhibitors (paclitaxel and docetaxel) for Stem-A (n = 32) and Non-Stem-A subtypes (n = 126). Frequency is on the *y*-axis. The *p*-value shown is computed by Fisher exact test. Abbreviation: Stem-A, Stem-like A; CR, complete response; PR, partial response; SD, stable disease; PD, progressive disease.

Referee #3 (Comments on Novelty/Model System):

The inadequacies of in vitro models were addressed in the manuscript.

Referee #3 (General Remarks):

The authors have addressed most of my concerns. The remaining concerns are relatively minor:

1) Some overstatement/misstatement of results remain. For example:

a. The authors state that (Page 12, 1st paragraph), "Epi-A and Epi-B cell lines had longer population doubling times and decreased colony-forming ability, corresponding to the less aggressive behaviour of clinical tumours. Overall these cell lines can serve as good experimental models for each molecular subtype." Those interpretations appear to be over stated. While in vitro proliferation rates and anchorage independent growth can correlate with in vivo tumour behaviour this language should softened to reflect the experimental findings directly.

We have changed the sentence on Page 12 to "Epi-A and Epi-B cell lines had longer population doubling times and decreased colony-forming ability, which may reflect the less aggressive behaviour of clinical tumours. Overall these cell lines can serve as good experimental models for each molecular subtype."

b. First line on page 10: please remove "precisely" in "precisely dissects ovarian serous carcinoma heterogeneity".

We have removed "precisely" of the sentence on Page 10 as suggested by the Referee.

c. Page 14: "Relying on a stringent criterion (>= 20% growth suppression..."-20% does not seem so stringent! Please just rephrase.

We have rephrased the sentence on Page 14 as suggested by the Referee to "Relying on criterion of >= 20% growth suppression …"

d. Page 16: "For the first time, using a genome-wide shRNA screen, we revealed that subtype-matched cell lines have distinct molecular pathways." I would change "revealed" to "found" and "molecular pathways" to "vulnerabilities".

As suggested by the Referee, we have revised the sentence on Page 16 to "For the first time, using a genome-wide shRNA screen, we found that subtype-matched cell lines have distinct vulnerabilities"

e. Later on page 16, the words "unequivocally" and "inevitable" seem unnecessary overstatements.

We have removed the words "unequivocally" and "inevitable" from page 16 so as not to overstate our findings.

f. Similarly, on page 17, "confirmed by shared cell functions" is strong and could be changed to "further supported by shared cell functions."

We have replaced the sentence as suggested by the Referee on page 17.

2) While the overall trends can be discerned, the resolution of Fig. 2B is too low to properly evaluate the data. Particularly the "Clinical sample probability" and "AUC" panels. The resolution on Supp Fig 1a is also too low.

We have enlarged the size of our figures and increased the resolution of all the figures from 96 dpi to 300 dpi.

© EMBO
