# Supplementary material for: Functional genomics identifies five distinct molecular subtypes with clinical relevance and pathways for growth control in epithelial ovarian cancer
Source: EMBO Mol Med. 2013 May 13;5(7):983–98. doi: 10.1002/emmm.201201823 (PMC3721473; doi:10.1002/emmm.201201823)
Supplement: Supplementary file 2 [file emmm0005-0983-SD2.pdf]

Supplemental Information

Functional Genomics Identifies Five Distinct Molecular Subtypes with Clinical Relevance and Pathways for Growth Control in Epithelial Ovarian Cancer

Tuan Zea Tan, Qing Hao Miow, Ruby Yun-Ju Huang, Meng Kang Wong, Jieru Ye, Jieying Amelia Lau, Meng Chu Wu, Luqman Hakim Bin Abdul Hadi, Richie Soong, Mahesh Choolani, Ben Davidson, Jahn Nesland, Ling-Zhi Wang, Noriomi Matsumura, Masaki Mandai, Ikuo Konishi, Boon-Cher Goh, Jeffrey T. Chang, Jean-Paul Thiery, Seiichi Mori

Table of Contents:

Supplemental Text

- Designation of molecular subtypes
- Discordance of molecular subtype prediction
- Classification accuracy of core and non-core samples
- Association of molecular subtypes with patient outcomes
- Cell line subtype identification by consensus clustering
- Identification of Epi-B cell lines
- Pathways affected in silencing PA-1-specific genes

Supplemental Figure

Suppl. Fig. 1. Removing the batch effect of combined expression microarray data for epithelial ovarian cancer.

Suppl. Fig. 2. Statistical power plot.

Suppl. Fig. 3. Additional information for the proposed ovarian cancer molecular subtype.

Suppl. Fig. 4. Subtype distribution.

Suppl. Fig. 5. *WT1* gene expression of ovarian cancer subtype.

Suppl. Fig. 6. Clinicopathological characterisation of molecular subtypes.

Suppl. Fig. 7. A diagnostic method to predict ovarian cancer subtype based on a Binary Regression model.

Suppl. Fig. 8. Diagnostic subtype prediction by SAM/ROC/ClaNC.

Suppl. Fig. 9. Three-way split cross validation using BinReg and ClaNC.

Suppl. Fig. 10. Identification of cell line subtype status.

Suppl. Fig. 11. Surface marker and interferon signature of Epi-B cell lines.

Suppl. Fig. 12. Subtype-specific amplified or depleted hairpins.

Suppl. Fig. 13. Pathways affected by silencing PA-1-specific genes.

Supplemental Table

Supplemental Materials and Method
- Principal component analysis
- Statistical power analysis
- Predictive modelling and validation by BinReg
- Predictive modelling and validation by ClaNC
- Three-way split cross-validation using BinReg and ClaNC
- Cell line panel
- Genome-wide RNAi screens for subtype-specific proliferation genes
- Pathway analysis in silencing PA-1-specific genes

Supplemental References

Supplemental Text

Designation of molecular subtypes

The Epithelial-A (Epi-A/Differentiated/C3) and Epithelial-B (Epi-B/Immunoreactive/C2) tumour clusters expressed epithelial cell markers, such as *CDH1* (*E-cadherin*), *EPCAM*, various keratin genes (*KRT*s) and *CD24*. The Mesenchymal (Mes/Mesenchymal/C1) tumour subtype predominantly expressed fibroblastic/mesenchymal genes, such as *PDGFRA*, *VCAM1*, *ZEB1*, *TWIST1*, and extracellular matrix genes, including collagen and *FN1*. The Stem-like-A (Stem-A/Proliferative/C5) and Stem-like-B (Stem-B/C6) tumour clusters did not share many gene markers, but expressed typical markers for epithelial stem cells: *LGR5* and *PROM1* (*CD133*), respectively (Fodde, 2009). Stem-A tumours also expressed more *MYCN*, *NCAM*, *CDH2* (*N-cadherin*) and proliferation-related genes, suggesting neural characteristics. Epi-B and Mes tumours expressed inflammatory genes, such as multiple interferon down-stream genes, MHC class II genes and immunoglobulin genes (Fig. 1A; Suppl. Table 2; Suppl. Figs. 11A and 11B). The proportion of each subtype in all 1,538 samples was 8.8% for Epi-A (n = 135), 25.5% for Epi-B (n = 392), 26.7% for Mes (n = 412), 20.5% for Stem-A (n = 315), and 12.3% for Stem-B (n = 189); 6.2% remained unclassified (n = 95).

We noted that three datasets (GSE10971, GSE14407 and GSE18520) contained 78 samples taken by laser capture microscopy that were highly enriched for carcinoma cells by maximum elimination of contaminating stroma. The distribution of each subtype was consistent with that of the entire collection (Epi-A, 7.7%; Epi-B, 25.6%; Mes, 21.8%; Stem-A, 20.5%; Stem-B, 3.8%; and unclassified, 20.5% in 78 samples), implying that the subtypes are intrinsic to cancer cells, and not dependent on stromal cells.

A comparison of the subgrouping scheme in the current study with that of previous classifications by Tothill *et al.* (Tothill et al, 2008) or by The Cancer Genome Atlas (TCGA) (The Cancer Genome Atlas Research Network, 2011) revealed that the Stem-B/C6 subtype was identified from the analyses of multi-histotype ovarian cancers by Tothill *et al.* and by us but not by TCGA in their analysis of only high-grade serous ovarian cancer (Suppl. Fig. 3B). On the other hand, in our analyses, most of the histotypes other than serous cancers were

classified into the Stem-B subtype (Suppl. Figs. 4B and 6A); although, these non-serous tumours shared "few molecular similarities". The inclusion of the other histotypes, therefore, may allow the identification of a unique subgroup in serous carcinomas. To see the effect of including the other histologies in our molecular classification (using a standard deviation cut-off of 1.05), we examined the lists of genes that were most variably expressed across the samples: 1) all samples used in the current study (n = 1,538), 2) serous carcinoma samples (n = 1,274) and 3) samples with histotypes other than serous carcinoma (n = 264). These lists showed a significantly higher overlap between all 1,538 and 1,274 serous samples (1,116 overlapped/1,138 genes in total; 98.1%), than between all 1,538 and 264 of the other histology samples (1,089 overlapped/1,311 genes in total; 83.1%). This difference in the gene lists indicates that the overall expression pattern with all 1,538 samples was influenced mainly by variation within the serous cancer samples, but to a lesser extent than that within the other histological types. The other histotypes might have been thus classified into the same molecular category as Stem-B, even though they exhibited distinct biological characteristics. The clinicopathological information obtained with each dataset was neither standardised nor centrally reviewed across the datasets; therefore, it is possible that pathologically misdiagnosed samples were included. However, it is also likely that the variation between serous and the other histologies contributed to the identification of the Stem-B subtype exhibiting "less serous" features. This notion was also supported by the fact that the expression level of *WT1* gene, a marker of serous adenocarcinoma of the ovary (Lawrenson & Gayther, 2009) was significantly lower in Stem-B tumours, irrespective of whether they were exhibiting other histotypes or were only serous ovarian carcinoma (Suppl. Fig. 5).

The Kaplan-Meier curves for the Stem-B subtype differed between the training and validation sets, while those curves for the other subtypes were consistent (Fig. 1B; Suppl. Figs. 7C, 7D, 8B and 8D). The small number of samples for Stem-B (n = 56 [BinReg] or 32 [ClaNC] samples with survival information) in the validation dataset might be responsible for this discrepancy. However, we also found that the serous samples included in the Stem-B

subtype have different overall survival rates between the training and validation datasets, implying possible heterogeneity within the subtype.

Discordance of molecular subtype prediction

Despite recent progress in the development of statistical models for cancer molecular subtyping with expression microarray data, there is still uncertainty about the reproducibility of these various classification algorithms because of the intrinsic nature of subtype identification where the true classification remains unknown (Haibe-Kains et al, 2012). Previously, Haibe-Kains *et al.* showed that the concordance between pairs of different classifiers varied from 49-86% (median: 68%) in the molecular subtyping of 5,715 breast cancer samples derived from 36 independent datasets (Haibe-Kains et al, 2012). Similarly, a concordance of 77.5% was observed between a centroid prediction and a *k*-means clustering in a breast cancer cohort with 412 samples (Calza et al, 2006), showing that the best methods achieve a concordance comparable to the 78.8% we observed in our study. By scrutinising the discrepancy in the subtype classification, we noted high rates of discordant assignments in distinguishing Epi-A from Epi-B or Stem-B, and Epi-B from Mes (Suppl. Table 8). This ambiguity may arise from shared biological properties between some of the subtypes; for example, all three Epi-A, Epi-B and Stem-B subtypes expressed epithelial markers, and many clinical samples with Epi-B or Mes subtype assignment expressed inflammatory cell markers (Fig. 1A; Suppl. Fig. 11A). A similar overlap was indeed observed in a breast cancer cohort between luminal A and luminal B subtypes, which shared a nuclear expression of oestrogen receptors (Calza et al, 2006). In fact, 82% of 489 ovarian cancer expression data of TCGA were assigned to more than one subtype in a cross-validation with ss-GSEA (Verhaak et al, 2013), implying there were transcriptionally overlapped features across the samples. We believe that the observed discordance is due to similarity in the biological properties of the clinical samples.

Classification accuracy of core and non-core samples

The classification accuracy of core samples was worse than non-core samples (Fig. 1D) primarily due to the difference in gene sets used for defining core samples, and the gene sets used to define subtype by BinReg. The gene components for each gene set were largely distinct, with a 9.8% overlap (116/1185 genes). Moreover, the silhouette width for the genes with variable expression was not correlated with that for the BinReg subtype signatures (Spearman correlation *rho* = 0.0194 and p = 0.4612). These findings are not inconsistent with our observation, in which the core samples were not always predicted as anticipated.

Association of molecular subtypes with patient outcomes

In addition to Table 1, which outlines the analysis of all histological ovarian cancer cases with age, stage, grade, metastasis status and molecular subtype status for overall survival rate, we further performed four additional Cox hazardous regression analyses (Suppl. Table 5A) as described below:

1) Serous cancer cases with age, stage, grade, metastasis and molecular subtype status, based on the overall survival rate (OS) (Suppl. Table 5B).

: The Stem-A or Epi-B subtype was found to be an independent prognostic factor from multiple clinical characteristics for serous ovarian cancer patients.

2) Serous cancer cases with age, stage, grade, metastasis status and molecular subtype status, based on the progression-free survival rate (PFS) (Suppl. Table 5E).

: The molecular subtype was not independently correlated with PFS for serous ovarian cancer.

3) All histology cancer cases with age, stage, grade, metastasis status, surgical status and molecular subtype status, based on OS (Suppl. Table 5C).

: The limited number of cases with complete information prevented us from identifying even the debulking status as an independent prognostic factor with statistical significance. Similarly, none of the molecular subtype was found as an independent prognostic factor.

4) All histology cancer cases with status for debulking surgery or molecular subtypes, based on OS (Suppl. Table 5D).

: When we examined only the debulking surgery status and the molecular subtype, Epi-B and Stem-A were found to be significant prognostic factors independent of the debulking status.

In summary, the Stem-A and Epi-B subtypes are consistently identified as prognostic factors that are independent of multiple clinical parameters and status, which include the status for surgery in the overall survival rate (Table 1; Suppl. Tables 5A, 5B and 5D).

Cell line subtype identification by consensus clustering

Four independent datasets of ovarian cancer cell lines from Duke University (42 cell lines), Kyoto University (37 cell lines), National University of Singapore (34 cell lines) and Lawrence Berkeley National Laboratory (29 cell lines) were analysed (Guan et al, 2007; Matsumura et al, 2011). The data for a total of 142 cell lines were compiled and analysed with the data of 1,142 core clinical samples in consensus clustering, without considering the histological origin of the cell line. Realising that the identified subclass labelling for cell lines did not fully capture the pattern of clinical samples, this labelling was then used as a tentative assignment for cell line subtypes for subsequent clustering analysis. After identification of the subtype-specific marker genes using the "cell-line only" expression data with SAM and ROC (Tusher et al, 2001), a consensus clustering was again performed relying on the selected gene sets (Suppl. Fig. 10A). The approach of the co-clustering analysis (Lowe et al, 2007; Perou et al, 1999; Prat et al, 2010; Virtanen et al, 2002) yielded a stable subtype classification for the cell lines with reasonable similarity to that of the clinical samples (Suppl. Fig. 10C), and achieved a 100% consistency in subtype classification for biological replicates of 28 cell lines that originated from the same source (Suppl. Table 9). This is in contrast to the approach of predicting the cell line subtype with BinReg or ClaNC, where the consistency in subtype classification was 67.9% (19/28 cell lines) amongst the biological replicates (Suppl. Table 9). Based on this observation, co-clustering was adopted instead of a prediction approach for

subtype assignment of the ovarian cancer cell lines. We performed a silhouette analysis and a SigClust (Liu et al, 2008) to confirm the validity of the cell line subtypes (Fig. 2A). In addition, to confirm the expression similarity between cell lines and clinical samples for each subtype, Spearman correlation map was constructed to measure the closeness in gene expression between the cell lines and clinical samples (Suppl. Fig. 10C). Furthermore, BinReg was adopted to validate the subtype assignment of the cell lines (Fig. 2B) (Gatza et al, 2010).

A co-consensus clustering of the cell lines and the serous tumour samples was performed, and yielded a result that was highly concordant (137/142, 96.5%) with that of the original analysis. We noted that the five discordant arrays included three biological replicates of the TYK-nu cell line and one of its derivatives (TYK-nu CisR) (data not shown), which were originally grouped into the Mes subtype but were clustered with Stem-A serous tumours when clustering with serous tumours only. Nevertheless, this highly concordant assignment demonstrates the robustness of the co-clustering method and implies a very modest influence of the other histologies when included with serous tumour samples.

Identification of Epi-B cell lines

Since Epi-B clinical samples were characterised by contaminating inflammatory-cell gene expression, it seemed difficult to identify Epi-B cell line counterparts. Nevertheless, the identified Epi-B cell lines indeed shared transcriptional profiles with the Epi-B clinical tumour samples. The similarity in the expression was confirmed with the BinReg analyses at gene and pathway levels (Figs. 2B and 2C), as well as with the Spearman correlation map (Suppl. Fig. 10C). It is plausible that the similarity between cell lines and clinical samples is intrinsic to tumour cells and not to contaminating inflammatory cells, as supported by several lines of evidence: 1) The Epi-B subtype of clinical samples consists of C2 and C4 according to the classification of Tothill and colleagues, as illustrated in Suppl. Fig. 3B (Tothill et al, 2008). C4 is a tumour cluster with fewer infiltrating cells than C2; therefore, the Epi-B subtype in clinical samples is not solely characterised by inflammatory cell infiltration (Fig.

1A). 2) Epi-B tumours are characterised not only by the marker gene expression of inflammatory cells, such as major histocompatibility complex (MHC) class II genes (*HLA-DMs*, *DOs*, *DPs*, *DQs* and *DRs*), but also by gene sets for epithelial cells and interferon (IFN) pathways, such as IFN-downstream genes (Fig. 1A; Suppl. Figs. 11A and 11B). It is important to note that Epi-B cell lines also retained a similar expression pattern in these subsets of genes that include even MHC class II genes (Suppl. Figs. 11A and 11B). This ectopic expression of MHC class II genes in Epi-B cell lines may be induced through the IFN signalling pathway, since intrinsic inflammatory pathways in cancer cells (such as TNF-α and IFNs) can be activated by tumorigenic events. Also, IFNs are reported to stimulate the expression of MHC genes in various cancer cells, including ovarian cancer *in vitro* and *in vivo* (Boyer et al, 1989; Freedman et al, 2000; Mantovani et al, 2008).

Pathways affected in silencing PA-1-specific genes

The ss-GSEA revealed the pathways affected by targeting each of the five PA-1 relevant genes in PA-1, OVCA433 and HeyA8. All siRNA transfections produced significant and appropriate silencing of the genes with 67.3-92.6% (median 83.9%) efficacy (Suppl. Fig. 13A). A total of 534 pathways were commonly altered across the three cell lines (Suppl. Fig. 13B; Suppl. Table 14). *GTF3C1* knockdown coincided with the down-regulation of the gene set *Reactome RNA Polymerase III Transcription* in all three cell lines pertaining to the function of *GTF3C1* as a regulatory component of RNA polymerase III transcription machinery (Kovelman & Roeder, 1992). Similarly, as one of the components required for microtubule nucleation (Fava et al, 1999; Moritz et al, 1995; Moritz et al, 1998), *TUBGCP4* knockdown resulted in down-regulation of the *Microtubule* gene set in the transcriptome. Common pathways for *NAT10, BLOC1S1* and *LRRC59* are listed in Suppl. Table 14. Thus, these examples indicate that this approach can connect a gene with a pathway as expected from the biological function of the gene.

Supplemental Figure

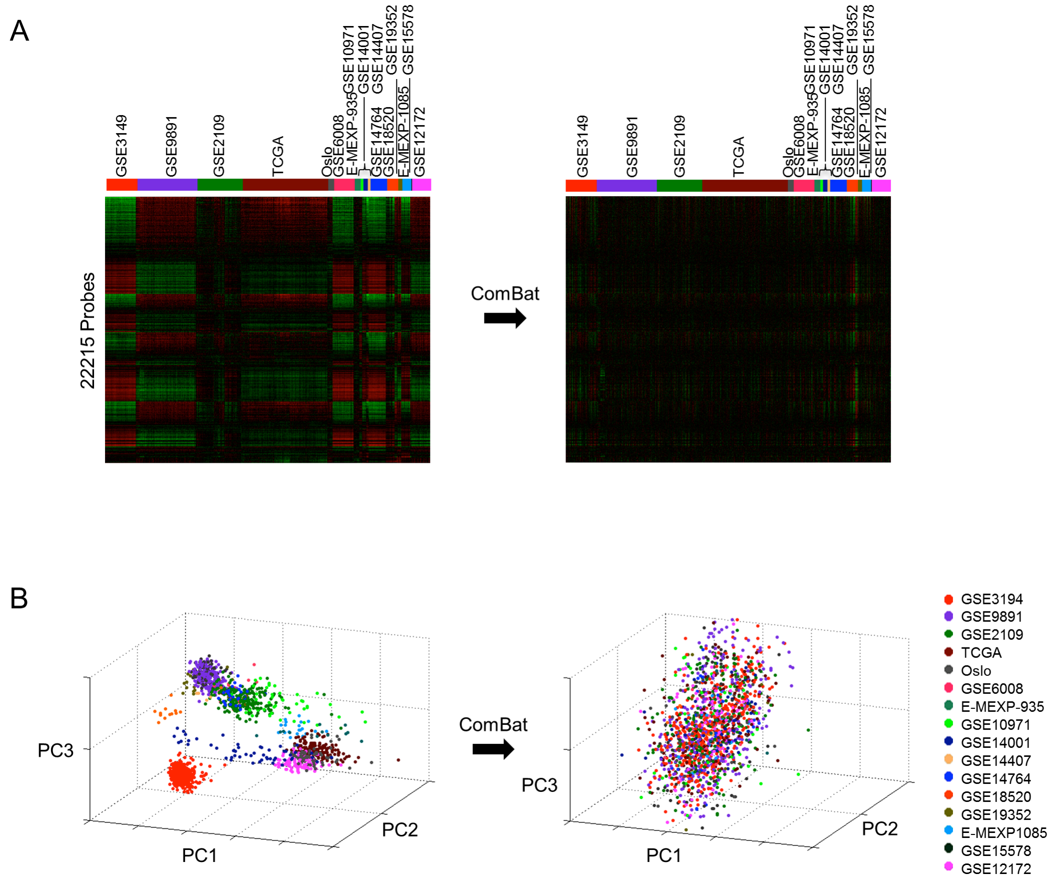

Suppl. Fig. 1. Removing the batch effect of combined expression microarray data for epithelial ovarian cancer.

A. Heatmaps showing the effect of ComBat standardisation. The samples were aligned according to 16 RMA-normalised, independent ovarian cancer gene expression data (GSE3149, GSE9891, GSE2109, TCGA, Oslo cohort, GSE6008, E-MEXP-935, GSE10971, GSE14001, GSE14407, GSE14764, GSE18520, GSE19352, E-MEX-P1085, GSE15578, and GSE12172). Combined gene expression heatmaps of 22,215 probes available on the U133A platform before (left panel) and after (right panel) ComBat standardisation. B. Effect of ComBat standardisation in principle component analysis (PCA) of 16 independent cohorts. Left panel. Prior to ComBat standardisation, the combined ovarian cancer gene expression data showed technical variations. Right panel. After ComBat standardisation of the same data, the technical variations were eliminated.

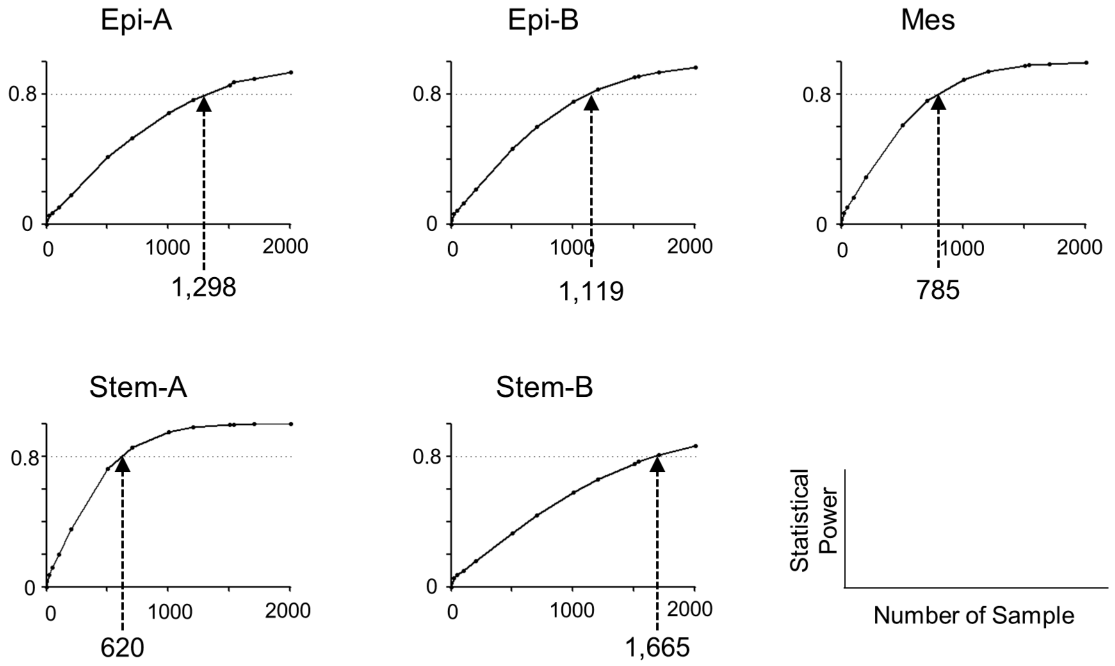

Suppl. Fig. 2. Statistical power plots.

Statistical power plots to distinguish one subtype from the others. The statistical power computation is based on the results of a *t*-test, with a significance level $\alpha = 0.05$, and on the assumption that subtype distribution is the same throughout different population sizes. Sample mean was treated as population mean. Arrows indicate the number of samples required to attain a statistical power of 0.8 for distinguishing amongst molecular subtypes. The required number of samples was approximated by fitting a cubic polynomial equation with Graphpad robust fit function. *x*-axis is the number of samples; *y*-axis is statistical power. Abbreviations: Epi-A; Epithelial-A, Epi-B; Epithelial-B, Mes; Mesenchymal, Stem-A; Stem-like-A, Stem-B; Stem-like-B.

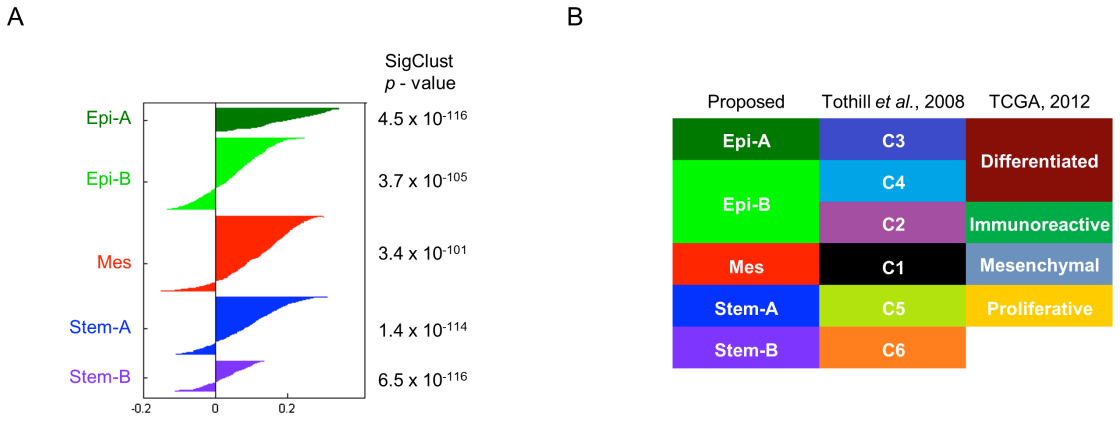

Suppl. Fig. 3. Additional information for the proposed ovarian cancer molecular subtype.

A. Silhouette plot. Clinical samples positive for the silhouette width (SW) in each subtype: Epi-A, 74.8% (101/135); Epi-B, 80.4% (315/392); Mes, 78.9% (325/412); Stem-A, 81.3% (256/315); and Stem-B, 76.7% (145/189). SigClust (Liu et al, 2008) *p*-values indicative of significance of clustering are shown to the right of the silhouette plot. B. Comparison of the proposed molecular subtype with previously published subtyping schemes for epithelial ovarian cancer by Tothill *et al.* (Tothill et al, 2008) and The Cancer Genome Atlas (TCGA) (The Cancer Genome Atlas Research Network, 2011). Note that Epi-B subtype carries an immunoreactive and a differentiated component, and also that TCGA molecular subtyping lacks Stem-B/C6 population. Abbreviations: Epi-A; Epithelial-A, Epi-B; Epithelial-B, Mes; Mesenchymal, Stem-A; Stem-like-A, Stem-B; Stem-like-B.

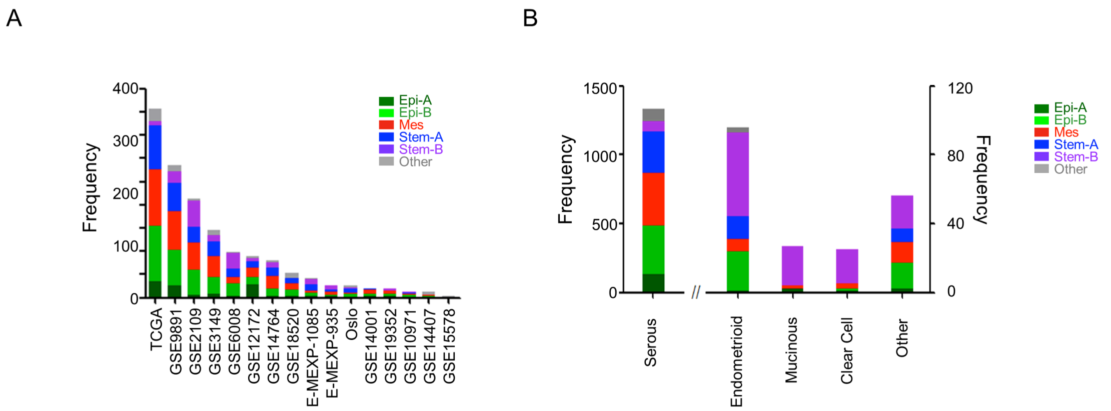

Suppl. Fig. 4. Subtype distribution

Subtype distribution by cohorts (A) and histologies (B). *X*-axis is the cohort or histology, and *y*-axis is the frequency. “Other” indicates the unclassified samples not grouped in any of the five subtypes in the initial consensus clustering analysis in Fig. 1A. Colour code: Epi-A, dark green; Epi-B, light green; Mes, red; Stem-A, blue; Stem-B, purple; Other, grey. Abbreviations: Epi-A; Epithelial-A, Epi-B; Epithelial-B, Mes; Mesenchymal, Stem-A; Stem-like-A, Stem-B; Stem-like-B.

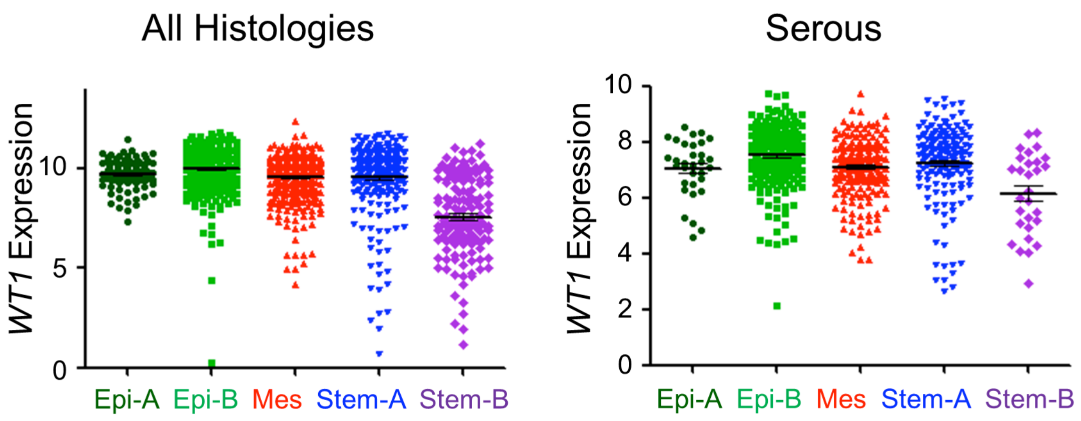

Suppl. Fig. 5. *WT1* gene expression of ovarian cancer subtype.

Left panel. Dot plots of subtypes for the gene expression level of *WT1*, a serous histology marker, in clinical ovarian cancer samples comprising serous, endometrioid, mucinous, clear cell and other histologies. Right panel. Dot plot of subtypes for *WT1* gene expression level in serous ovarian cancers only. Colour code: Epi-A, dark green; Epi-B, light green; Mes, red; Stem-A, blue; Stem-B, purple. Abbreviations: Epi-A; Epithelial-A, Epi-B; Epithelial-B, Mes; Mesenchymal, Stem-A; Stem-like-A, Stem-B; Stem-like-B.

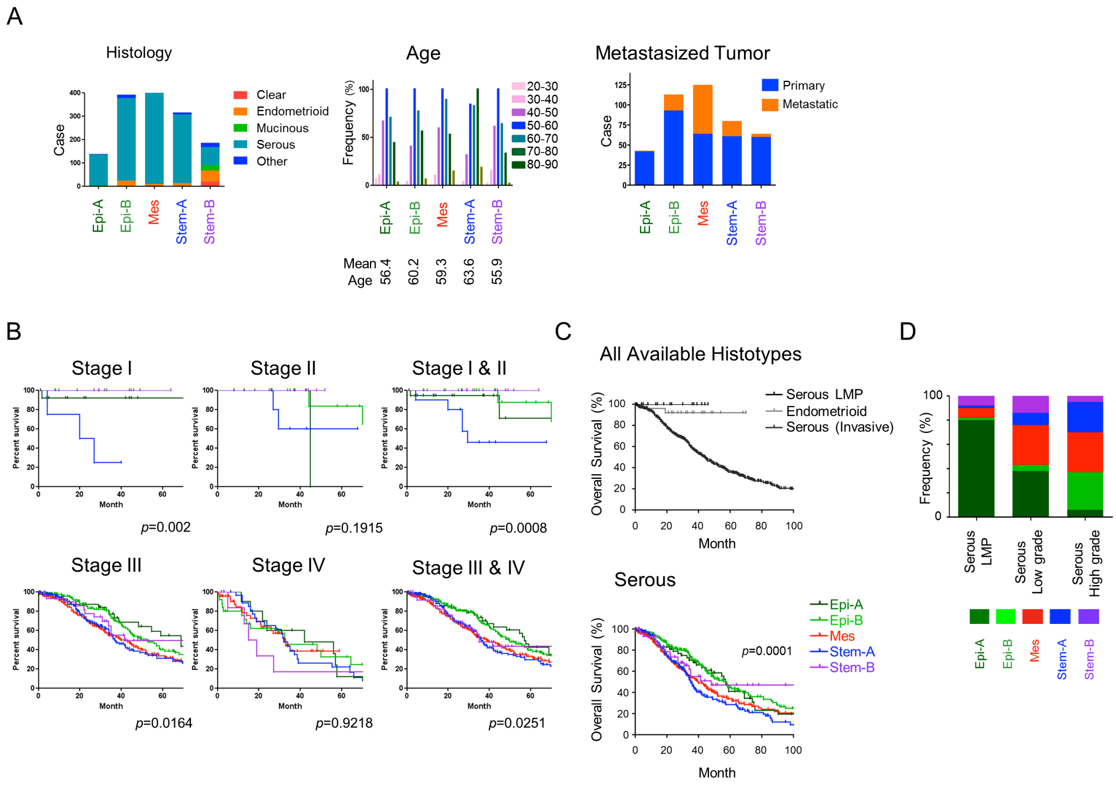

Suppl. Fig. 6. Clinicopathological characterisation of molecular subtypes.

A. Histograms showing the relationship between expression subtypes with the histotypes (left panel), age distribution (middle panel, where the mean age in years is shown underneath) and primary or metastasised tumours (right panel). Definition of metastasised tumour follows the original description in the literature (Anglesio et al, 2008; Bowen et al, 2009; Tone et al, 2008; Tothill et al, 2008). B. Kaplan-Meier survival analyses stratified by clinical stage. Note that patients with stage I or II Stem-A ovarian carcinomas have worse outcome, with statistical significance. Epi-A and Epi-B subtypes show better prognoses overall, but Stem-B cancers are no longer benign at advanced stages. C. Prognostic feature of subtypes in serous ovarian carcinomas without low malignant potential (LMP) (= high-grade serous EOCs). A total of 863 samples in this category were analysed (Epi-A; 63, Epi-B; 258, Mes; 280, Stem-A; 217, and Stem-B; 45 samples). Kaplan-Meier analyses of EOCs with all available histotypes (upper panel) and with high-grade serous histotype (lower panel) are shown. Note that the molecular sub-classification further dissects heterogeneity of high-grade serous EOCs in patient prognosis. D. Distribution of molecular subtypes in ovarian serous tumours. The overview frequency distribution as a percentage for ovarian cancer subtypes as LMP, low-

grade and high-grade serous ovarian carcinomas. *x*-axis is the subtype or histological type, whereas *y*-axis is either the frequency or frequency as a percentage. Colour code: Epi-A, dark green; Epi-B, light green; Mes, red; Stem-A, blue; Stem-B, purple. Abbreviations: Epi-A; Epithelial-A, Epi-B; Epithelial-B, Mes; Mesenchymal, Stem-A; Stem-like-A, Stem-B; Stem-like-B.

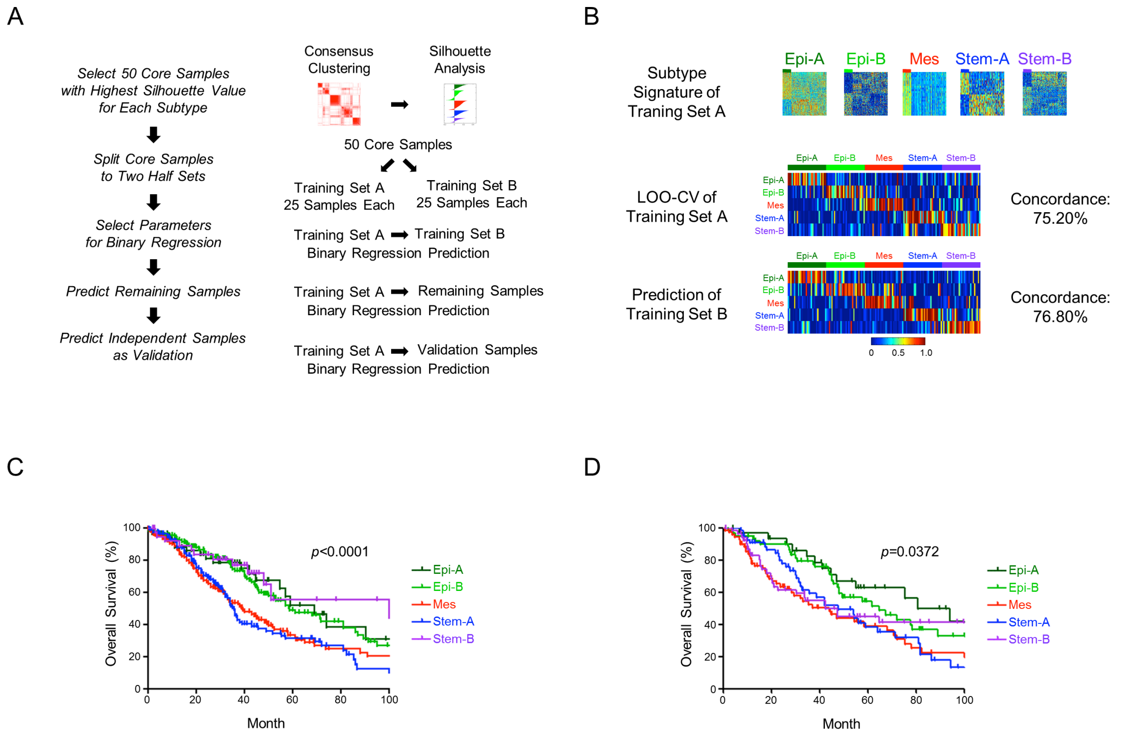

Suppl. Fig. 7. A diagnostic method to predict ovarian cancer subtype based on a Binary Regression model.

A. Scheme of diagnostic subtype prediction based on a Binary Regression (BinReg) model. For each subtype, 50 clinical samples with the highest silhouette values in Suppl. Fig. 3A were subdivided into training sets A and B, and predictive models were generated based on the results of the gene expression arrays of training set A. Multiple tests of predictions from training set A to training set B determined the best condition to perform a BinReg. The defined condition (Suppl. Materials and Methods) was used to predict the status of the remaining samples and the samples with unknown status for the subtype. B. Generation of subtype predictors. Upper panels. Gene expression heatmaps for subtype predictor. Expression of predictor genes: red = high; blue = low. Bar = 25 samples were used to generate the subtype signature. Middle panel. Heatmap for predicted probabilities of subtype status of leave-one-out cross-validation (LOO-CV) analysis. Lower panel. Heatmap for predicted probabilities of subtype status of training set B samples. Overall concordance (comparing the subtype assignment by consensus clustering against that predicted by BinReg) is shown. C. Kaplan-Meier analysis of the predicted subtype of samples not used in predictor

generation. The concordant samples with survival information (n = 811) were plotted according to the predicted subtypes. *p*-value was computed by the log-rank test. D. Kaplan-Meier analysis of 260 validation samples for which patient outcome information was supplied with predicted subtypes by BinReg. *p*-value was computed by the log-rank test. Note that the similarity of the patient outcomes between the training and validation sets was observed in the subtypes except for Stem-B (Fig. 1B,; Suppl. Figs. 7C and 8D). Abbreviations: Epi-A; Epithelial-A, Epi-B; Epithelial-B, Mes; Mesenchymal, Stem-A; Stem-like-A, Stem-B; Stem-like-B.

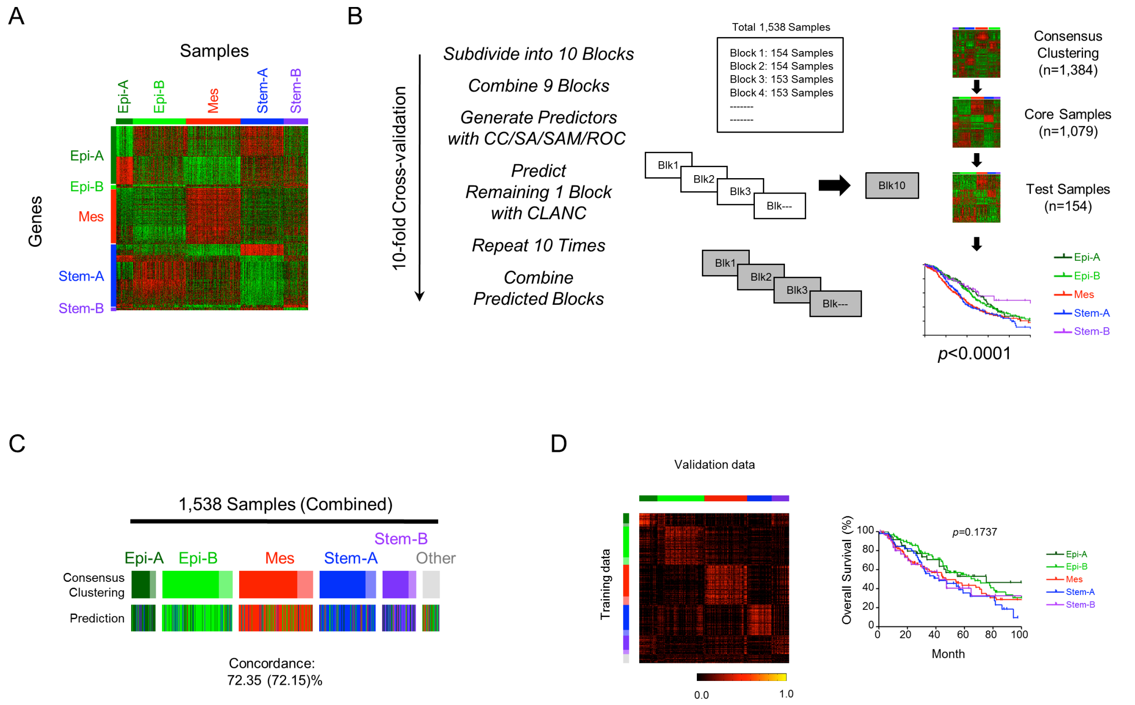

Suppl. Fig. 8. Diagnostic subtype prediction by SAM/ROC/ClaNC.

A. Gene expression heatmap of subtype-specific genes. Clinical samples with positive silhouette width (SW) values (Suppl. Fig. 3A) are aligned according to their subtype. Red = high, green = low expression. The subtype-specific genes were identified using significance analysis of microarrays (SAM) (false discovery rate; FDR q = 0%) and receiver operating characteristic (ROC) analysis (> 0.78) (Tusher et al, 2001). B. Left panels. Scheme of 10-fold cross-validation. We performed a 10-fold cross-validation analysis, in which we generated the expression signatures of 90% samples, predicted the subtype status of the remaining 10% of the samples with the signatures by classification to nearest centroids (ClaNC), and repeated these predictions 10 times (Dabney, 2006; Subramanian & Simon, 2011). Data from 1,538 epithelial ovarian cancer (EOC) samples were randomly subdivided into 10 blocks of 154 or 153 samples. Using data from 9 blocks (90% samples) (total 1,384 or 1,385 samples) as training data, subtype identification and subsequent gene selection were performed with consensus clustering (CC), silhouette analysis (SA), SAM and ROC. The remaining block (10% samples) was used as a validation set and the subtype of each sample was predicted by ClaNC. We repeated this process 10 times, combined predictions and performed Kaplan-Meier survival analysis. Right panels. An example of cross-validation (Experiment #2). Gene

expression heatmaps (red = high, green = low expression) and Kaplan-Meier survival analysis of the combined result at the final stage are shown. C. Concordance of the ClaNC prediction with the subtype status derived from consensus clustering. Coloured bar = subtype status prediction of a sample. Samples are aligned according to the subtype classification by CC and SW. Deep colour = positive SW; pale colour = samples classified to a subtype but negative SW. Concordance was computed by comparing the subtype assignment by CC of 1,538 clinical samples against the predicted subtype by ClaNC. The number in parentheses indicates the accuracy of the prediction against core samples. This 10-fold cross-validation showed an overall concordance of 72.4% for 1,538 samples. "Other" indicates the unclassified samples not grouped in any of the five subtypes in the initial consensus clustering analysis in Fig. 1A. D. Left panel. Heatmap of Spearman correlation *Rho* of the training data subtype (n = 1,538) and subtype predicted by ClaNC in the validation set comprising five independent datasets (GSE19829 [n = 28], GSE20565 [n = 95], GSE30311 [n = 67], GSE26712 [n = 185] and GSE27651 [n = 43]; total n = 418). Correlation of gene expression is computed based on the subtype signature (SAM false discovery rate = 0, and ROC = 0.78). Yellow indicates a perfect correlation (*Rho* = 1) whereas black indicates no correlation (*Rho* = 0). Right panel. Kaplan-Meier plot of subtype predicted by ClaNC in the validation set. *p*-value shown is computed by log-rank test. Note the similarity of the patient outcomes between the training and validation sets observed in the subtypes except for Stem-B (Fig. 1B; Suppl. Figs. 7C and 7D). Abbreviations: Epi-A; Epithelial-A, Epi-B; Epithelial-B, Mes; Mesenchymal, Stem-A; Stem-like-A, Stem-B; Stem-like-B.

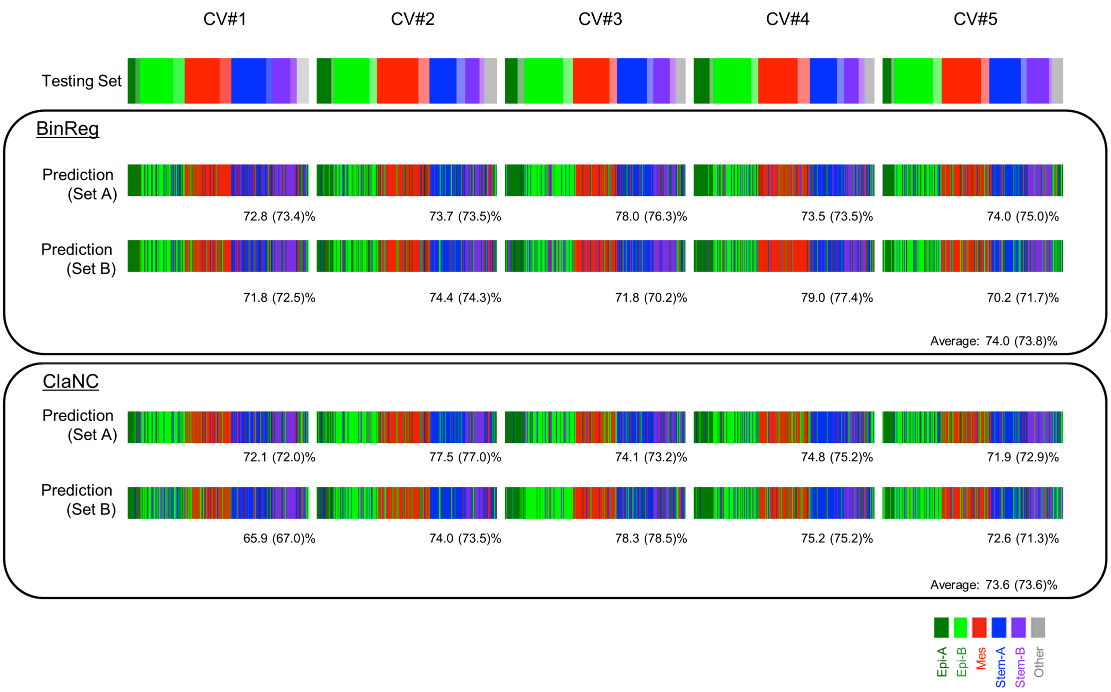

Suppl. Fig. 9. Three-way split cross-validation using BinReg and ClaNC

Five sets of three-way split cross-validations for subtype classifiers by BinReg and ClaNC using the dataset with 1,538 clinical samples are shown. The data was divided into five random sets of: training set A, (40%, n = 615); training set B, (40%, n = 615) and a testing set (20%, n = 308), such that each sample was used in the testing set exactly once to ensure balance. The classifier was subsequently built using training set A or B, and then used to predict the testing set. The five 40-40-20 cross-validation sets are aligned as columns. The top coloured bar represents the subtypes identified in consensus clustering, followed by subtype prediction by BinReg using training set A, training set B (Upper panel), and then ClaNC prediction using training set A, and training set B (Lower panel). The percentage underneath each coloured bar is the overall concordance between the predicted subtype and the consensus clustering subtype. The number in parentheses is the percentage concordance of the core samples. Average overall concordance of each classifier is given at the bottom right of each panel. “Other” indicates the unclassified samples not grouped in any of the five subtypes in the initial consensus clustering analysis in Fig. 1A. Colour code: Epi-A, dark green; Epi-B, light green; Mes, red; Stem-A, blue; Stem-B, purple; Other, grey. Abbreviations: Epi-A;

Epithelial-A, Epi-B; Epithelial-B, Mes; Mesenchymal, Stem-A; Stem-like-A, Stem-B; Stem-like-B.

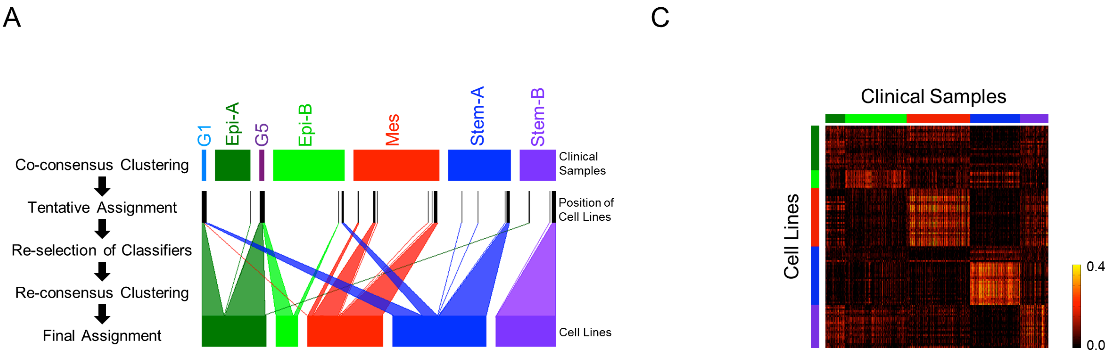

B

Epithelial-A

JHOC-7 (K)
JHOC-8 (K)
JHOC-9 (K)
JHOS-2 (K)
KOC-5c (K)
OAW42 (S)
OC 316 (N)
OV90 (N)
OVCA429 (D, K, S)
OVCA433 (D, K, S)
OVCAR-8 (D, K, S)
OVISE (K)
PEO1 (D, K, S)
PEO4 (D)
RMG-I (K)
RMG-II (K)
SKOV-3 (N)
SKOV-8 (D)
TAYA (K)
TOV-21G (N)
UWB1.289 (S)

Epithelial-B

Caov-3 (D, K, N, S)
JHOS-3 (K)
OCC1 (N)
OMC3 (K)
OVCA432 (D, K, S)

Mesenchymal

59M (N)
DOV 13 (D, N, S)
DOV 13A (D)
DOV 13B (D)
EFO-21 (S)
EFO-27 (N)
ES2 (N)
HEY (D, K)
HeyA8 (D)
HeyC2 (D)
JHOC-5 (K)
JHOM-1 (K)
OAW28 (S)
OV56 (S)
OV7 (S)
ovary1847 (D, K, S)
OVCA432 (N)
OVCAR-3 (N)
OVTOKO (K)
PA-1 (N)
PEO1 (N)
SKOV-3 (D, K, S)
TYK-nu (D, K, S)
TYK-nu cisR (D)
UPN251 (N)

Stem-A

A2780 (D, K, N, S)
A2780 cisR (D)
CHI (D, S)
CHI cisR (D)
COLO720E (S)
FU-OV-1 (D, S)
HEY (N)
IGROV-1 (D, K, S)
IOSE29 (N)
JHOS-2 (S)
JHOS-4 (K)
KOC-7C (K)
M41 (D)
M41 cisR (D)
OAW28 (N)
OAW42 (N, S)
OV17R (S)
OVCA420 (N)
OVCA433 (N)
OVCAR-10 (D, S)
OVCAR-3 (D)
OVCAR-3 (K, S)
OVCAR-8 (N)
PA-1 (D)
RMG-IV (K)
SKOV-4 (D)
SKOV-6 (D)
SW626 (N)
TOV-112D (N, S)
TOV-21G (D, K)

Stem-B

A2008 (D, K, S)
BG1 (D)
C13 (D, S)
Caov-2 (D, S)
JHOM-2B (K)
MCAS (D, K)
OC314 (N)
OC315 (N)
OV2008 (D, S)
OV90 (D, S)
OV90 (K)
OVCA420 (D, K, N, S)
OVCAR-2 (D, S)
OVCAR-5 (D, N, S)

Suppl. Fig. 10. Identification of cell line subtype status.

A. A schematic presentation of subtype classification of cell lines by consensus clustering (CC). To identify the cell-line counterparts of ovarian tumours, data for a total of 142 cell lines (Duke: GSE25429 [n = 42], Kyoto: GSE29175 [n = 37], National Laboratory: E-TABM-254 [n = 29] and Singapore: GSE28724 [n = 34]; 60 cell lines were redundant in their names) were analysed together with 1,142 core tumour samples in CC using the same gene set from Suppl. Fig. 8A. Co-clustering subdivided the cell lines into 7 clusters (G1: 21, G5: 23, Epi-A: 1, Epi-B: 12, Mes: 33, Stem-A: 24 and Stem-B: 28 cell lines) and, of those clusters, two (G1 and G5) predominantly comprised cell lines. Because the expression signature for *in vitro* cultured ovarian cell-line subtype might not be exactly matched with that of clinical tumours, we performed an additional round of CC purely relying on the re-selected cell-line classifiers based on the first-time subgrouping result (data not shown). This two-time consensus clustering finally yielded five subtypes for the cell lines (Epi-A: 29, Epi-B: 10, Mes: 34, Stem-A: 42 and Stem-B: 27 cell lines) that were unambiguously supported by

similarity matrices and the silhouette values (Fig. 2A). Upper coloured bars = subtype status of clinical samples after the first CC. Middle black bars = position of the cell line sample with clinical tumour samples in the first clustering analysis. Lower coloured bars = cell line classes. Pale triangles = relationship between the first and the second classifications. B. Cell line names in the classification. Data sources of cell lines are Duke University (D), Kyoto University (K), National University of Singapore (S) and Lawrence Berkeley National Laboratory (N). Many cell lines in the Kyoto and Singapore collections are derived from the Duke collection, while the National Laboratory collection is totally independent of the Kyoto, Duke and Singapore collections. Cell lines with a negative silhouette width in Fig. 2A are shown in grey font. The cell-line classification was confirmed across the different cohorts. Note that the Kyoto data included duplicated arrays for the HEY cell line. Also note that the two-round CC method provides consistent subtype assignments for the biological replicates across the 28 cell lines derived from the same source (Duke, Kyoto, and Singapore). C. Heatmap of Spearman correlation *Rho* of the clinical tumour subtype (n = 1,538) and ovarian cancer cell line subtype (n = 142). Correlation of gene expression is computed based on the subtype signature (SAM false discovery rate = 0, and ROC = 0.82) from ovarian cancer cell lines. Yellow indicates a perfect correlation (*Rho* = 1) whereas black indicates no correlation (*Rho* = 0). Abbreviations: Epi-A; Epithelial-A, Epi-B; Epithelial-B, Mes; Mesenchymal, Stem-A; Stem-like-A, Stem-B; Stem-like-B.

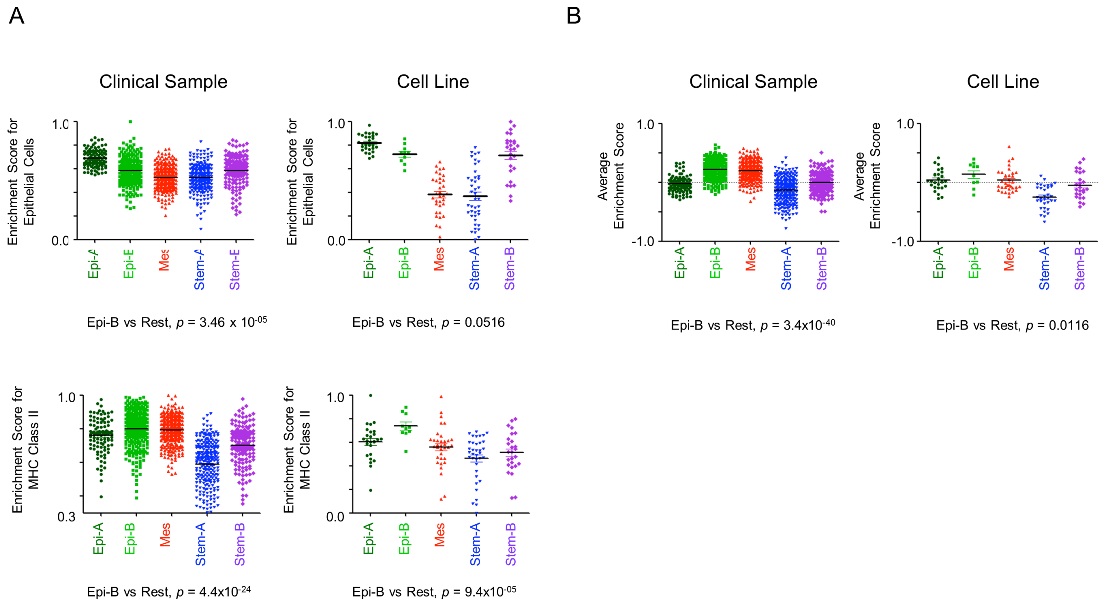

Suppl. Fig. 11. Surface marker and interferon signature of Epi-B cell lines.

A. Upper panel. Enrichment scores for epithelial cell markers of the five ovarian carcinoma subtypes for clinical samples and cell lines. The gene set was derived from expression microarrays of ovarian cancer cell lines with *CDH1* and *CDH2* immunohistochemistry staining pattern (SAM false discovery rate = 0, ROC = 0.85), consisting of known epithelial cell markers including *DDR1*, *KRT8*, *KRT18*, *CDH1*, *CDH3*, *CLDN3*, *CLDN4*, and *EPCAM*. Lower panel. Enrichment scores for MHC class IIs (comprising genes such as *HLA-DM*, *DO*, *DP*, *DQ*, and *DR*) for the five molecular subtypes in ovarian carcinoma. B. Average enrichment score of interferon signatures found in MSigDb v3.0 (Suppl. Table 6) and Gatza *et al*. (Gatza et al, 2010) of ovarian carcinoma subtype. For A and B panels, the left panel contains a dot plot of the clinical samples, whereas the right panel contains a dot plot of the cell lines. The *p*-value underneath each dot plot provides the significance level of distinguishing Epi-B from the others and is computed with Mann-Whitney *U*-test. Colour code: Epi-A, dark green; Epi-B, light green; Mes, red; Stem-A, blue; Stem-B, purple. Abbreviations: Epi-A; Epithelial-A, Epi-B; Epithelial-B, Mes; Mesenchymal, Stem-A; Stem-like-A, Stem-B; Stem-like-B.

A

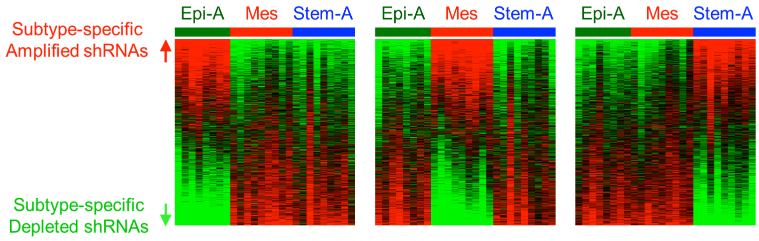

B

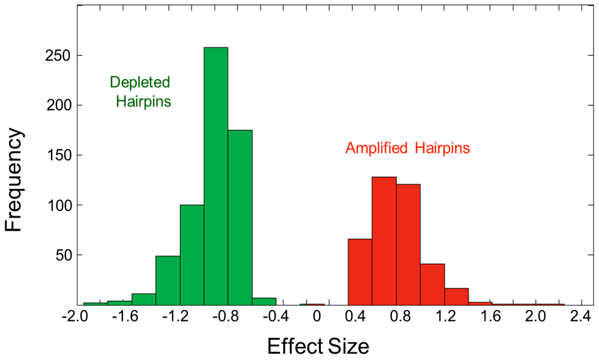

Suppl. Fig. 12. Subtype-specific amplified or depleted hairpins.

A. Subtype-specific cell growth-relevant genes. Heatmaps of centred and normalised copy numbers of 14 cell lines for the whole hairpins (57,168 hairpins) retrieved after next-generation sequencing analysis. The results are compiled from two independent screens. Quadruplicates of three cell lines (OVCA433 [Epi-A], HeyA8 [Mes] and PA-1 [Stem-A]) were assayed in the initial screen, while the other screen regarded one experimental replicate of 14 different cell lines (4 Epi-A: OVCA429, OVCAR-8, OVCA433, PEO1; 5 Mes: ovary1847, HEY, HeyA8, HeyC2, SKOV-3; and 5 Stem-A: A2780, CH1, PA-1, SKOV-4, SKOV-6). The cell lines were sorted according to their subtypes (Epi-A, Mes then Stem-A), whereas the hairpin copy numbers were sorted according to hairpin score in the RIGER analysis (Luo et al, 2008), with which binary comparisons were performed for each subtype to obtain subtype-specific cell growth determinant genes. Note that a clear genome-wide distinctive pattern of hairpins across subtypes was detected. The relatively amplified hairpins (red) putatively target the genes that have suppressive effects on cell growth under conventional culture conditions, while genes targeted by the relatively depleted shRNAs

(green) may have growth-promoting effects on cells with a given subtype. Red = higher; green = lower copy number counts. B. Effect-size distribution of the subtype-specific amplified or depleted hairpins from the three binary comparisons. *x*-axis is the effect size, *y*-axis is the frequency. Abbreviations: Epi-A; Epithelial-A, Mes; Mesenchymal, Stem-A; Stem-like-A.

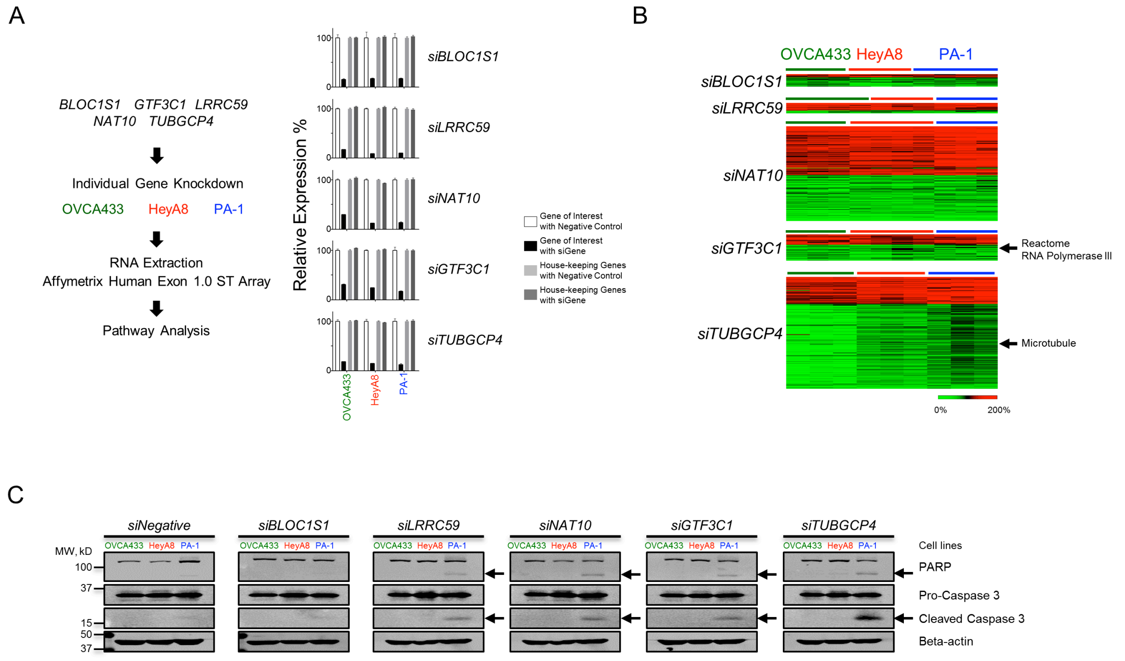

Suppl. Fig. 13. Pathways affected by silencing PA-1-specific genes.

A. Left panel. A scheme of the pathway analysis after silencing each PA-1-specific gene in OVCA433, HeyA8 and PA-1 cells using siRNA. Right panel. Effect of silencing each of the five PA-1 genes on the relative gene expression in the three cell lines (OVCA433, HeyA8, PA-1). Bar plots indicate the expression of genes of interest with Non-Targeting siRNA (negative control) (white bar), silencing a gene of interest (si"Gene"; black bar), averaged expression of house-keeping genes (*ACTB, B2M, GAPDH, HPRT1, PGK1, PGK2, PPP1CA, RPL13A, TBP, TFRC*) with Non-Targeting Pool siRNA (negative control) (light grey bar) and silencing a gene of interest (dark grey bar). Error bar indicates SEM of triplicate experiments. B. Common pathways in response to knockdown of the five PA-1 genes. Heatmaps show the relative change in pathway activities of ss-GSEA scores (si"Gene" versus siNon-Targeting negative control). Positions of *Reactome of RNA polymerase* and *Microtubule* are indicated to the right. Red = increased activity; green = decreased activity. The bar on top of the heatmap indicates triplicates or quadruplicates of OVCA433, HeyA8 and PA-1 cells. Green = OVCA433 (Epi-A), Red = HeyA8 (Mes), Blue = PA-1 (Stem-A). C. Detection of apoptotic activity initiated by the five PA-1 gene knockdowns. The five PA-1 selective genes were silenced individually by siRNA in OVCA433, HeyA8 and PA-1 and examined for the

presence of apoptotic activity by immunoblotting for cleaved PARP and Caspase 3. Arrows indicate cleavage of PARP and Caspase 3. Suppression of *LRRC59, NAT10, GTF3C1 and TUBGCP4* was linked with the induction of apoptosis only in PA-1 cells, albeit a slight apoptotic response was also detected using the siRNA negative control in PA-1. Abbreviations: Epi-A; Epithelial-A, Mes; Mesenchymal, Stem-A; Stem-like-A.

Supplemental Tables

Please refer to the Excel file.

Supplemental Materials and Methods

Principal component analysis

Principal component analysis (PCA) was performed on the combined dataset before and after ComBat standardisation to ensure analysis on the combined dataset was not biased by the presence of technical variations derived from non-biological effects. Expression data of all the probes were used to calculate the principal components, and then the first three principal components comprising less than ~97% variance were visualised in the 3-dimensional scatter plot by Matlab (ver. 7.8.0).

Statistical power analysis

Statistical power is the probability of rejecting the null hypothesis when the alternative hypothesis is true. A statistical power analysis for distinguishing among the subtypes was performed as follows: First, we computed the subtype distribution of the 1,538 ovarian carcinoma [Epi-A: 135 (8.8%), Epi-B: 392 (25.5%), Mes: 412 (26.8%), Stem-A: 315 (20.5%), Stem-B: 189 (12.3%), unclassified (5.9%)]. Second, for each gene, we estimated the population mean of a subtype ($\mu_{subtype}$) and the population mean of other subtypes ($\mu_{other\text{-}subtype}$). Last, we computed the statistical power for two-sided *t*-test using Matlab for each gene, with significance set at $\alpha = 0.05$. We repeated the procedure for the different number of samples assuming that the subtype distribution is consistent. The statistical power plot for each subtype is given in Suppl. Fig. 2.

Predictive modelling and validation by BinReg

The BinReg parameters determined from training sets A and B for molecular subtype predictive modelling are given in Suppl. Table 17.

Predictive modelling and validation by ClaNC

Silhouette analysis was performed using Matlab (ver. 7.8.0) to identify core samples that best represent their subtypes with a positive silhouette width. Significance analysis of microarrays

(SAM) and receiver operating characteristic (ROC) analysis were applied to determine the marker genes for each subtype and to assess their capability for distinguishing one subtype from the others (Tusher et al, 2001). A false discovery rate of zero and area under the curve (Pejovic et al, 2009) threshold of >0.78 (up-regulated in the subtype) or <0.22 (down-regulated in the subtype) were used to filter out non-significant genes for SAM and for ROC, respectively. Based on these marker genes, we applied classification of microarrays to nearest centroids (ClaNC) to generate signatures for each subtype, and subsequently the subtype predictive model of the clinical samples (Dabney, 2006). In order to validate the subtype prediction, we adopted for a 10-fold cross-validation to provide a sufficient estimation of the predictive model performance. In the 10-fold cross-validation, the 1,538 epithelial ovarian cancer samples were randomly partitioned into 10 sets, each comprising 153-154 samples (Subramanian & Simon, 2011). One set was used as a validation set (to be predicted), whereas the other 9 sets (1,384 or 1,385 samples) were used to build the predictive model. This process was repeated 10 times such that each set was used as the validation set exactly once. This method minimised bias introduced by the sample order and distribution when assessing the predictive model. Subtype predictions of all the validation sets were combined and compared against the subtype assignment by consensus clustering on all of the 1,538 samples.

Three-way split cross-validation using BinReg and ClaNC

To provide additional assessment on BinReg and ClaNC for ovarian cancer molecular subtype predictors, we performed five random 40-40-20 cross-validations using the two methods, where two mutually exclusive sets comprising 40% (n = 615) of the 1,538 ovarian cancer samples were used as either training set A or B to model a predictor, and the remaining 20% (n = 308) of the samples not in training sets A and B were used as a testing set to assess the predictor performance. To ensure balance in predictor assessment, each sample was used in the testing sample exactly once in the five random 40-40-20 data division. Subsequently, we identified molecular subtypes independently from training set A or B using consensus

clustering (with most varying genes), silhouette width analysis, and then build BinReg or ClaNC predictors using core samples, as described in the section "Predictive modelling and validation by BinReg/ClaNC" of Materials and Methods and Suppl. Materials and Methods. The predictive model from training set A or B was then used to predict the subtype of the samples in the testing set. Concordance was measured by comparing predicted subtype with the original classification from the consensus clustering using all the 1,538 samples. The result is shown in Suppl. Fig. 9.

Cell line panel

The cell-line panel used in the study was a mixture of histology according to the publicly available descriptions (Suppl. Table 11). Microarray analyses for the gene expression of 142 ovarian cancer cell lines were combined from four independent datasets and analysed: (1) Duke University (n = 42: A2008, A2780 cisR, A2780, BG1, C13, Caov-2, Caov-3, CH1, CH1 cisR, DOV 13, FU-OV-1, HEY, HeyA8, HeyC2, IGROV-1, M41, M41 cisR, MCAS, NOSE-06, NOSE-07, OV2008, OV90, ovary1847, OVCA420, OVCA429, OVCA432, OVCA433, OVCAR-10, OVCAR-2, OVCAR-3, OVCAR-5, OVCAR-8, PA-1, PEO1, PEO4, SKOV-3, SKOV-4, SKOV-6, SKOV-8, TOV-21G, TYK-nu, TYK-nu cisR); (2) Kyoto University (n = 37: A2008, A2780, Caov-3, HEY, IGROV-1, JHOC-5, JHOC-7, JHOC-8, JHOC-9, JHOM-1, JHOM-2B, JHOS-2, JHOS-3, JHOS-4, KOC-5c, KOC-7c, MCAS, OMC3, OV90, ovary1847, OVCA420, OVCA429, OVCA432, OVCA433, OVCAR-3, OVCAR-8, OVISE, OVK-18, OVTOKO, PEO1, RMG-I, RMG-II, RMG-V, SKOV-3, TAYA, TOV-21G, TYK-nu); (3) National University of Singapore (n = 34: A2008, A2780, C13, Caov-2, Caov-3, CH1, COLO720E, DOV 13, EFO-21, FU-OV-1, IGROV-1, JHOS-2, OAW28, OAW42, OV17R, OV2008, OV56, OV7, OV90, ovary1847, OVCA420, OVCA429, OVCA432, OVCA433, OVCAR-10, OVCAR-2, OVCAR-3, OVCAR-5, OVCAR-8, PEO1, SKOV-3, TOV-112D, TYK-nu, UWB1.289); and (4) Lawrence Berkeley National Laboratory (n = 29: 59M, A2780, Caov-3, DOV 13, EFO-27, ES-2, HEY, IOSE29, OAW28, OAW42, OC314, OC315, OC316, OCC1, OV90, OVCA420, OVCA429,

OVCA432, OVCA433, OVCAR-3, OVCAR-5, OVCAR-8, PA-1, PEO1, SKOV-3, SW626, TOV-112D, TOV-21G, UPN251). Out of these 142 cell lines, 28 cell lines originated from the same source (Duke, Kyoto, and National University of Singapore) and served as biological replicates. These 28 cell lines are described in Suppl. Table 9.

Genome-wide RNAi screens for subtype-specific proliferation genes

To assess the reliability of our shRNA screen, we performed RIGER analysis to obtain hairpins that correlated with the *TP53* status of the 14 cell lines. The result shows that our systematic functional screening was able to deliver known *TP53*-downstream genes associated with a *TP53* genotype of the cultured cell lines, with statistical significance (data not shown). Subsequently, the same method was applied to identify hairpins specifically relevant for cell growth in a molecular subtype.

A comparison of the Cheung *et al.* (Cheung et al, 2011) dataset with ours was not feasible because of different experimental designs (i.e., at the endpoint of the screen, we compared the abundance of integrated shRNA sequences across the different molecular subtypes, while Cheung *et al.* compared the relative abundance to the initial shRNA pool across different cell line lineages) and detection platforms (next-generation sequencing versus microarray, respectively).

Pathway analysis in silencing PA-1-specific genes

Dharmacon SMART pool ON-TARGET*plus* siRNA (Thermo Fisher Scientific, Lafayette, CO) was used to silence the genes in OVCA433 (Epi-A), HeyA8 (Mes), and PA-1 (Stem-A). ON-TARGET*plus* Non-Targeting Pool (#D-001810-10-20) was used as a negative control. siRNA reverse transfections were performed in triplicate in a 6-well plate under the following conditions: OVCA433, 32,000 cells with 3.0 µl of DF1 (T-2001); HeyA8, 19,000 cells with 0.7 µl of DF4 (T-2004); PA-1, 18,000 cells with 2.5 µl of DF2 (T-2002, Thermo Fisher Scientific). After 96-hour incubation, RNA was extracted (#74106, RNeasy Mini Kit, Qiagen, Hilden, Germany), and assayed for Affymetrix Human Exon 1.0 ST arrays (Affymetrix, Santa

Clara, CA). Fifty-eight microarray data were obtained, including duplicates to bridge four different microarray batches. The fold-change in the ss-GSEA score of siRNA treatment with a gene of interest versus that with negative control siRNA was computed. The gene sets with a standard deviation <0.1 and maximum absolute fold-change of >0.3 across all three cell lines were denoted as commonly altered gene sets. These gene sets are shown in Suppl. Table 14.

Supplemental References

Anglesio MS, Arnold JM, George J, Tinker AV, Tothill R, Waddell N, Simms L, Locandro B, Fereday S, Traficante N et al (2008) Mutation of ERBB2 provides a novel alternative mechanism for the ubiquitous activation of RAS-MAPK in ovarian serous low malignant potential tumors. Mol Cancer Res 6**:** 1678-1690

Bowen NJ, Walker LD, Matyunina LV, Logani S, Totten KA, Benigno BB, McDonald JF (2009) Gene expression profiling supports the hypothesis that human ovarian surface epithelia are multipotent and capable of serving as ovarian cancer initiating cells. BMC Med Genomics 2**:** 71

Boyer CM, Dawson DV, Neal SE, Winchell LF, Leslie DS, Ring D, Bast RC, Jr. (1989) Differential induction by interferons of major histocompatibility complex-encoded and non-major histocompatibility complex-encoded antigens in human breast and ovarian carcinoma cell lines. Cancer research 49**:** 2928-2934

Calza S, Hall P, Auer G, Bjohle J, Klaar S, Kronenwett U, Liu ET, Miller L, Ploner A, Smeds J et al (2006) Intrinsic molecular signature of breast cancer in a population-based cohort of 412 patients. Breast cancer research : BCR 8**:** R34

Cheung HW, Cowley GS, Weir BA, Boehm JS, Rusin S, Scott JA, East A, Ali LD, Lizotte PH, Wong TC et al (2011) Systematic investigation of genetic vulnerabilities across cancer cell lines reveals lineage-specific dependencies in ovarian cancer. Proceedings of the National Academy of Sciences of the United States of America 108**:** 12372-12377

Dabney AR (2006) ClaNC: point-and-click software for classifying microarrays to nearest centroids. Bioinformatics 22**:** 122-123

Fava F, Raynaud-Messina B, Leung-Tack J, Mazzolini L, Li M, Guillemot JC, Cachot D, Tollon Y, Ferrara P, Wright M (1999) Human 76p: A new member of the gamma-tubulin-associated protein family. The Journal of cell biology 147**:** 857-868

Fodde R (2009) The stem of cancer. Cancer Cell 15**:** 87-89

Freedman RS, Kudelka AP, Kavanagh JJ, Verschraegen C, Edwards CL, Nash M, Levy L, Atkinson EN, Zhang HZ, Melichar B et al (2000) Clinical and biological effects of intraperitoneal injections of recombinant interferon-gamma and recombinant interleukin 2 with or without tumor-infiltrating lymphocytes in patients with ovarian or peritoneal carcinoma. Clinical cancer research : an official journal of the American Association for Cancer Research 6**:** 2268-2278

Gatza ML, Lucas JE, Barry WT, Kim JW, Wang Q, Crawford MD, Datto MB, Kelley M, Mathey-Prevot B, Potti A et al (2010) A pathway-based classification of human breast cancer. Proceedings of the National Academy of Sciences of the United States of America 107**:** 6994-6999

Guan Y, Kuo WL, Stilwell JL, Takano H, Lapuk AV, Fridlyand J, Mao JH, Yu M, Miller MA, Santos JL et al (2007) Amplification of PVT1 contributes to the pathophysiology of ovarian and breast cancer. Clinical cancer research : an official journal of the American Association for Cancer Research 13**:** 5745-5755

Haibe-Kains B, Desmedt C, Loi S, Culhane AC, Bontempi G, Quackenbush J, Sotiriou C (2012) A three-gene model to robustly identify breast cancer molecular subtypes. Journal of the National Cancer Institute 104: 311-325

Kovelman R, Roeder RG (1992) Purification and characterization of two forms of human transcription factor IIIC. The Journal of biological chemistry 267: 24446-24456

Lawrenson K, Gayther SA (2009) Ovarian cancer: a clinical challenge that needs some basic answers. PLoS medicine 6: e25

Liu Y, Hayes DN, Nobel A, Marron JS (2008) Statistical Significance of Clustering for High-Dimension, Low-Sample Size Data. Journal of the American Statistical Association 103: 1281-1293

Lowe AW, Olsen M, Hao Y, Lee SP, Taek Lee K, Chen X, van de Rijn M, Brown PO (2007) Gene expression patterns in pancreatic tumors, cells and tissues. PloS one 2: e323

Luo B, Cheung HW, Subramanian A, Sharifnia T, Okamoto M, Yang X, Hinkle G, Boehm JS, Beroukhim R, Weir BA et al (2008) Highly parallel identification of essential genes in cancer cells. Proceedings of the National Academy of Sciences of the United States of America 105: 20380-20385

Mantovani A, Allavena P, Sica A, Balkwill F (2008) Cancer-related inflammation. Nature 454: 436-444

Matsumura N, Huang Z, Mori S, Baba T, Fujii S, Konishi I, Iversen ES, Berchuck A, Murphy SK (2011) Epigenetic suppression of the TGF-beta pathway revealed by transcriptome profiling in ovarian cancer. Genome Res 21: 74-82

Moritz M, Braunfeld MB, Sedat JW, Alberts B, Agard DA (1995) Microtubule nucleation by gamma-tubulin-containing rings in the centrosome. Nature 378: 638-640

Moritz M, Zheng Y, Alberts BM, Oegema K (1998) Recruitment of the gamma-tubulin ring complex to Drosophila salt-stripped centrosome scaffolds. The Journal of cell biology 142: 775-786

Pejovic T, Pande NT, Mori M, Mhawech-Fauceglia P, Harrington C, Mongoue-Tchokote S, Dim D, Andrews C, Beck A, Tarumi Y et al (2009) Expression profiling of the ovarian surface kinome reveals candidate genes for early neoplastic changes. Transl Oncol 2: 341-349

Perou CM, Jeffrey SS, van de Rijn M, Rees CA, Eisen MB, Ross DT, Pergamenschikov A, Williams CF, Zhu SX, Lee JC et al (1999) Distinctive gene expression patterns in human mammary epithelial cells and breast cancers. Proceedings of the National Academy of Sciences of the United States of America 96: 9212-9217

Prat A, Parker JS, Karginova O, Fan C, Livasy C, Herschkowitz JI, He X, Perou CM (2010) Phenotypic and molecular characterization of the claudin-low intrinsic subtype of breast cancer. Breast cancer research : BCR 12: R68

Subramanian J, Simon R (2011) An evaluation of resampling methods for assessment of survival risk prediction in high-dimensional settings. Stat Med 30: 642-653

The Cancer Genome Atlas Research Network T (2011) Integrated genomic analyses of ovarian carcinoma. Nature 474: 609-615

Tone AA, Begley H, Sharma M, Murphy J, Rosen B, Brown TJ, Shaw PA (2008) Gene expression profiles of luteal phase fallopian tube epithelium from BRCA mutation carriers resemble high-grade serous carcinoma. Clinical cancer research : an official journal of the American Association for Cancer Research 14**:** 4067-4078

Tothill RW, Tinker AV, George J, Brown R, Fox SB, Lade S, Johnson DS, Trivett MK, Etemadmoghadam D, Locandro B et al (2008) Novel molecular subtypes of serous and endometrioid ovarian cancer linked to clinical outcome. Clinical cancer research : an official journal of the American Association for Cancer Research 14**:** 5198-5208

Tusher VG, Tibshirani R, Chu G (2001) Significance analysis of microarrays applied to the ionizing radiation response. Proceedings of the National Academy of Sciences of the United States of America 98**:** 5116-5121

Verhaak RG, Tamayo P, Yang JY, Hubbard D, Zhang H, Creighton CJ, Fereday S, Lawrence M, Carter SL, Mermel CH et al (2013) Prognostically relevant gene signatures of high-grade serous ovarian carcinoma. The Journal of clinical investigation 123**:** 517-525

Virtanen C, Ishikawa Y, Honjoh D, Kimura M, Shimane M, Miyoshi T, Nomura H, Jones MH (2002) Integrated classification of lung tumors and cell lines by expression profiling. Proceedings of the National Academy of Sciences of the United States of America 99**:** 12357-12362
